# Carabrone Attenuates Metabolic Dysfunction–Associated Steatohepatitis by Targeting STAT3 in Mice

**DOI:** 10.1002/mco2.70145

**Published:** 2025-03-10

**Authors:** An Pan, Jiaming Jin, Yuze Wu, Qiang Zhang, Huanhuan Chen, Yang Hu, Wen Xiao, Anqi Shi, Yang Yang, Lina Jiang, Minghui Tan, Junwei Wang, Lihong Hu

**Affiliations:** ^1^ State Key Laboratory on Technologies for Chinese Medicine Pharmaceutical Process Control and Intelligent Manufacture Nanjing University of Chinese Medicine Nanjing China; ^2^ Jiangsu Key Laboratory for Functional Substance of Chinese Medicine School of Pharmacy Nanjing University of Chinese Medicine Nanjing China; ^3^ School of Life Science and Bioengineering Jining University Jining China

**Keywords:** carabrone, chemoproteomic profiling, MASH, STAT3

## Abstract

Metabolic dysfunction–associated steatohepatitis (MASH) has become one of the most common progressive liver diseases worldwide, but effective treatment options are severely unmet. Carabrone, a sesquiterpene lactone from the traditional Chinese herb *Carpesium abrotanoides *L., shows various pharmacological properties, whereas its effect on the improvement of MASH and the underlying mechanisms have not yet been reported. In this work, we revealed for the first time the beneficial effect of carabrone on MASH, including reducing liver lipid accumulation, inflammatory cell infiltration, and fibrosis in multiple diet‐induced mice. Carabrone also alleviated lipid accumulation and inflammation in palmitic acid/oleic acid–stimulated hepatocytes. Mechanically, we identified signal transducers and transcriptional activator 3 (STAT3) as a key target of carabrone for treating MASH through quantitative chemical proteomic analysis, as well as the verification by the overexpression of STAT3 in vivo and in vitro. Further studies demonstrated that carabrone blocks MASH progression by inhibiting the activation of STAT3. More importantly, a new carabrone derivative **CA‐21** with stronger anti‐MASH activity and affinity for STAT3 was discovered through rational structural modification. Taken together, our findings suggest that carabrone and **CA‐21** could be developed as promising drug candidates for MASH treatment.

## Graphical abstract

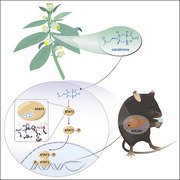

## 1 Introduction

Metabolic dysfunction associated–liver disease (MAFLD) has become the most common chronic liver disease worldwide, encompassing metabolic dysfunction–associated fatty liver (MAFL) and the more severe metabolic dysfunction–associated steatohepatitis (MASH). MASH is characterized by steatosis and inflammation, and if left untreated, it can progress to cirrhosis, liver failure, and even liver cancer. Estimates suggest that 30% of the global population is affected by MAFLD, and of these, 20% may advance to MASH, imposing a substantial burden on global health [1, 2].

Recent research has revealed various pathological mechanisms of MASH, with hepatocyte injury, steatosis, inflammation, and varying degrees of fibrosis considered to be the main pathogenic factors [3, 4]. The diagnosis of MAFLD and MASH is usually based on one or more of the following criteria: overweight/obesity, type 2 diabetes, or metabolic disorders. The presence of other liver conditions, such as alcoholic liver disease and viral hepatitis, does not require diagnosis for MASH [5]. A definitive clinical diagnosis of MAFLD and MASH usually requires a liver biopsy. Additionally, imaging techniques such as magnetic resonance imaging (MRI), computed tomography (CT), ultrasound (US), and elastography are effective tools for assessing fat deposition and fibrosis [6].

Despite decades of efforts in new drug development for MASH, clinical trials have often encountered setbacks due to adverse side effects or insufficient efficacy in achieving clinical endpoints. Only one new drug, resmetirom, was approved by the FDA in 2024 [7]. Resmetirom is an oral, liver‐targeted thyroid hormone receptor‐β (THRβ) selective drug that is effective in the treatment of MASH‐associated liver fibrosis. It can reduce liver fat content, improve liver fibrosis, and alleviate liver injury, but has no significant effect on body weight and glucose metabolism [8]. Considering the high global incidence of MASH, there is an urgent need to discover more safe and effective drugs with novel mechanisms of action to alleviate MASH.

The search for natural compounds with high efficiency and low toxicity to treat complex diseases has become one of the important strategies of drug discovery. *Carpesium abrotanoides* L. is a kind of Chinese medicine widely used to treat hepatitis, bronchitis, and bruises, owing to its diverse pharmacological activities, such as anti‐parasite, insecticidal activity, antidiabetic activity, antioxidant activity, and anti‐inflammatory [9, 10, 11, 12, 13, 14, 15]. Carabrone, one of the main bioactive ingredients extracted from *Carpesium abrotanoides* L., has been emphasized for its biological functions including anti‐inflammatory, anti‐fungal, anti‐virus, and anti‐tumor in recent studies [16, 17]. However, whether carabrone can attenuate MASH is not yet clear.

In this research, we explored the effects of carabrone on MASH pathogenesis, focusing on its influence on hepatic injury, lipid accumulation, inflammation, and fibrosis. Our findings revealed that carabrone markedly reduced hepatic lipid accumulation, inflammation, and fibrosis in multiple mouse models of MASH, including high‐fat/high‐cholesterol (HFHC), high‐fat (HFD), and methionine‐ and choline‐deficient (MCD) diets induced MASH models. Delving into the mechanism, we utilized a biotin‐tagged carabrone chemical probe and validation experiments to pinpoint carabrone's key targets. We discovered that carabrone exerts its anti‐MASH effects by binding directly to the signal transducer and activator of transcription 3 (STAT3) and inhibiting its activation. Furthermore, through structural modifications of carabrone, we identified a derivative with enhanced affinity for STAT3, demonstrating promising therapeutic efficacy against MASH in MCD‐fed mice.

## 2 Results

### 2.1 Carabrone Blocks MASH Progression in HFHC‐Induced Mice

**FIGURE 1:**
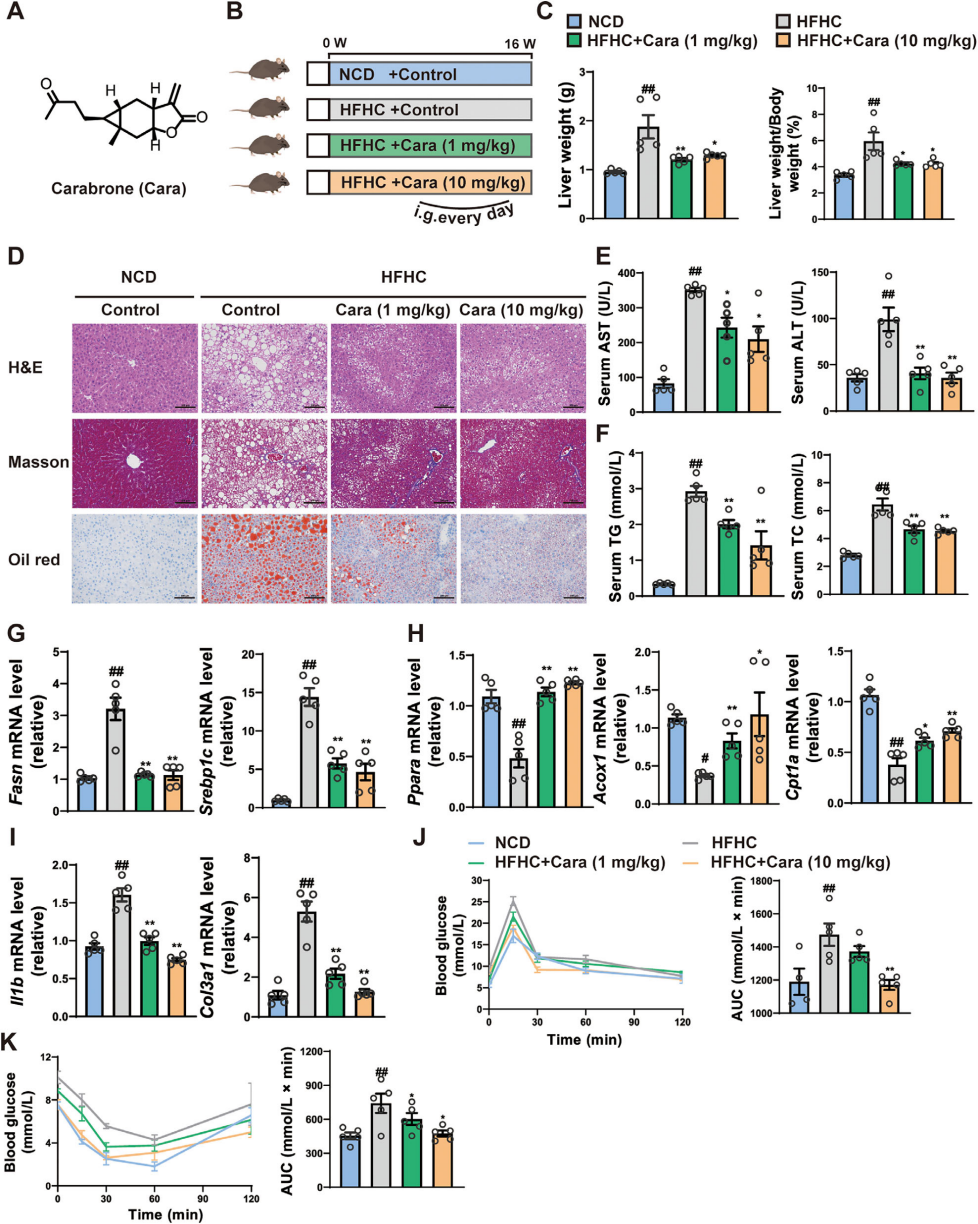
Carabrone blocks MASH progression in HFHC‐induced mice. Eight‐week‐old mice intragastrically administered with 0.5% CMCNa or carabrone (1 or 10 mg/kg) were fed with NCD or HFHC for 16 weeks. *n* = 5 per group. (A) Chemical structure of carabrone. (B) Scheme illustrating carabrone treatment strategy. (C) Liver weight and liver index. (D) Representative images of H&E, Masson, and Oil Red O–stained liver sections. Scale  =  100 µm. (E) Serum ALT and AST levels. (F) Serum TG and TC levels. (G) mRNA levels of genes related to lipogenesis (*Fasn* and *Srebp1c*) and (H) lipoclasis (*Cpt1a*, *Acox1*, and *Ppara*) in the livers. (I) mRNA levels of genes related to inflammation (*Il1b*) and fibrosis (*Col3a1*) in the livers. (J) Oral glucose tolerance test (2.5 g/kg) after feeding for 15 weeks. AUC is indicated on the right. (K) Insulin tolerance test (0.75 U/kg) after feeding for 15 weeks. AUC is indicated on the right. Values represent mean ± SEM. Statistical differences were determined by one‐way ANOVA. ^#^ *p* < 0.05, ^##^ *p* < 0.01 compared with NCD group; *p < 0.05, **p < 0.01 compared with HFHC group.

Wild‐type (WT) mice were subjected to HFHC diet for 16 weeks to induce the MASH phenotype, then the mice were treated with carabrone (intragastrically, 1 and 10 mg/kg/day) or vehicle, and the mice subjected to normal chow diet (NCD) were used as control (Figure 1A,B). After 16 weeks’ treatment, the body weight of HFHC mice increased significantly compared to the NCD mice, while the weight of carabrone‐treated HFHC mice was stable (Figure S1A). Consistent with body weight, the epididymal adipose size of carabrone‐treated mice was significantly lower than that of the HFHC control group (Figure S1B). The liver weights (LWs) and the liver weight‐to‐body weight (LW/BW) of carabrone‐treated mice were also lower than those of the HFHC control group (Figure 1C). In addition, carabrone‐treated mice exhibited lower serum lipid, hepatic lipid accumulation, and de novo lipogenesis but higher β‐oxidation than the HFHC control group (Figure 1D,G,H, Figure S1C). Histological staining displayed that the hepatic inflammatory response and fibrosis deposition were ameliorated in carabrone‐treated mice (Figure 1D). Compared with the HFHC control group, the hepatic profibrotic mediators were markedly reduced in carabrone‐treated group, further demonstrating the inhibitory effect of carabrone on liver fibrosis (Figure 1I), and these effects were accompanied by a reduction in liver injury (Figure 1E). Moreover, carabrone‐treated mice showed much lower blood glucose in OGTT and ITT assays, suggesting that carabrone was able to improve glucose tolerance and insulin tolerance (Figure 1J,K). Taken together, our study suggests that carabrone treatment can alleviate HFHC‐diet‐induced MASH.

### 2.2 Carabrone Blocks MASH Progression in HFD‐Induced Mice

**FIGURE 2:**
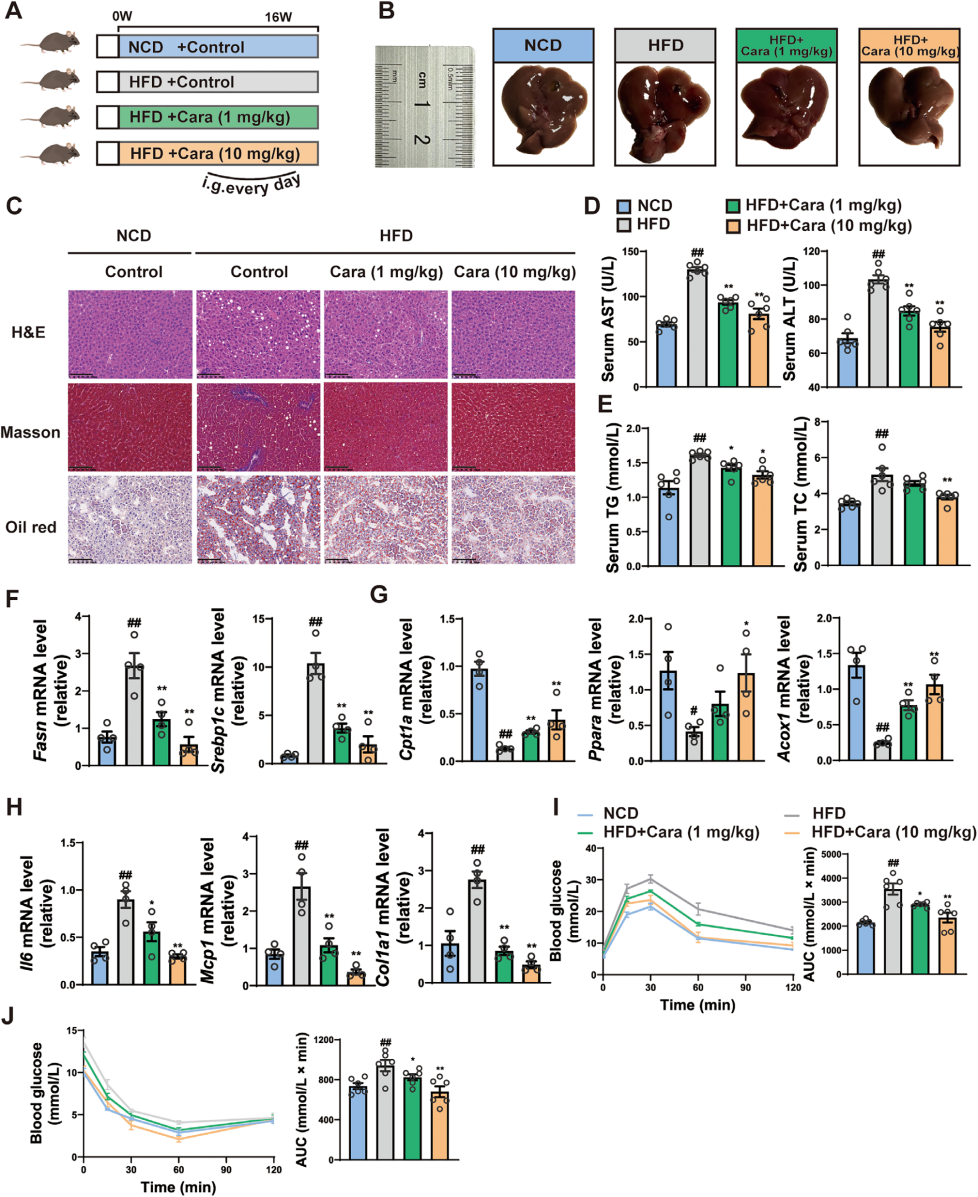
Carabrone blocks MASH progression in HFD‐induced mice. Eight‐week‐old mice intragastrically administered with 0.5% CMCNa or carabrone (1 or 10 mg/kg) were fed with NCD or HFD for 16 weeks. *n* = 6 per group. (A) Scheme illustrating carabrone treatment strategy. (B) Representative image of liver. (C) Representative images of H&E, Masson, and Oil Red O–stained liver sections. Scale  =  100 µm. (D) Serum ALT and AST levels. (E) Serum TG and TC levels. (F) mRNA levels of genes related to lipogenesis (*Fasn* and *Srebp1c*) and (G) lipoclasis (*Cpt1a*, *Acox1* and *Ppara*) in the livers (*n* = 4 per group). (H) mRNA levels of genes related to inflammation (*Il6* and *Mcp1*) and fibrosis (*Col3a1*) in the livers (*n* = 4 per group). (I) Oral glucose tolerance test (2.5 g/kg) after feeding for 15 weeks. AUC is indicated on the right. (J) Insulin tolerance test (0.75 U/kg) after feeding for 15 weeks. AUC is indicated on the right. Values represent mean ± SEM. Statistical differences were determined by one‐way ANOVA. ^#^ *p* < 0.05, ^##^ *p* < 0.01 compared with NCD group; *p < 0.05, **p < 0.01 compared with HFD group.

To further demonstrate the therapeutic effect of carabrone on MASH, we established HFD‐diet‐induced MASH model. After 16 weeks of HFD feeding and oral administration of carabrone (1 and 10 mg/kg/day) (Figure 2A), carabrone‐treated mice showed significantly lower body weight compared to mice fed HFD (Figure S2A,B). Moreover, the contents of liver lipid (TG and TC) and serum lipid (TG and TC) were significantly decreased in carabrone‐treated group (Figure 2D and Figure S2C). H&E and Oil Red O staining revealed that carabrone treatment markedly inhibited the accumulation of lipid droplet in liver tissues (Figure 2C). Serum ALT and AST levels in HFD mice were notably decreased after long‐term administration of carabrone (Figure 2E). Similar to histological results, carabrone‐treated mice exhibited lower mRNA levels of de novo lipogenesis, inflammatory, and fibrosis, but higher β‐oxidation compared with HFD mice (Figure 2F–H). Furthermore, carabrone treatment improved the glucose sensitivity of HFD mice, as shown by the OGTT and ITT results (Figure 2I,J). These findings suggested that carabrone could significantly ameliorate HFD‐diet‐induced MASH.

**FIGURE 3:**
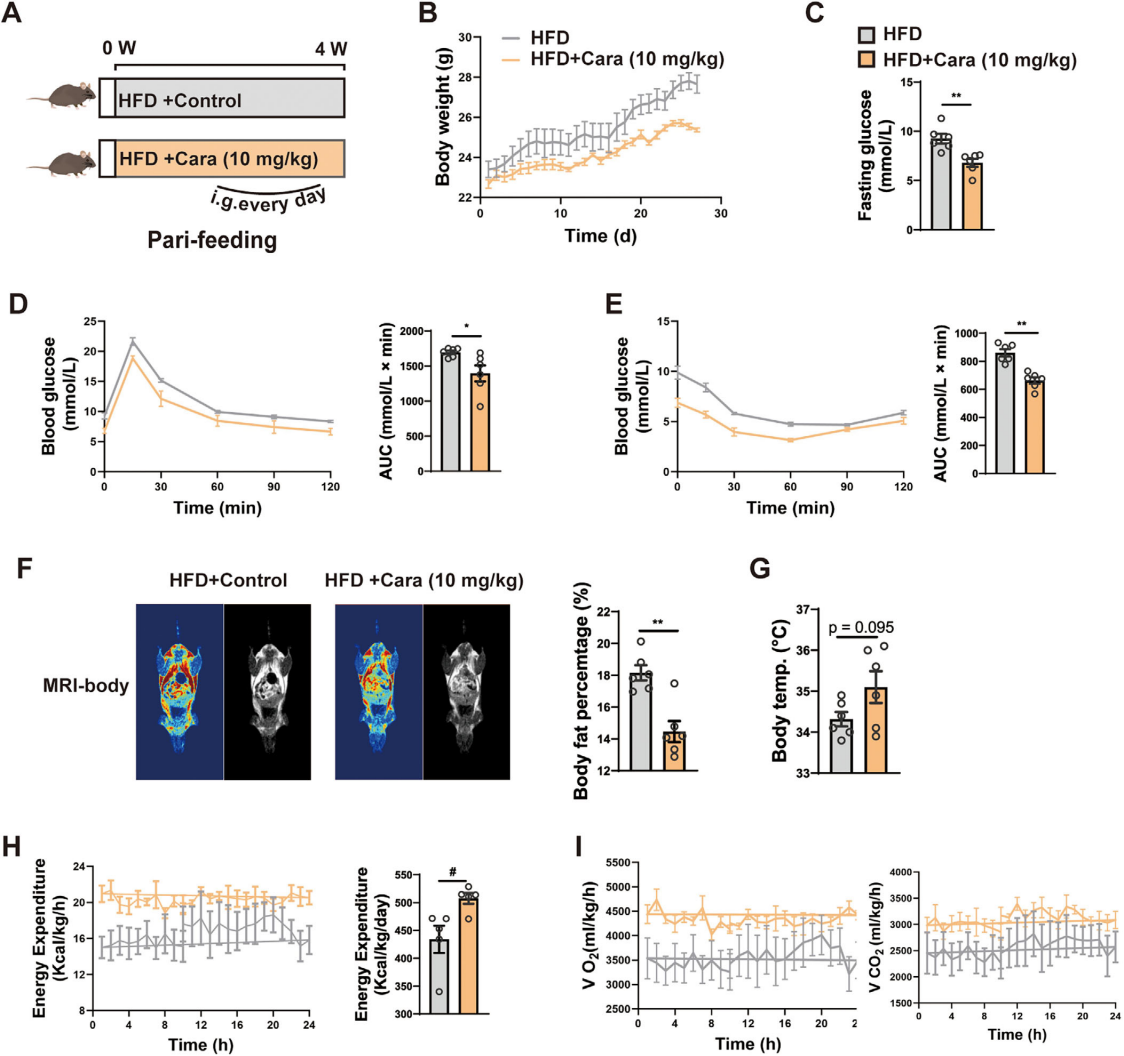
Carabrone improved glucose metabolism and energy expenditure in pair‐fed HFD mice. Eight‐week‐old mice intragastrically administered 0.5% with CMCNa or carabrone (10 mg/kg) were fed with HFD for 4 weeks. *n* = 6 per group. (A) Scheme illustrating carabrone treatment strategy. (B) Body weight. (C) Fasting glucose. (D) Oral glucose tolerance test (2.5 g/kg). AUC is indicated on the right. (E) Insulin tolerance test (0.75 U/kg). AUC is indicated on the right. (F) The representative magnetic resonance imaging (MRI) images of whole‑body fat, and the right panel presented the quantitative data. (G) Body temperature. (H and I) Energy expenditure, CO_2_ production, and oxygen consumption were measured as kilocalories per kilogram lean mass per hour. Values represent mean ± SEM. Statistical differences were determined by one‐way ANOVA. #*p* < 0.05, ##*p* < 0.01 compared with NCD group; *p < 0.05, **p < 0.01 compared with HFD group.

Next, we preformed pair‐feeding experiments in HFD‐fed mice for 4 weeks (Figure 3A) and observed a similar increase in body weight (Figure 3B). Carabrone‐treated mice showed much lower blood glucose in OGTT and ITT assays (Figure 3C–E). MRI analyses demonstrated that carabrone treatment reduced the whole‐body and liver fat content, which was supported by the decrease of body weight (Figure 3F). The body temperature and energy expenditure of carborne‐treated mice elevated (Figure 3G,H), as did the respiratory exchange ratio (Figure 3I), which may contribute to body weight loss. The reduction of obesity and hepatic steatosis may lead to an improved glucose metabolic phenotype. We administered an HFD to mice for 5 days to investigate the influence of lipid metabolism on glucose phenotypes (Figure S3A). Our findings revealed that while HFD impacted glucose phenotypes, it did not significantly affect lipid metabolism as evidenced by unchanged body weight, epididymal fat mass, and hepatic lipid droplet accumulation (Figure S3B–D). Furthermore, we observed that the carabrone attenuated HFD‐induced hyperglycemic events (Figure S3E,F). These results indicated that the carabrone could ameliorate glucose disorders independently of lipid metabolic improvements.

### 2.3 Carabrone Attenuates Lipid Accumulation and Inflammation in Hepatocytes Stimulated by Palmitic Acid and Oleic Acid

**FIGURE 4:**
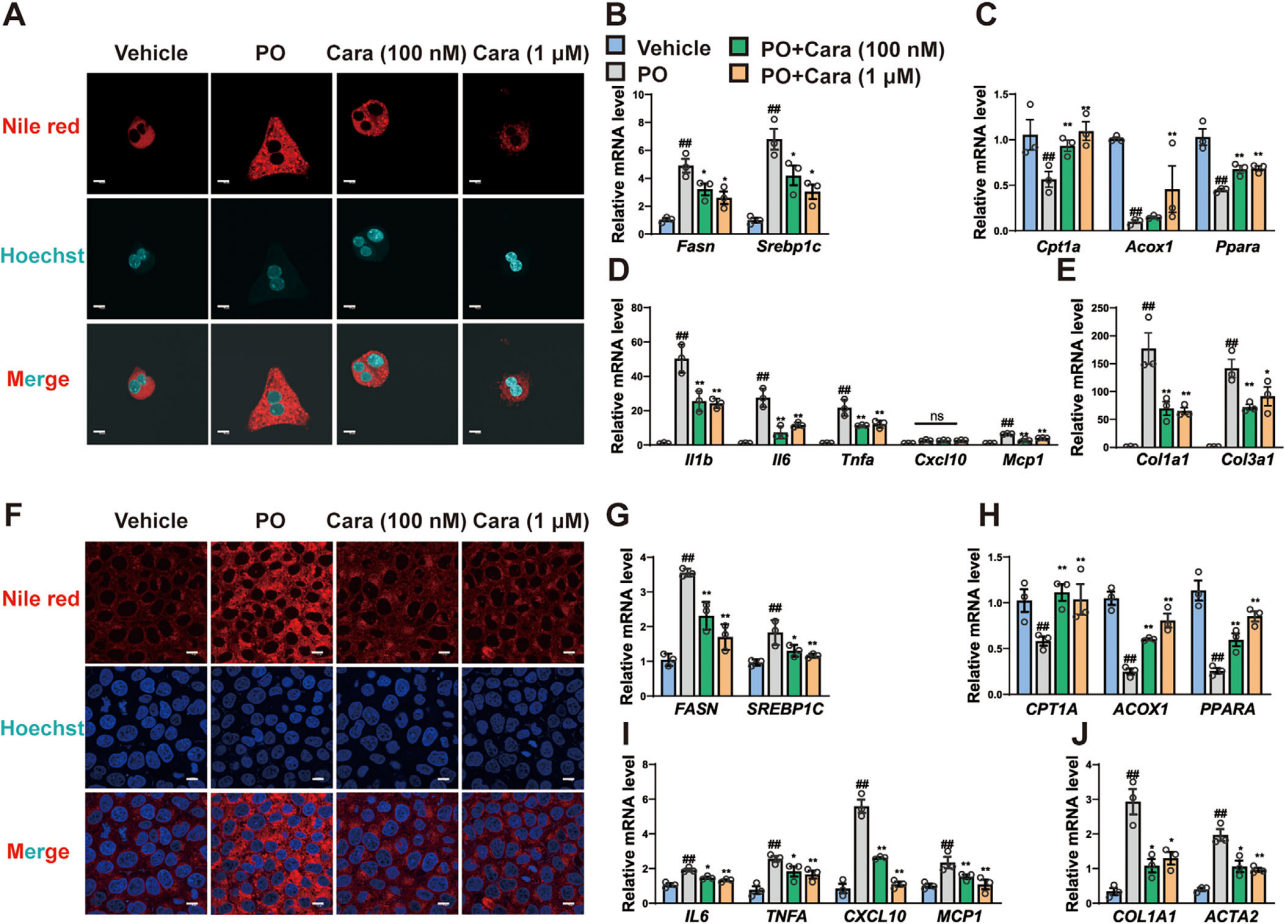
Carabrone attenuates PO‐stimulated lipid accumulation and inflammation in hepatocytes. Primary hepatocytes and L02 cells were cultured in PO medium containing 0.4 mM PA and 0.4 mM OA and treated with carabrone (100 nM and 1 µM) for 24 h. (A) Representative Nile Red staining images of primary hepatocytes. Scale  =  10 µm. (B) mRNA levels of lipogenesis (*Fasn* and *Srebp1c*) and (C) lipoclasis (*Cpt1a*, *Acox1*, and *Ppara*) related genes in primary hepatocytes. *n* = 3 per group. (D) mRNA levels of inflammation (*Il1b*, *Il6*, *Tnfa*, *Cxcl10*, and *Mcp1*) and (E) fibrosis (*Col1a1* and *Col3a1*) related genes in primary hepatocytes. *n* = 3 per group. (F) Representative Nile Red staining images of L02 cells. Scale  =  10 µm. (G) mRNA levels of lipogenesis (*FASN* and *SREBP1C*) and (H) lipoclasis (*CPT1A*, *ACOX1*, and *PPARA*) related genes in L02 cells. *n* = 3 per group. (I) mRNA levels of inflammation (*IL6*, *TNFΑ*, *CXCL10*, and *MCP1*) and (J) fibrosis (*COL1A1* and *ACTA2*) related genes in L02 cells. *n* = 3 per group. Values represent mean ± SEM. Statistical differences were determined by one‐way ANOVA. ^#^ *p* < 0.05, ^##^ *p* < 0.01 compared with control group; *p < 0.05, **p < 0.01 compared with PO group.

Primary hepatocytes are the main cell type of the liver tissue and are closely related to the occurrence of liver metabolic disorders. Thus, the effects of carabrone (0.1 and 1 µM) on lipid accumulation, inflammation, and fibrosis of primary hepatocytes and L02 cells stimulated by palmitic acid and oleic acid (PO) were investigated. Oil Red O and Nile Red staining showed that the lipid accumulation in PO‐treated primary hepatocytes and L02 cells were significantly increased, while the lipid accumulation in carabrone‐treated group was significantly decreased (Figure 4A,F, Figure S4A,B). Carabrone treatment also reduced the expressions of the lipogenic genes *Fasn* and *Srebp1c*, and increased the expressions of lipoclastic genes *Cpt1a*, *Acox*1 and *Ppara* in PO‐treated primary hepatocytes (Figure 4B,C). Furthermore, the expressions of proinflammatory (*Il1b*, *Il6*, *Tnfα*, *Cxcl10*, and *Mcp1*) and fibrotic genes (*Col1a1* and *Col3a1*) in PO‐treated primary hepatocytes were significantly decreased after carabrone treatment (Figure 4D,E). Similar results were also observed in quantitative PCR assay of PO‐treated L02 cells after carabrone treatment (Figure 4G–J). Collectively, above results suggest that carabrone can attenuate lipid accumulation and inflammation in PO‐treated hepatocytes.

### 2.4 STAT3 is a Direct Target of Carabrone in Hepatocytes

**FIGURE 5:**
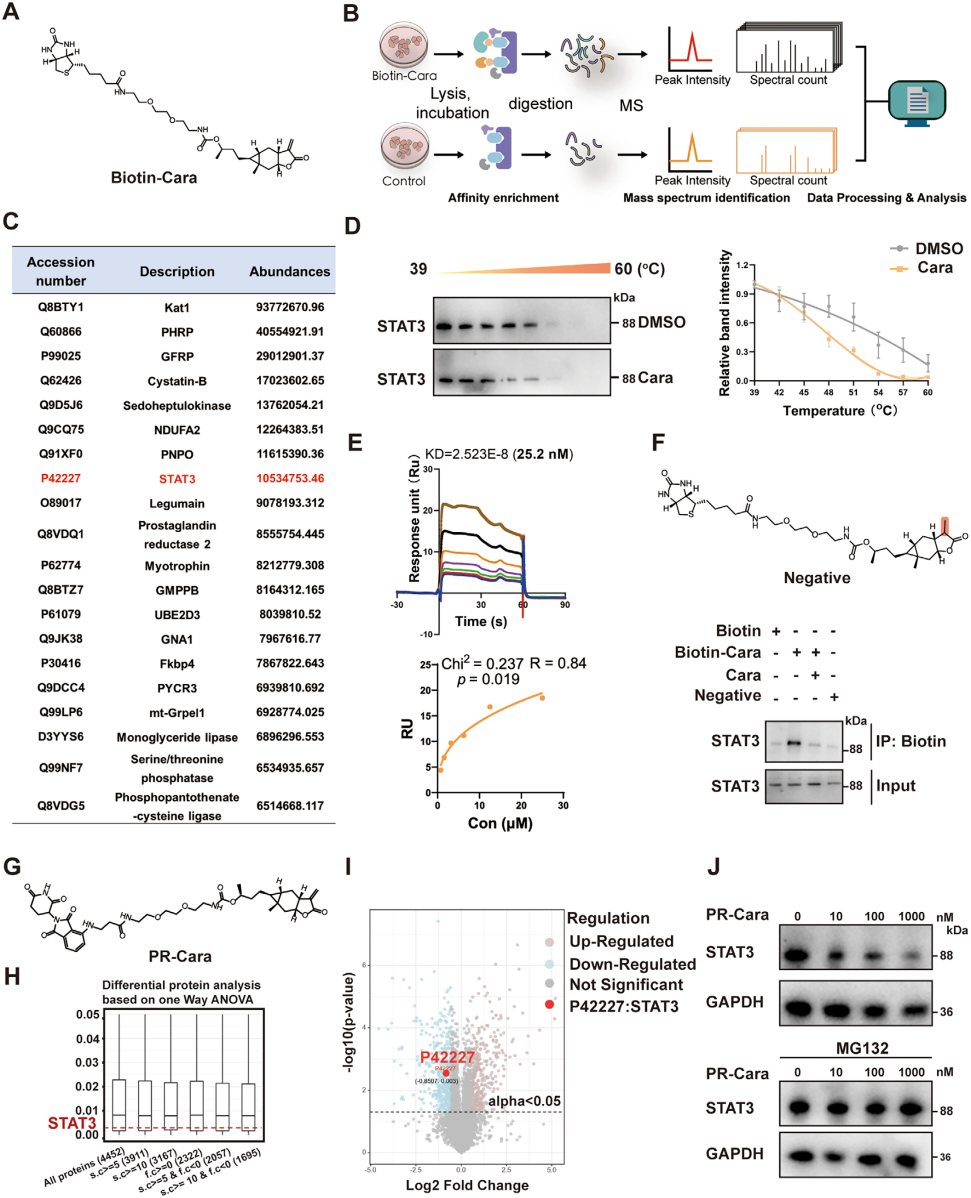
STAT3 is a direct target of carabrone in hepatocytes. (A) The chemical structure of Biotin–Cara. (B) Procedure of target identification by immunoprecipitation‐mass spectrometry. (C) The top 20 abundant proteins binding to carabrone. (D) Cellular thermal shift assay (CETSA) for in‐cell STAT3 target engagement. Representative western blots showed thermostable STAT3 with indicated heat shocks (*n* = 3). (E) The interaction between carabrone and STAT3 confirmed by surface plasmon resonance. The surface of STAT3 biosensor was injected with different concentrations of carabrone. (F) Immunoprecipitation analysis of the interactions between biotin–Cara, biotin or negative‐probe, and STAT3 in the absence or presence of excess unlabeled carabrone. (G) The chemical structure of PROTAC probe (PR‐Cara). (H) Boxplot depicting the distribution of *p*‐values across six groups in a differential protein analysis in PO‐stimulated primary hepatocytes treated with PROTAC probe. Groups include proteins with sequence coverage ≥ 5, sequence coverage ≥ 10, fold change < 0 (downregulated), fold change < 0 and sequence coverage ≥ 5, fold change < 0 and sequence coverage ≥ 10. The line indicates the *p*‐value of protein P42227, highlighting its statistical significance. s.c., sequence coverage; f.c., fold change. (I) Boxplot depicting the distribution of *p*‐values across six groups in a differential protein analysis in PO‐stimulated primary hepatocytes treated with PROTAC probe. Groups include proteins with sequence coverage ≥ 5, sequence coverage ≥ 10, fold change < 0 (downregulated), fold change < 0 and sequence coverage ≥ 5, fold change < 0 and sequence coverage ≥ 10. The line indicates the *p*‐value of protein P42227, highlighting its statistical significance. s.c., sequence coverage; f.c., fold change. (J) Protein expression of STAT3 in primary hepatocytes incubated with PR‐Cara with or without MG132.

To identify the functional targets of carabrone that are responsible for its potent anti‐MASH effect, several chemical probes for affinity purification were prepared. The optimal chemical probe biotin‐tagged carabrone (Biotin–Cara) was determined by in vitro cell activity screening (Figure S5A,B). Next, we performed a pulldown assay by incubating the hepatocytes lysates with biotin–Cara or free biotin. The proteins bind to carabrone were precipitated by streptavidin‐agarose beads, followed by SDS‐PAGE and mass spectrometry analysis (Figure 5A,B). Proteome Discoverer 2.4 was used to calculate the averaged SILAC ratio for all controlled and competing experiments, and 226 proteins were collectively identified as potential targets bound to carabrone (Table S5). Among the top 10 interacting proteins in protein abundances, STAT3 plays a key role in the pathogenesis of liver diseases (Figure 5C). Moreover, KEGG analysis of the pathways associated with these 226 proteins disclosed a top‐ranking functional cluster of “STAT3‐related” (Figure S5C). Given the critical role of STAT3 in inflammation progression, we hypothesized that STAT3 might be the direct pharmacological target of carabrone in hepatocytes.

To test this hypothesis, we employed biophysical analysis of the interaction between STAT3 and carabrone. Cellular thermal shift assay (CETSA) showed that carabrone decreased the thermal stability of STAT3 protein (Figure 5D). Surface plasmon resonance (SPR) analysis demonstrated that carabrone had strong binding affinity to STAT3 (KD = 25.23 nM, Figure 5E). Biotin–Cara probe effectively pulled STAT3 protein out, while the addition of unlabeled carabrone reduced their binding due to competitive binding (Figure 5F). The negative probe synthesized by reducing the carbon–carbon double bond of the Michael receptor could not pull STAT3 protein out (Figure 5F).

Recently, proteolysis targeting chimera (PROTAC) has emerged as a new approach to selectively degrade target proteins using endogenous proteasome, providing a new strategy for the identification of natural product targets [18]. In this work, we further designed a carabrone‐based PROTAC probe PR‐Cara to identify its potential targets for the treatment of MASH (Figure 5G). The PO‐stimulated hepatocytes were treated with PR‐Cara, followed by mass spectrometry analysis. A one‐way ANOVA with *p* value < 0.05 and a fold change > 0.5 were employed to identify proteins exhibiting significantly different expression under experimental conditions. A total of 334 upregulated proteins and 395 downregulated proteins were identified (Table S6). Specifically, we investigated proteins categorized by sequence coverage thresholds and fold change values indicative of down‐regulation. Notably, STAT3 protein emerged as statistically significant, demonstrating its importance (Figure 5H). The ggplot2 package (R software) was used to map the volcano plot of the distribution of those identified proteins, with STAT3 highlighted by the red point (Figure 5I). Western blot showed that PROTAC probe treatment decreased the expression of STAT3, but the addition of proteasome inhibitor MG132 blocked this effect (Figure 5J). Together, these data revealed the strong interaction between carabrone and STAT3.

### 2.5 Direct Inactivation of STAT3 by Carabrone

**FIGURE 6:**
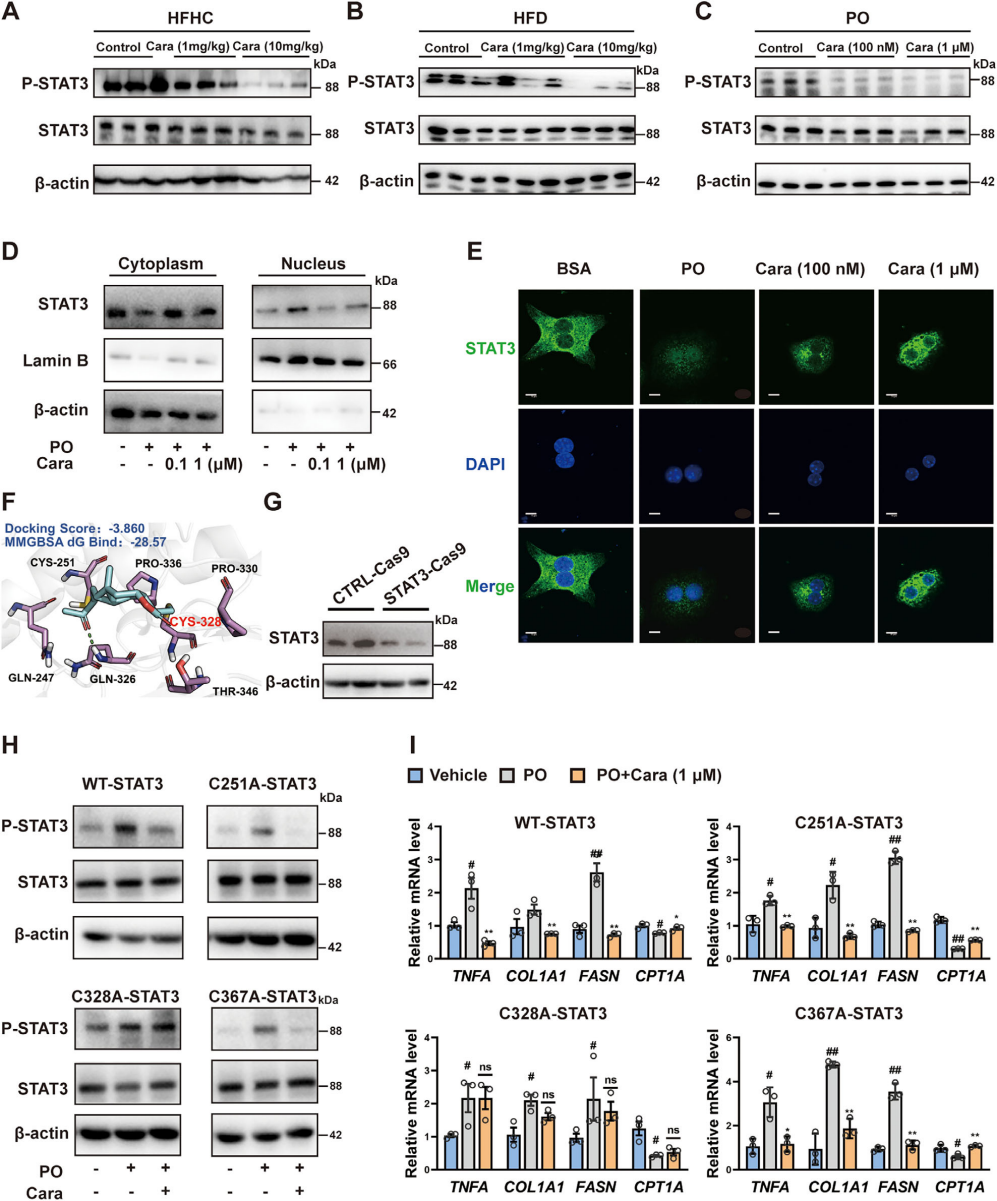
Direct inactivation of STAT3 by carabrone. (A) Protein expression of STAT3 and p‐STAT3 in the livers of HFHC or (B) HFD‐fed mice. *n* = 3 per group. (C) Protein expression of STAT3 and p‐STAT3 in PO‐stimulated primary hepatocytes. *n* = 3 per group. (D) Protein expression of STAT3 and p‐STAT3 in the cytoplasm and nuclear of PO‐stimulated primary hepatocytes with or without carabrone treatment (1 µM, 24 h). (E) Representative confocal images of STAT3 nuclear translocation in PO‐stimulated primary hepatocytes with or without carabrone treatment (1 µM, 24 h). Scale  =  10 µm. (F) Binding modes and sites of carabrone with STAT3 predicted by molecular docking. (G) Protein expression of STAT3 in STAT3‐KO L02 cells by CRISPR/Cas9 method. (H) Protein expression of STAT3 and p‐STAT3 in STAT3‐KO/WT L02 cells transfected with plasmids encoding the WT, C251A, C328A, C367A mutation of STAT3, with or without carabrone treatment (1 µM, 24 h). (I) mRNA levels of *TNFA*, *COL1A1*, *FASN*, and *CPT1A* in STAT3‐KO/WT L02 cells transfected with plasmids encoding the WT, C251A, C328A, C367A mutation of STAT3, with or without carabrone treatment (1 µM, 24 h). *n* = 3 per group. Values represent mean ± SEM. Statistical differences were determined by one‐way ANOVA. ^#^ *p* < 0.05, ^##^ *p* < 0.01 compared with control group; *p < 0.05, **p < 0.01 compared with PO group.

To corroborate the role of STAT3 activation in carabrone‐mediated anti‐MASH effect, STAT3 was overexpressed in hepatocytes using plasmid interference (Figure S6A). It was observed that the effects of carabrone on attenuating PO‐stimulated lipid accumulation and inflammation were completely lost in this case (Figures S6B–D). Since the tyrosine phosphorylation results in STAT3 dimerization and translocation into the nucleus, thereby regulating MASH‐related genes expression [19], we further assessed the effect of carabrone on these processes. The results in Figure 6A–C showed that carabrone treatment reduced the STAT3 phosphorylation in the livers of HFHC‐fed mice, HFD‐fed mice, and PO‐stimulated hepatocytes, as well as decreased the translocation of STAT3 into nucleus in PO‐stimulated hepatocytes (Figure 6D). Immunofluorescence staining results further confirmed the roles of carabrone in STAT3 nuclear translocation (Figure 6F).

Subsequently, the binding modes of carabrone to STAT3 were simulated by the covalent docking module of Schrödinger. According to the docking score and binding free energy (MM‐GBSA), the top three residues were Cys251, Cys328, and Cys367 (Figure 6F and Figure S6E,F). To identify which cysteine residue was most critical for the inhibitory activity of carabrone on STAT3 activation, these cysteines were mutated to alanine residues, respectively, and the L02 cells with STAT3 knockdown were constructed using CRISPR/Cas9 method (STAT3‐Cas9 L02 cells, Figure 6G). It was found that carabrone remarkably inhibited the phosphorylation of the transfected wild type (WT), C251A mutant and C367A mutant STAT3, except for C328A mutant STAT3 in PO‐stimulated Cas9‐L02 cells (Figure 6H), while mutation with alanine residue did not alter the effect of STAT3 on PO‐stimulated hepatocytes (Figure S6G). Furthermore, compared with wild‐type STAT3, the effect of carabrone on inhibiting *TNFA*, *FASN*, *CPT1A*, and *COL1A1* expressions was diminished by C328A mutation, but not by C251A and C367A mutations (Figure 6I). Similar results were observed in STAT3‐knockdown primary hepatocytes using siRNA transfection method (Figure S6H). These data indicated that STAT3 was a key target for carabrone to exert anti‐MASH effect, and Cys328 was critical for carabrone‐mediated inhibition on STAT3 activation.

### 2.6 A New Carabrone Derivative Was Found to Have Stronger Anti‐MASH Activity and Affinity for STAT3

**FIGURE 7:**
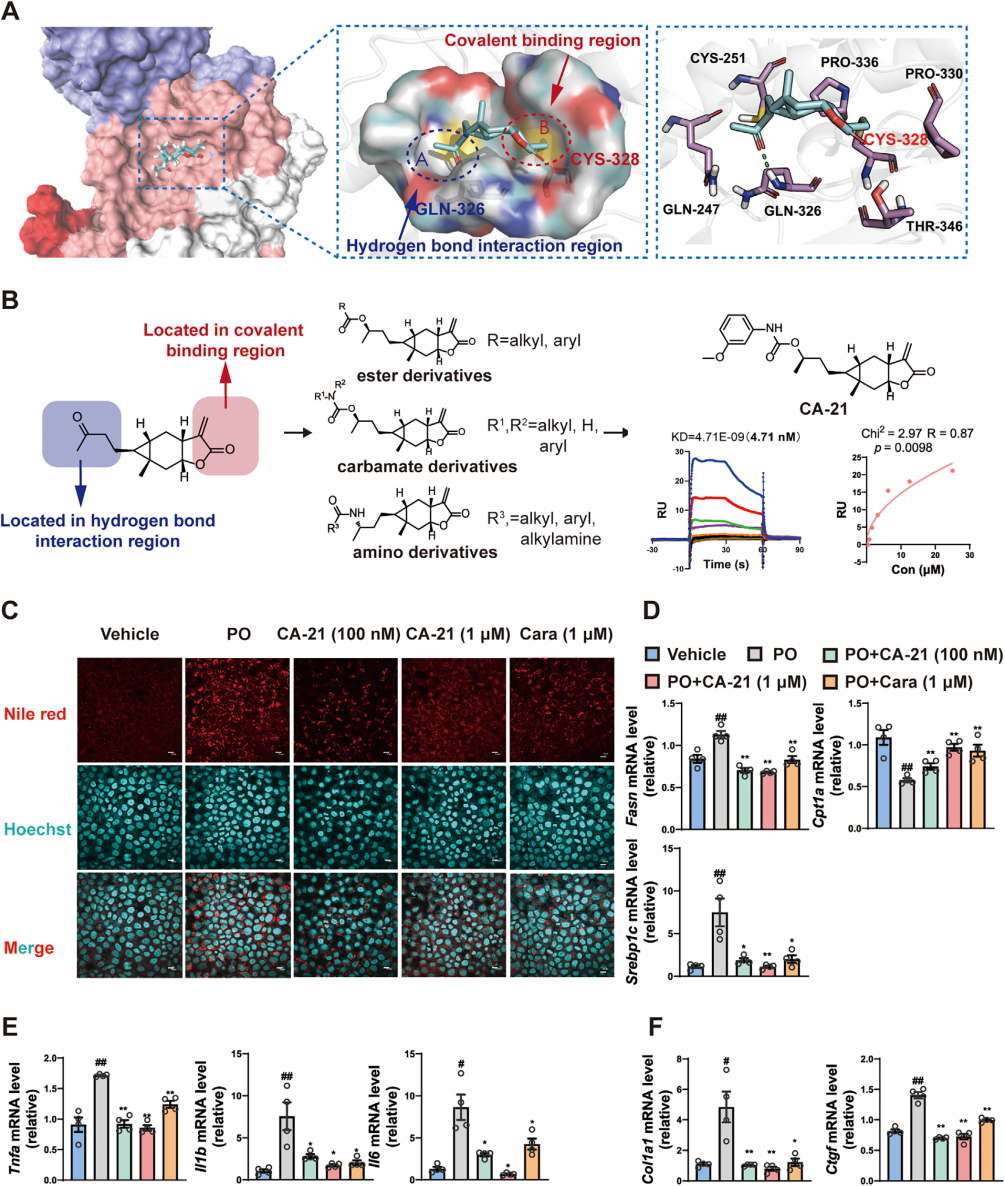
A new carabrone derivative was found to have stronger anti‐MASH activity and affinity for STAT3. (A) Analysis of the covalent docking mode between carabrone and STAT3. (B) The discovery of carabrone derivative **CA‐21**. (C) Representative Nile Red staining images of PO‐stimulated primary hepatocytes treated with **CA‐21** (100 nM and 1 µM) for 24 h. Scale = 10 µm. (D–F) mRNA levels of *Fasn*, *Srebp1c*, *Cpt1a*, *Tnfa*, *Il1b*, *Il6*, *Col1a1*, and *Ctgf* in PO‐stimulated primary hepatocytes treated with **CA‐21** (100 nM and 1 µM) or carabrone (1 µM) for 24 h. *n* = 4 per group. Values represent mean ± SEM. Statistical differences were determined by one‐way ANOVA. ^#^ *p* < 0.05, ^##^ *p* < 0.01 compared with control group; *p < 0.05, **p < 0.01 compared with PO group.

To avoid the potential side effects from off‐target effects, structure modification of carabrone was carried out to improve its affinity to STAT3 (Figure S11‐23). To make a reasonable modification of carabrone, we first analyzed the binding mode of carabrone to STAT3 by molecular docking. The covalent docking result indicated that the unsaturated lactone ring of carabrone was located in the key covalent binding region and was not suitable for structural modification. In contrast, the terminal carbonyl group of carabrone was positioned in the hydrogen bond interaction region, making it a suitable site for modification to enhance the interaction with STAT3 (Figure 7A). Encouraged by above analysis result, three series of carabrone derivatives were designed, synthesized, and evaluated for their anti‐MASH activity by determining the mRNA levels of proinflammatory genes (*Il1b*, *Il6*) in PO‐treated primary hepatocytes (Tables S2–S4), and compounds with stronger anti‐MASH activities than carabrone were selected to evaluate their binding affinity to STAT3 by SPR (see Supporting Information for detail, Figure S7). The results showed that carabrone derivative **CA‐21** (Figure 7B) had the strongest anti‐MASH activity in vitro, as well as robust binding affinity to STAT3 (Kd = 4.63 nM), which was much improved compared to carabrone (Kd = 25.23 nM). Then, the effects of **CA‐21** on lipid accumulation, inflammation, and fibrosis in PO‐treated primary hepatocytes and L02 cells were investigated. The results showed that **CA‐21** treatment significantly improved lipid accumulation in PO‐treated L02 cells (Figure 7C) and reduced the expressions of lipogenic genes (*Fasn* and *Srebp1c*) and increased the expression of lipoclastic gene *Cpt1a* in PO‐treated primary hepatocytes (Figure 7D). Moreover, the expressions of proinflammatory (*Il1b*, *Il6*, and *Tnfa*) and fibrotic genes (*Col1a1* and *Ctgf*) were significantly decreased after **CA‐21** treatment in PO‐treated primary hepatocytes (Figure 7E,F). Compared to carabrone, **CA‐21** not only exhibited stronger anti‐MASH effects in vitro (Figure 7D,F), but also showed higher safety in hepatocyte (Figure S8).

### 2.7 Carabrone Attenuates MASH in a STAT3‐Dependent Manner in MCD‐Fed Mice

**FIGURE 8:**
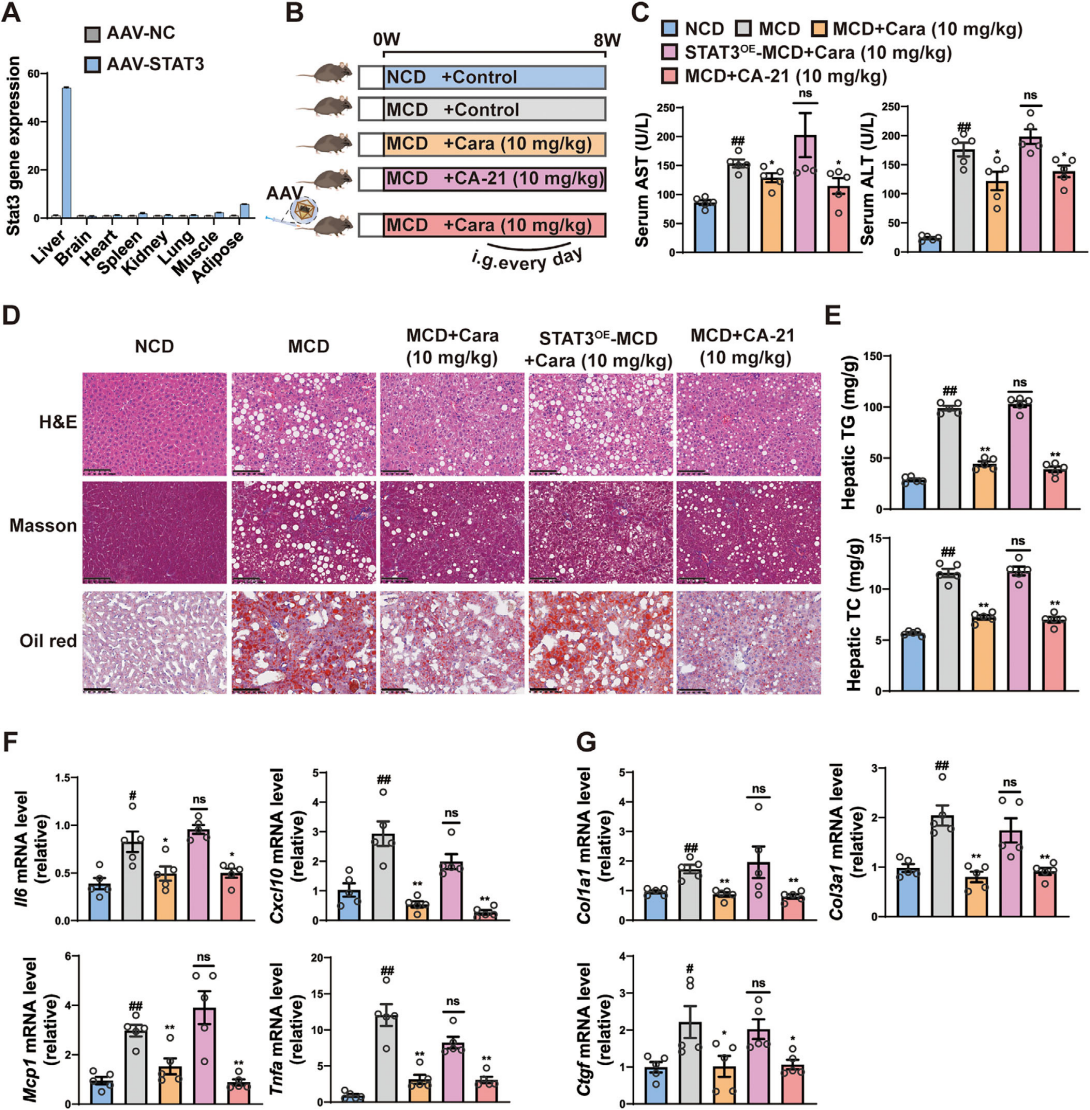
Carabrone attenuates MASH in a STAT3‐dependent manner in MCD‐fed mice. Eight‐week‐old mice injected with AAV8‐NC or AAV8‐STAT3 were fed with NCD or MCD for 8 weeks, intragastrically administered with 0.5% CMCNa, carabrone or **CA‐21** (10 mg/kg). *n* = 5 per group. (A) mRNA levels of STAT3 in the livers of mice injected with AAV8‐NC or AAV8‐STAT3. (B) Schematic diagram of the experimental procedure. (C) Serum ALT and AST levels. (D) Representative images of H&E, Masson, and Oil Red O–stained liver sections. Scale  =  100 µm. (E) TG and TC levels in the livers. (F) mRNA levels of inflammation (*Il6*, *Cxcl10*, *Mcp1*, and *Tnfa*) and (G) fibrosis (*Col1a1*, *Col3a1*, and *Ctgf*) related genes in the livers. Values represent mean ± SEM. Statistical differences were determined by one‐way ANOVA. ^#^ *p* < 0.05, ^##^ *p* < 0.01 compared with NCD group; *p < 0.05, **p < 0.01 compared with MCD group.

Finally, to evaluate whether carabrone alleviates MASH through the STAT3 pathway, mouse model with hepatic STAT3 overexpression was established by injecting AAV8 plasmid via tail vein (Figure 8A). Then the mice were subjected to MCD diet and treated with carabrone or **CA‐21** for 8 weeks (Figure 8B). Consistent with the results observed in HFHC and HFD mouse model, the serum ALT and AST levels, and liver TG and TC contents were significantly reduced after carabrone treatment (Figure 8C,E). Histological staining showed less steatosis and inflammatory cell infiltration in carabrone‐treated mice compared to control MCD mice (Figure 7D). Quantitative PCR assays (Figure 8F,G) also revealed that carabrone treatment decreased the expressions of inflammation (*Tnfα*, *Mcp1*, and *Cxcl10*) and fibrosis‐related genes (*Col1a1*, *Col3a1*, and *Ctgf*). However, these improvements were not observed in STAT3‐overexpressed mice (Figure 8C,G). Collectively, these results suggest that carabrone's therapeutic effect on MASH is dependent on the STAT3 pathway. More importantly, we obtained a new candidate **CA‐21**, which exhibits better ameliorating effect on MCD diet‐induced MASH compared to carabrone (Figure 8).

## 3 Discussion

Due to the escalating incidence and grave ramifications of MASH, patients afflicted with this condition have become a primary focus for liver transplantation, underscoring the urgent imperative for effective treatments [20]. While exercise training and dietary interventions remain the basic recommendations for MAFLD patients, their efficacy wanes for those advancing to the MASH stage [21]. Thus, pharmacological interventions for MAFLD and MASH are essential. Natural products, particularly the active compounds extracted from traditional Chinese medicine from various sources, have been widely used in the treatment of inflammatory diseases [22]. In this work, we show for the first time that carabrone effectively prevent the progression of MASH in HFHC‐, HFD‐, and MCD‐diet‐induced mice by regulating dysregulation of glucose metabolism, lipid accumulation, inflammation, and fibrosis. Additionally, carabrone mitigated lipid accumulation and inflammation induced by PO stimulation in primary hepatocytes and L02 cells. Mechanistically, a quantitative chemical proteomics study was conducted to identify the target proteins of carabrone, revealing direct binding and inactivation of STAT3 by carabrone. Moreover, carabrone‐mediated ameliorating effects on lipid accumulation and inflammation in PO‐treated hepatocytes and MCD mice livers were significantly diminished after STAT3 overexpression. Our findings suggest that carabrone ameliorates MASH by suppressing STAT3 activation and thereby regulating fatty acid metabolism and inflammation, providing a promising therapeutic strategy for MASH treatment (see graphical abstract).

An ABPP‐based chemical proteomic strategy was employed to identify the target proteins of carabrone. Our investigations unveiled 226 proteins that may be influenced by carabrone binding. The top 10 interacting proteins were ACAT3, GSTM6, KYAT1, PTER, GCHFR, CSTB, SHPK, STAT3, NDUFA2, and PNPO, among which STAT3 plays a crucial role in the pathogenesis of liver diseases and is therefore identified as a prime target of carabrone [23]. It has been reported that the PKA‐STAT3 signaling pathway in the liver of wild mice induces M2‐like activation of primary Kupfer cells [24]. Additionally, the combination of blueberries and probiotics has been found to be effective in alleviating MASH, partly by inhibiting IL‐22‐mediated JAK1/STAT3/BAX signaling pathway [25, 26]. Kahweol's anti‐inflammatory effect on liver inflammation was found to be associated with reduced expression of phospho‐NF‐kB and phospho‐STAT3. Moreover, treatment of HCC with small molecule STAT3 inhibitor C188‐9 conferred resistance to MASH‐associated liver fibrosis [27]. Given the critical role of STAT3 in hepatic inflammation progression, we selected STAT3 for subsequent validations. SPR analysis demonstrated direct binding of carabrone to recombinant STAT3 protein, a finding corroborated by CETSA and pull‐down assays. Taken together, our findings suggest that carabrone exerts its anti‐MASH effects by interaction with STAT3, at least in the case of PO‐stimulated hepatocytes and diet‐induced MASH.

An interesting question arose in our study was whether dysregulation of glucose metabolism, lipid accumulation, inflammation, and fibrosis caused by STAT3 activation were limited to the MASH stage, since STAT3 plays different roles in different liver diseases or stages. Particularly, evidence confirms that STAT3 is a critical anti‐inflammatory signal in MAFLD, regulating MAFLD‐induced liver inflammation and fibrosis. Zhao et al. reported that liver metabolic regulator STC2 improved hypertriglyceridemia and hepatosteatosis in HFD‐induced MAFLD mice by activating the STAT3 signaling pathway [23]. In ob/ob and high‐fat diet‐induced obese mice, IL‐6 ameliorated fatty liver by inducing STAT3 phosphorylation, while in IL‐10 knockout mice, deficiency of IL‐6 or STAT3 led to steatosis and hepatocellular injury [28, 29]. It has been reported that IL‐22 promotes the progression of MAFLD by activating STAT3 in hepatocytes [30, 31], MAPK and JAK/STAT3 signaling pathways are necessary for IL‐6 activation in MAFLD [32, 33], and STAT3 confers resistance to hepatocellular damage, acting as a cell survival signal in numerous rodent liver injury models [34, 35, 36]. Furthermore, Wan et al. found that betulinic acid or betulin alleviated acute ethanol‐induced fatty liver by downregulating the expression of Toll‐like receptor 4 and promoting the phosphorylation STAT3 in mice and HSCs, providing a potential treatment for ethanol‐induced fatty liver [31]. These findings imply that STAT3 plays distinct roles in different stages of liver disease. In the MAFLD stage, moderate activation of STAT3 can be hepatoprotective, whereas in the MASH stage, excessive activation of STAT3 may exert pro‐inflammatory effects.

To enhance compound's binding affinity to STAT3 protein and its anti‐MASH efficacy, as well as to discover new candidate compounds, three series of derivatives were synthesized through the structural modifications of carabrone. In vitro activity screening revealed that derivative **CA‐21** exhibited stronger anti‐MASH activity and affinity for STAT3 compared to carabrone. In MCD‐diet‐induced MASH mice, **CA‐21** also demonstrated better efficacy in mitigating liver lipid accumulation, inflammation, and fibrosis. These findings suggested that **CA‐21** hold promise as a more viable candidate for MASH treatment.

Despite the encouraging findings, our work still has some limitations: (1) Whether other top‐ranking proteins identified play a role in MASH remains to be further validation. (2) The Cre/loxP‐dependent conditional gene‐targeting approach should be used to confirm that carabrone exerts anti‐MASH activity through interaction with STAT3. (3) The specific binding mode of carabrone and STAT3 needs to be confirmed by analyzing the complex crystal structure of STAT3 and carabrone, but it remains a challenge.

In summary, this work reported for the first time that carabrone ameliorated MASH by binding STAT3 and inhibiting its activation. More importantly, we discovered a new carabrone derivative **CA‐21** with stronger anti‐MASH activity and binding affinity for STAT3, providing new insights into the development of MASH therapeutics.

## 4 Materials and Methods

### 4.1 Animals and Treatments

All animal procedures were ethically approved by the Animal Ethics Committee of Nanjing University of Chinese Medicine. Male C57BL/6J mice aged 6–8 weeks were procured from Jiangsu Qinglongshan Biotech Co. Ltd. (China). Following a week‐long acclimation period, the mice were raised in a controlled environment with a 12‐h light–dark cycle, receiving ad libitum access to food and water. The mouse models of MASH were established by feeding the mice an HFHC (Research Diet, USA) for 16 weeks, HFD (D12492, Research Diet) for 16 weeks, or MCD (A02082002BR, Research Diet) for 8 weeks. Carabrone was administered orally at doses of 1 or 10 mg/kg on a daily basis.

### 4.2 Oral Glucose and Insulin Tolerance Test in Mice

For oral glucose tolerance test, mice were fasted overnight and then orally administered with glucose (2.5 g/kg) via gavage. In the insulin tolerance test, fasted mice received insulin (0.75 U/kg) via intraperitoneal injection. Glucose levels were monitored at 0, 15, 30, 60, 90, and 120 min post‐challenge using ONETOUCH Ultra blood glucose meters (Johnson, USA).

### 4.3 Serum Biochemistry Analysis

Serum levels of total cholesterol (TC), triglycerides (TG), alanine aminotransferase (ALT), and aspartate aminotransferase (AST) were determined using commercial assay kits according to manufacturer's instructions: Total Cholesterol Assay Kit (A111‐1‐1, Njjcbio), Triglyceride Assay Kit (C110‐1‐1, Njjcbio), Alanine Aminotransferase Assay Kit (C009‐2‐1, Njjcbio), and Aspartate Aminotransferase Assay Kit (C010‐2‐1, Njjcbio).

### 4.4 Histology

Liver tissues were stained using standard protocols with hematoxylin and eosin (H&E) for general tissue morphology and Masson's trichrome for collagen fiber visualization. Hematoxylin stained the nuclei (blue) while eosin stained the cytoplasm (red). Oil Red O staining was performed on fresh liver lobes embedded in OCT, rapidly frozen in liquid nitrogen.

### 4.5 Quantitative Real‐Time Reverse Transcription PCR (qRT‐PCR)

Total mRNA was extracted using TRIzol Reagent (RNA isolator Total RNA Extraction reagent, R401‐01, Vazyme, China) following the manufacturer's instructions. cDNA synthesis was carried out using a cDNA Synthesis kit (HiScript II Q RT SuperMix for Qpcr, R223‐01, Vazyme). qRT‐PCR was conducted on the Roche LightCycler 96 System utilizing Fast SYBR Green Master Mix (AceQ qPCR SYBR GreenMaster Mix, R121‐02, Vazyme). Primer sequences are detailed in Table S1.

### 4.6 Cell Culture

Primary hepatocytes were obtained from the livers of male C57BL/6J mice through a process involving liver cell isolation via perfusion with a balanced salt solution (25 mM HEPES, 121 mM NaCl, 4.7 mM KCl, 1.2 mM MgSO_4_, 5 mM NaHCO_3_, 2 mM CaCl_2_, 10 mM glucose, pH 7.4) containing 2% collagenase IV (17104019, Gibco, America) following anesthesia [18]. Cell debris was removed using Dulbecco's modified eagle medium (DMEM, KGM12800‐500, Keygen Biotech, China) supplemented with 50% Percoll (BS909, Biosharp, China) via gradient centrifugation.

The L02 cell line was acquired from the American Type Culture Collection, while the human embryonic kidney 293T (HEK‐293T) cell line was obtained from the Stem Cell Bank, Chinese Academy of Sciences. Primary hepatocytes, L02 cells, and HEK‐293T cells were cultured in DMEM supplemented with 10% (v/v) fetal bovine serum (FBS) under conditions of 5% CO_2_ and 37°C. Treatments administered to cells are detailed in the figure legends.

### 4.7 Nile Red and Oil Red Staining for Cells

For Nile Red staining, cells were first fixed with paraformaldehyde for 1 h. Following fixation, cells were treated with 1 mM Nile Red (C2051S, Beyotime, China) and Hoechst (P0133, Beyotime) for 10 min at room temperature before imaging using a confocal scanning microscope (Olympus, Japan). For Oil Red staining, cells were fixed with paraformaldehyde for 20 min at room temperature, washed with PBS, and then briefly treated with a 60% isopropanol solution for 20 s. Finally, cells were stained with 60% Oil Red O (C0158S, Beyotime) for 3 min at room temperature.

### 4.8 Western Blotting

Cell and tissue proteins were lysed using RIPA lysis buffer with 1% protease inhibitor cocktail. Nuclear extracts were obtained utilizing the NE‐PER Nuclear Cytoplasmic Extraction Reagent kit (78833, Thermo Scientific, USA). Total protein concentration was determined using a BCA assay kit (P0010, Beyotime) to ensure samples normalization. Protein extracts were resolved on 8%–15% SDS‐PAGE gels and transferred onto polyvinylidene fluoride (PVDF) membranes. The membranes were then blocked with 5% nonfat milk and subsequently probed with specific primary antibodies (STAT3 antibody, 9139T, CST, USA; Phospho‐Stat3 (Tyr705) antibody, 9145T, CST; β‐actin antibody, 81115‐1‐RR, Proteintech, USA; GAPDH antibody, 5174T, CST) overnight at 4°C. Following primary antibody incubation, membranes were treated with horse radish peroxidase‐conjugated secondary antibodies.

### 4.9 LC‐MS for Target Identification

The LC‐MS/MS analysis was performed on a Q‐Exactive Orbitrap mass spectrometer (RIGOL L‐3000) coupled with an Ultimate 3000 LC system. Appropriate amount of protein was added to the final concentration of 5 mM DTT, incubated at 37°C for 1 h, and then returned to room temperature. Iodoacetamide was added to a final concentration of 10 mM and light was avoided at room temperature for 45 min. Following diluting three times with ammonium bicarbonate (25 mM) and added with pancreatic enzyme according to the ratio of 50:1, and incubated at 37°C overnight, C18 desalting column was used to desalt 100% acetonitrile‐activated desalting column, 0.1% formic acid balanced column, loading samples onto the column, then washing the column with 0.1% formic acid, and finally elution with 70% acetonitrile, collecting the flow solution, and freeze‐drying. The mobile phase liquid A (100% water, 0.1% formic acid) and liquid B (80% acetonitrile, 0.1% formic acid) were prepared. Note that 1 µg supernatant sample was injected after dissolving lyophilized powder with 10 µL A solution and centrifuging at 14,000 *g* at 4°C for 20 min for liquid quality detection. Set the ion spray voltage at 2.4 kV and the ion transport tube temperature at 275°C using a QExactive HF‐X mass spectrometer and Nanospray Flex ion source. Data‐dependent acquisition mode was adopted for mass spectrum, and the full scanning range of mass spectrum was *m*/*z* 350–1500. The raw mass spectrometry detection data (.raw) was generated and analyzed by Mus musculus.

### 4.10 Transfection in Hepatocytes

The methods for transfection in hepatocytes have been described previously [37]. STAT3 plasmids and mutation plasmids were obtained from the Public Protein/Plasmid Library in China. The siRNA oligos are as follows:

Normal control (NC) siRNA: UUCUCCGAACGUGUCACGUTT

ACGUGACACGUUCGGAGAATT

STAT3 siRNA 1: GUUGAAUUAUCAGCUUAAA

UUUAAGCUGAUAAUUCAAC

### 4.11 Immunofluorescence

Primary hepatocytes were plated in cell dishes and fixed with a 4% paraformaldehyde solution for 15 min, followed by permeabilization with 0.2% Triton X‐100. Subsequently, the cells were incubated in blocking buffer for 60 min, followed by overnight incubation with primary antibodies at 4°C. Afterward, the cells were treated with secondary antibodies (Alexa Fluor 488 Secondary Antibody, A32731, Thermo Scientific, USA) for 60 min and stained with DAPI for 10 min before analysis using a confocal scanning microscope (Olympus).

### 4.12 Cellular Thermal Shift Assay (CETSA)

Primary hepatocytes were plated in dishes and treated with DMSO or carabrone for 6 h, and then frozen‐thawed three times with liquid nitrogen. The resulting cell lysate was incubated with 50 µM carabrone for 4–6 h. An aliquot of the lysate was sequentially heated with the increasing temperature (39, 42, 45, 48, 51, 54, 57, and 60°C). The lysates were centrifuged at 20,000 × *g* for 20 min at 4°C. The supernatants were added 1× SDS loading buffer and analyzed by immunoblotting.

### 4.13 Immunoprecipitation

In the case of Biotin–Cara pull‐down, cell lysates were collected in NP‐40 (P0013F, Beyotime) and preincubated with either biotin‐labeled carabrone or free biotin for 4 h. Subsequently, the lysates were incubated with Streptavidin Magarose Beads (SM007002, Smart‐Lifesciences, China) overnight at 4°C while being rotated. Afterward, the beads were washed three to four times and subjected to analysis via western blotting.

### 4.14 Molecular Docking

The x‐ray crystal structure of STAT3 (PDB ID: 6NJS) was downloaded from the PDB database. Molecular docking was performed with module of Covalent Docking (Schrödinger, 2019). The protein structure of STAT3 was prepared by the module of Protein Preparation Wizard. Amino acid residues in 20 Å range around the native ligand were defined as the binding pocket. The ligand carabrone was prepared by the module of Ligand Prepare. The prepared ligand was docked into STAT3 protein in the covalent‐docking mode. The covalent reaction type was set to Michael Addition, and the covalent modification residue was set to Cys328. Above procedures were performed with default parameters. After completion of the docking, the pose with the highest score was selected, and the binding mode was analyzed and imaged using pymol 2.4 [38].

### 4.15 SPR Binding Experiment

The binding kinetics of carabrone were assessed through SPR using a Biacore T200 instrument and its associated software (GE Healthcare, USA). STAT3 (10 µg/mL) was diluted in a 10 mM phosphate solution at different pH levels (5.5, 5.0, 4.5, and 4), then covalently attached to CM5 chips, achieving an immobilization density of 8000–10,000 resonance units. A reference surface was similarly prepared, omitting any protein solution. For immobilization, PBS‐P+ Buffer was utilized as the running buffer after activating the chip with 75 mg/mL of N‐(3‐dimethylaminopropyl)‐N'‐ethylcarbodiimide hydrochloride and 10 mg/mL of N‐hydroxysuccinimide, with the coupling reaction conducted at a flow rate of 30 µL/min. Binding affinity measurements were carried out at the same flow rate using the PBS‐P+ Buffer. Carabrone was evaluated in single cycle kinetics mode with the Biacore T200 control software, diluted in the running buffer, and injected at escalating concentrations over a contact period of 90 s. The resulting sensorgrams were processed and analyzed using the Biacore T200 evaluation software, and the binding curves were fitted to calculate the equilibrium dissociation constant (KD) [18].

### 4.16 STAT3 Knockout L02 Cells

STAT3 knockout cells (STAT3‐KO) were generated using CRISPR‐Cas9‐based gene editing. Briefly, sgRNAs targeting specific genes were cloned into pLV1cmv‐Cas9‐Puro‐U6 plasmid and lentivirus were prepared in 293T. L02 cells were infected with lentivirus using polybrene (10 mg/mL). After 48 h, transfected cells were selected by supplementing cell culture media with 2 mg/mL puromycin, and then seeded as single cells. STAT3 knockout clones were verified by western blotting.

### 4.17 Liver‐Specific Transfection in Mice

To induce liver‐specific overexpression of STAT3, mice were administered a 100 µL injection of AAV8‐TBG plasmid virus suspension (Hanbio Biotechnology, China) mixed with 100 µL of saline for a duration of 3 weeks by tail vein injection. Control mice received an injection of AAV8‐TBG normal control (NC).

### 4.18 Statistics

All data are presented as mean ± SEM. Comparisons were analyzed by using a two‐tailed Student's *t* test or one‐way analysis of variance (ANOVA). Significance was determined at *p* < 0.05 (GraphPad Prism version 8.0).

### 4.19 Chemical Synthesis of Probes and Derivatives in This Paper

The synthetic schemes and methods can be found in Supporting Information.

## Author Contributions

**An Pan**: writing – review and editing, writing – original draft, visualization, validation, supervision, funding acquisition, software, project administration, methodology, data curation, conceptualization. **Jiaming Jin**: writing – review and editing, writing – original draft, methodology, investigation, funding acquisition, data curation, methodology. **Yuze Wu**: methodology, investigation, data curation, conceptualization. **Huanhuan Chen**: methodology, investigation. **Yang Hu**: writing – review and editing, methodology, investigation, data curation. **Qiang Zhang**: methodology, investigation. **Wen Xiao**: methodology, investigation, data curation. **Anqi Shi**: methodology, investigation. **Yang Yang**: methodology, investigation. **Lina Jiang**: methodology. **Minghui Tan**: software, investigation. **Junwei Wang**: writing – review and editing, writing – original draft resources, project administration, funding acquisition, conceptualization. **Lihong Hu**: writing – review and editing, validation, project administration, funding acquisition, formal analysis, data curation, conceptualization. All authors have read and approved the final manuscript.

## Ethics Statement

All animal experiments were conducted in accordance with the China Laboratory Animal Welfare Guidelines and approved by the Animal Ethics Committee of Nanjing University of Chinese Medicine (approval number: 202304A065).

## Conflicts of Interest

The authors declare no conflicts of interest.

## Supporting information



Supporting Information: Figure S1‐S10, Table S1‐S4 and synthetic schemes and methods of compounds in this paper.

Supporting Information: Table S5 and 6. Excel file containing additional data too large to fit in a PDF, related to Figure 5C and Figure 5G.

## Data Availability

The datasets used and/or anaylzed during the present study are available from the corresponding author on reasonable request.
