# Supplementary material for: Carabrone Attenuates Metabolic Dysfunction–Associated Steatohepatitis by Targeting STAT3 in Mice
Source: MedComm (2020). 2025 Mar 10;6(3):e70145. doi: 10.1002/mco2.70145 (PMC11892021; doi:10.1002/mco2.70145)

**Supporting Information**

**Carabrone attenuates diet-induced non-alcoholic steatohepatitis by targeting STAT3 in mice**

An Pan,^a,b,†^ Jiaming Jin, ^b,c,†^ Yuze Wu,^b^ Qiang Zhang,^b^ Huanhuan Chen,^b^ Yang Hu,^b^ Wen Xiao,^b^ Anqi Shi,^b^ Yang Yang,^b^ Lina Jiang,^b^ Minghui Tan,^b^ Junwei Wang, ^a,b,^* Lihong Hu^b,^*

*^a^* State Key Laboratory on Technologies for Chinese Medicine Pharmaceutical Process Control and Intelligent Manufacture, Nanjing University of Chinese Medicine, Nanjing, 210023, P. R. China

*^b^* Jiangsu Key Laboratory for Functional Substance of Chinese Medicine, School of Pharmacy, Nanjing University of Chinese Medicine, Nanjing, 210023, P.R. China

*^c^* School of Life Science and Bioengineering, Jining University, Jining 273155, China

^†^ These authors contributed equally.

*Corresponding authors: jwwang@njucm.edu.cn; [lhhu@njucm.edu.cn](mailto:lhhu@njucm.edu.cn)

**Supplementary methods**

**Pari-feeding**

For pair-feeding experiments, we housed 2 mice with similar body weights in each cage according to a protocol approved by Vanderbilt IACUC. We measured food intake as g/100 g body weight from HFD-fed mice and gave that amount to carabrone-treated mice. We added food to the pair-feeding carabrone-treated mice at 10 am.

**Metabolic cage studies**

Mice were housed individually in metabolic cages with unlimited access to water and food and were maintained on a 12-h light/dark cycle at 24 °C or 30 °C. The mice were acclimated in the metabolic cages for 2 days before the start of the experiments. The VO_2_, VCO_2_, locomotor activity, food/water intake, and body weight were monitored with an O_2_/CO_2_ metabolic measuring system (Model MK-5000, Muromachi Kikai, Japan) for 2 consecutive days. The abbreviated Weir equation was used to calculate the 24-h energy expenditure (1.44 [3.9 × VO_2_ (mL/min) + 1.1 × VCO_2_ (mL/min)]).

**Supplemental information**

**Supplementary Figures**

**FIGURE S1**

**
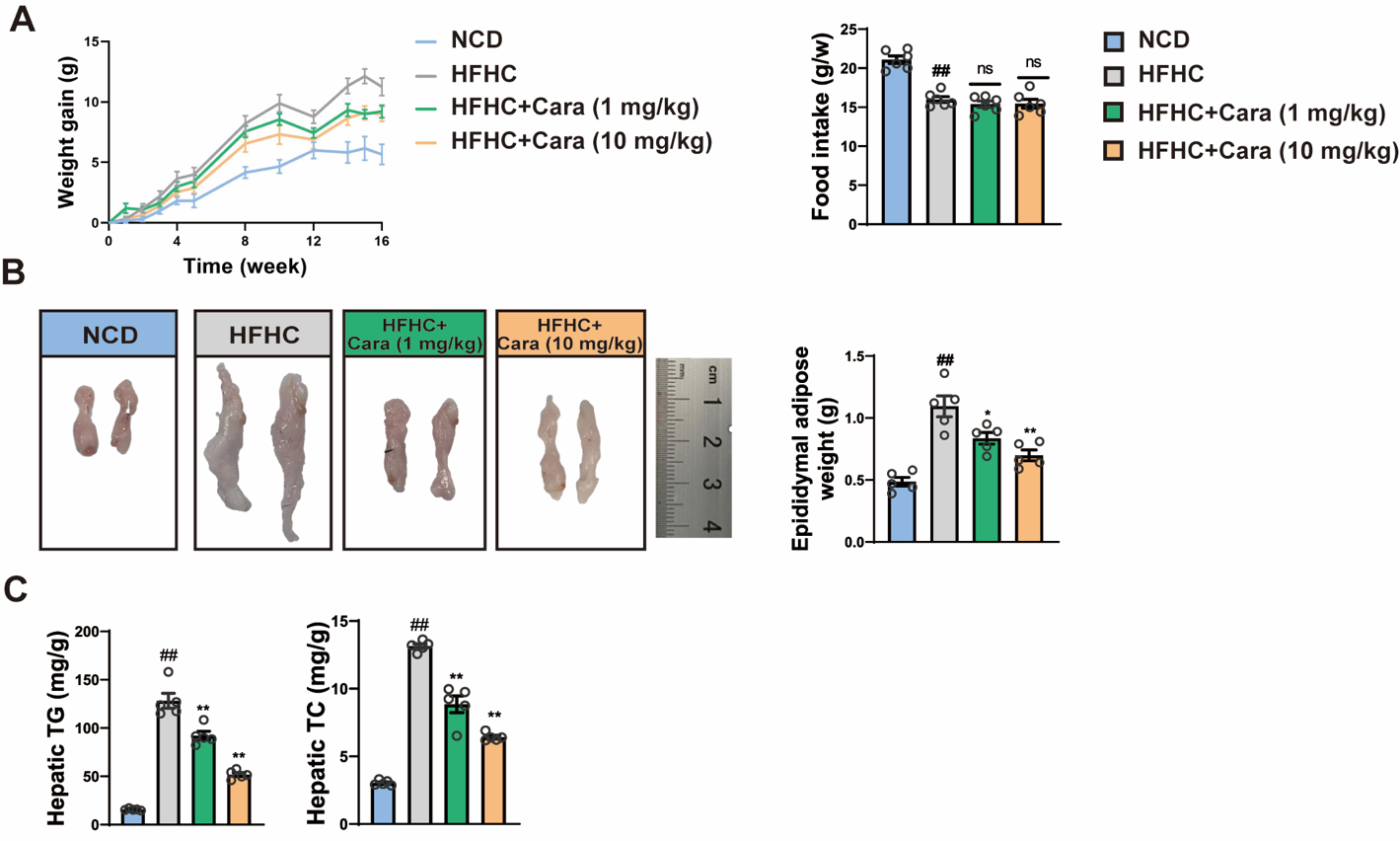
** **FIGURE S1. Carabrone blocks MASH progression in HFHC-induced mice.** Eight-week-old mice intragastrically administered 0.5% with CMCNa or carabrone (1 or 10 mg/kg) were fed with NCD or HFHC for 16 weeks. n = 5 per group. (A) Body weight gains. (B) The images and the weight of white adipose. (C) TG and TC levels in the livers. Values represent mean ± SEM. Statistical differences were determined by one-way ANOVA. #*p* < 0.05, ##*p* < 0.01 compared with NCD group; **p* < 0.05, ***p* < 0.01 compared with HFHC group.

**FIGURE S2**


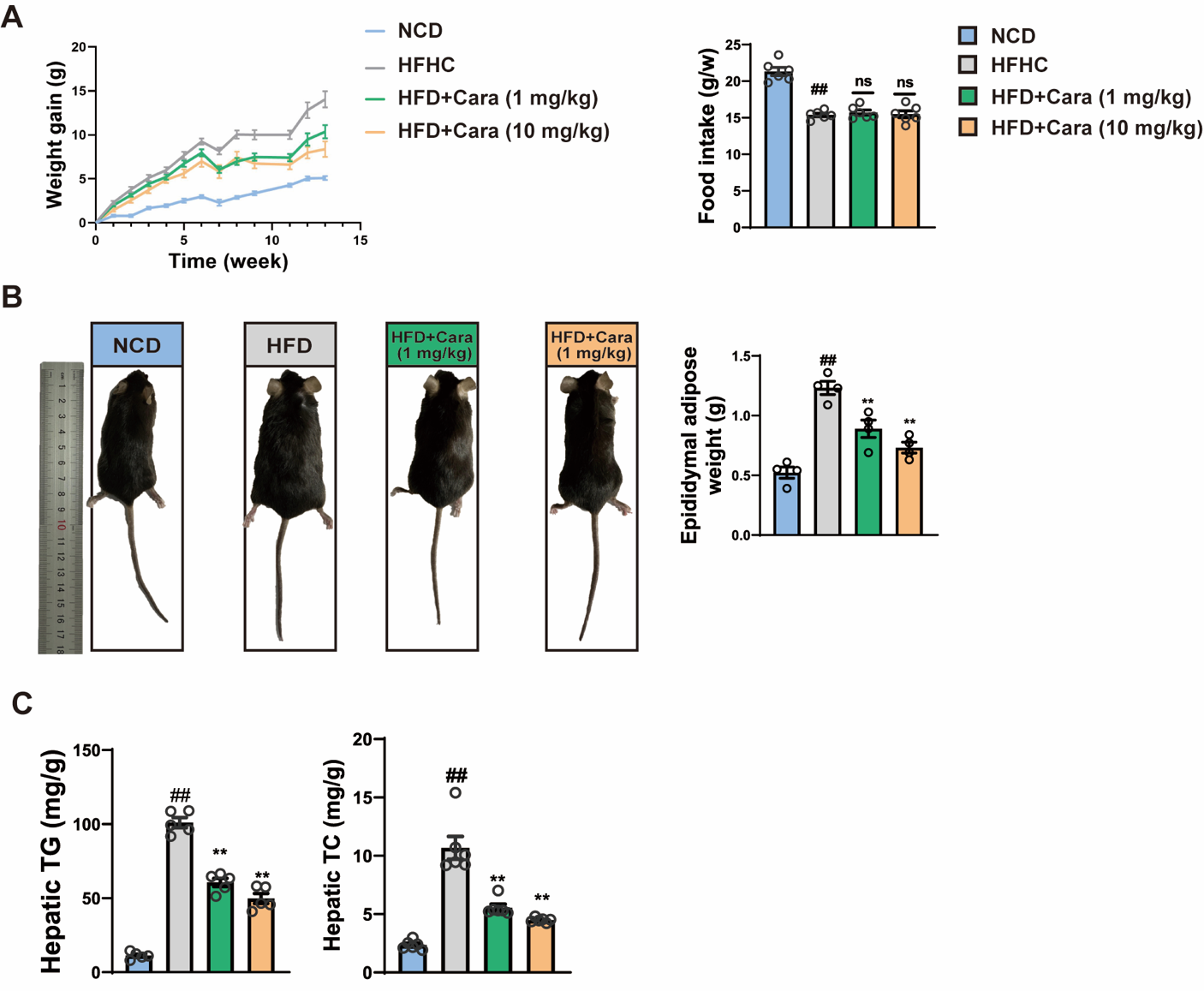


**FIGURE S2. Carabrone blocks MASH progression in HFD-induced mice.** Eight-week-old mice intragastrically administered 0.5% with CMCNa or carabrone (1 or 10 mg/kg) were fed with NCD or HFD for 16 weeks. (A) Body weight gains. (B) Body size and the weight of white adipose. (C) TG (n=5 per group) and TC (n=6 per group) levels in the livers. Values represent mean ± SEM. Statistical differences were determined by one-way ANOVA. #p < 0.05, ##p < 0.01 compared with NCD group; **p* < 0.05, ***p* < 0.01 compared with HFD group.

**FIGURE S3**

**
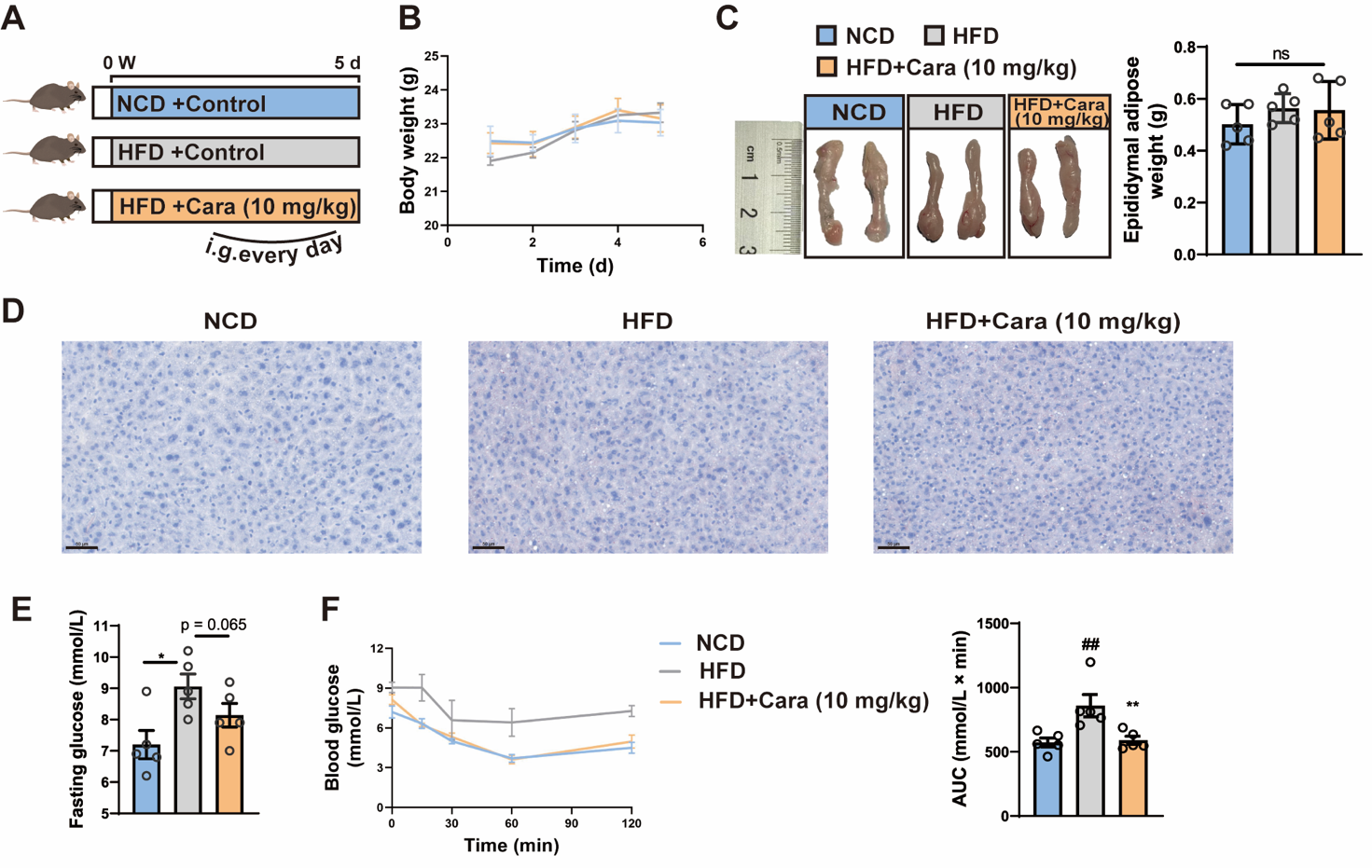
**

**FIGURE S3. Carabrone improved glucose metabolism in short-period HFD mice.** Eight-week-old mice intragastrically administered 0.5% with CMCNa or carabrone (10 mg/kg) were fed with HFD or NCD for 5 days. n = 5 per group. (A) Scheme illustrating carabrone treatment strategy. (B) Body weight. (C) The images and the weight of white adipose. (D) Representative images of Oil Red O-stained liver sections. Scale = 50 µm. (E) Fasting glucose. (F) Insulin tolerance test (0.75 U/kg). AUC is indicated on the right. Values represent mean ± SEM. Statistical differences were determined by one-way ANOVA. #p < 0.05, ##p < 0.01 compared with NCD group; **p* < 0.05, ***p* < 0.01 compared with HFD group.

**FIGURE S4**


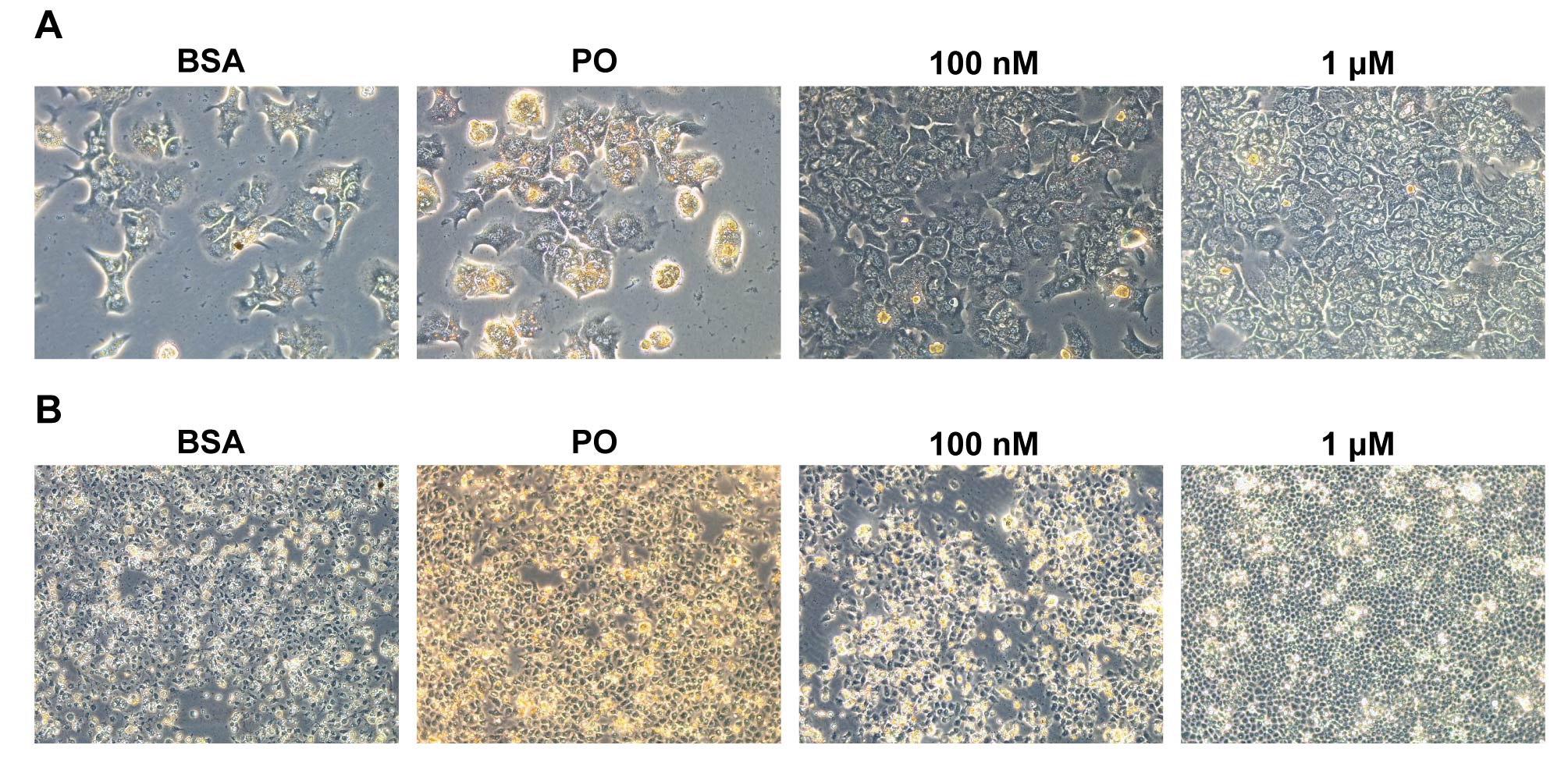


**FIGURE S4.** **Carabrone attenuates PO-stimulated lipid accumulation in hepatocytes.** (A) Representative oil red O staining images of PO-stimulated primary hepatocytes treated with carabrone (0.1 and 1 μM) for 24 h. (B) Representative oil red O staining images of PO-stimulated L02 cells treated with carabrone (0.1 and 1 μM) for 24 h.

**FIGURE S5**


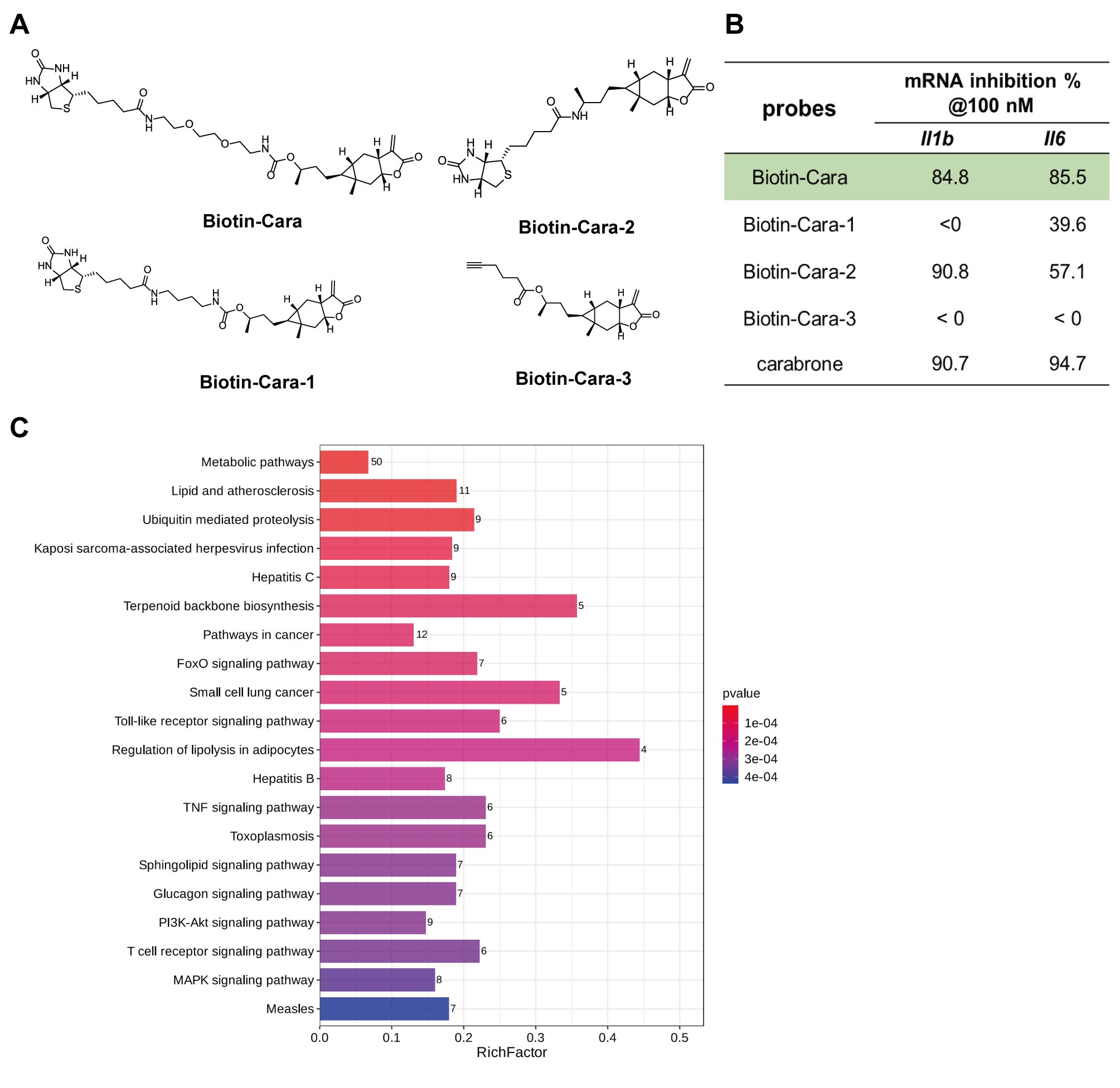


**FIGURE S5.** **STAT3 is a direct target of Carabrone in hepatocytes.** (A) Structure of probes and (B) their anti-MASH activities in primary hepatocytes. (C) KEGG analysis of the pathways associated with carabrone-interacted proteins.

**FIGURE S6**

**
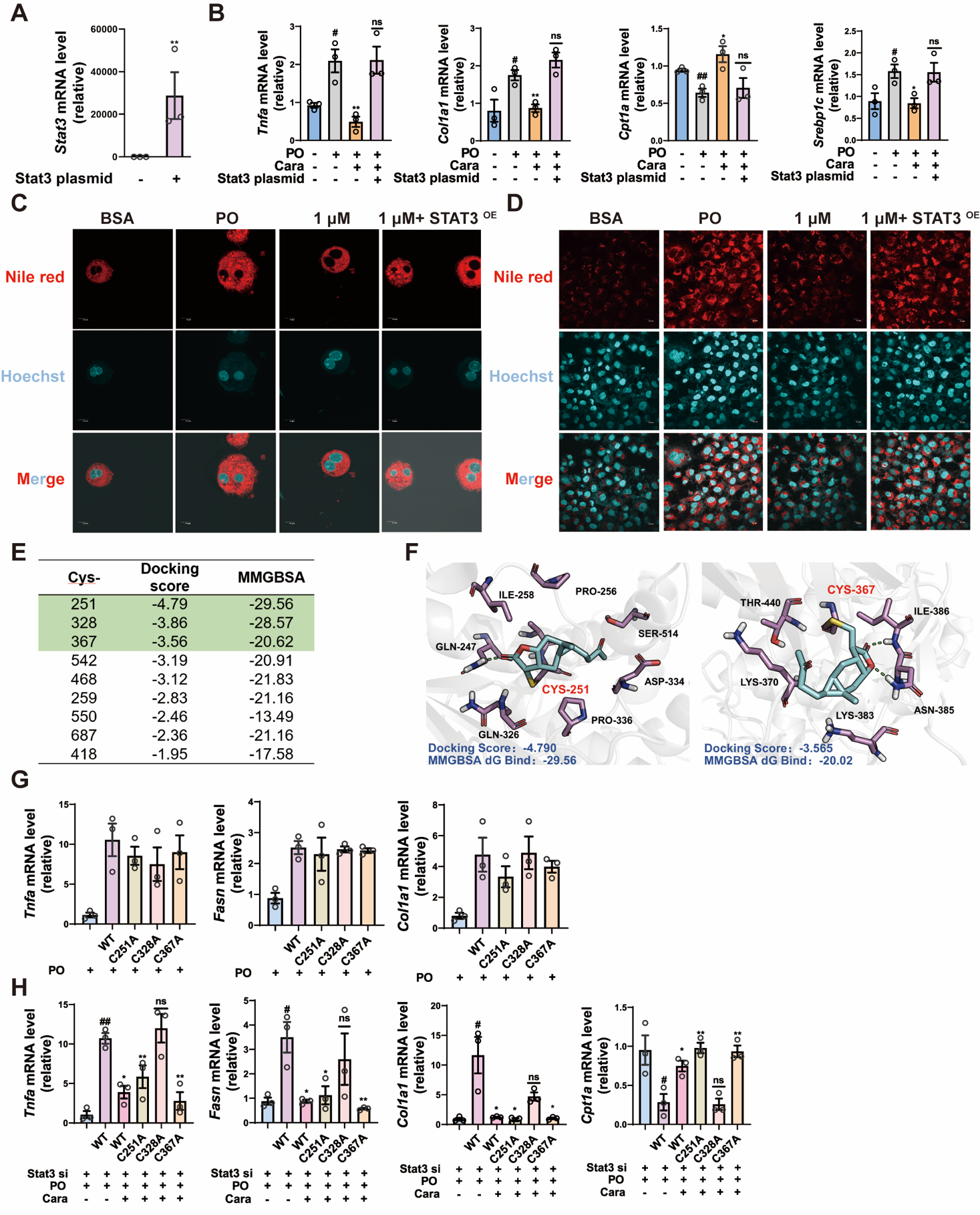
**

**FIGURE S6. Direct inactivation of STAT3 by Carabrone.** (A) mRNA levels of *Stat3* in primary hepatocytes with or without STAT3 plasmid transfection. n = 3 per group. (B) mRNA levels of *Tnfa*, *Col1a1*, *Srebp1c* and *Cpt1a* in PO-stimulated primary hepatocytes treated with carabrone (1 μM) for 24 h, with or without STAT3 plasmid transfection. n = 3 per group. (C) Representative nile red staining images of PO-stimulated primary hepatocytes treated with carabrone (1 μM) for 24 h, with or without STAT3 plasmid transfection. Scale = 10 µm. (D) Representative nile red staining images of PO-stimulated L02 cells treated with carabrone (1 μM) for 24 h, with or without STAT3 plasmid transfection. (E) Score of covalent docking with different cysteine residues as reaction sites. (F) Binding modes and sites of carabrone with STAT3 predicted by Schrödinger. (G) mRNA levels of *Tnfa*, *Fasn* and *Col1a1* in PO-stimulated primary hepatocytes transfected with plasmids encoding the WT, C251A, C328A, C367A mutation of STAT3. n = 3 per group. (H) mRNA levels of genes *Tnfa*, *Fasn*, *Col1a1* and *Cpt1a* transfected with plasmids encoding the WT, C251A, C328A, C367A mutation of STAT3, with or without carabrone treatment (1 mM, 24 h). n = 3 per group. Values represent mean ± SEM. Statistical differences were determined by one-way ANOVA. ^#^*p* < 0.05, ^##^*p* < 0.01 compared with control group; **p* < 0.05, ***p* < 0.01 compared with PO group.

**FIGURE S7**


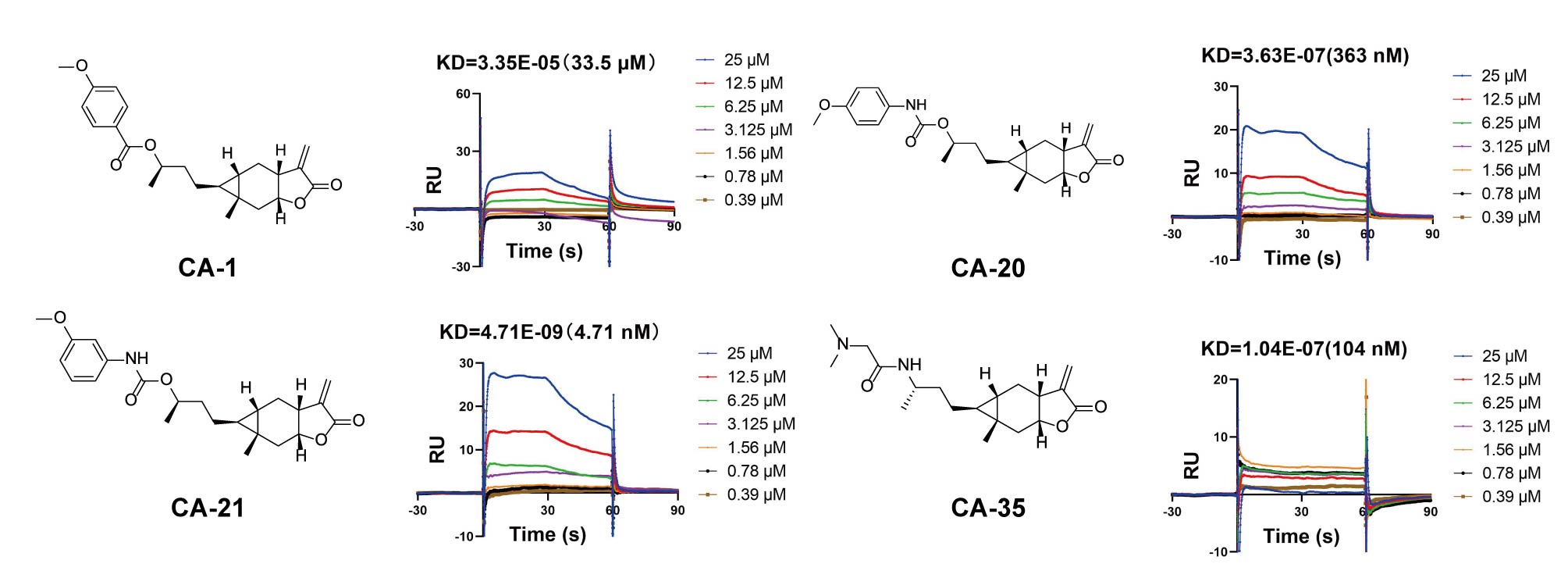


**FIGURE S7. The binding affinity between carabrone derivatives and STAT3.**

**FIGURE S8**
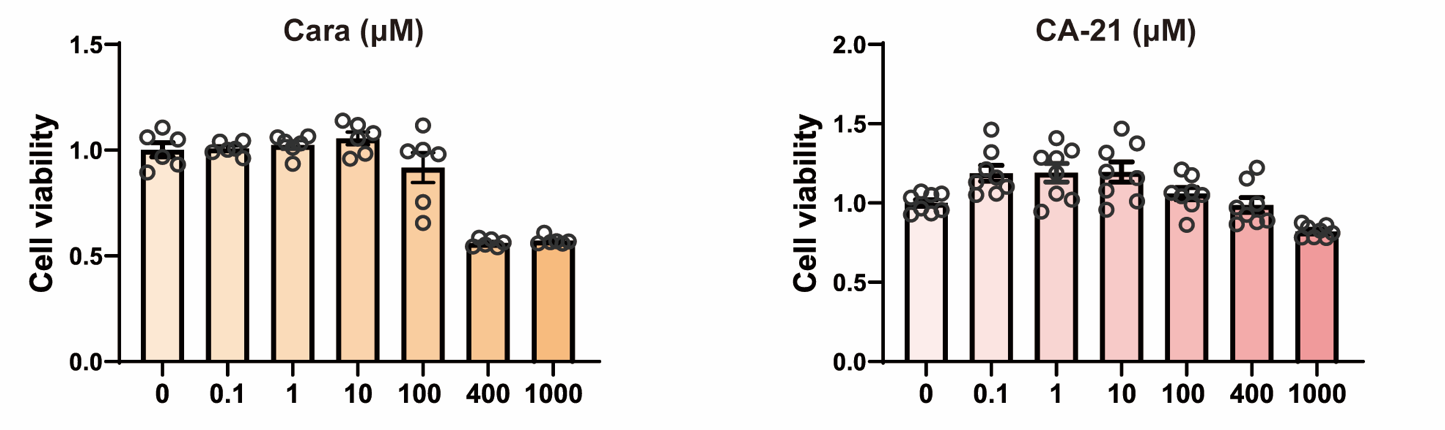
 **FIGURE S8. The CCK-8 assay of carabrone and CA-21 in primary hepatocytes.**

**FIGURE S9**


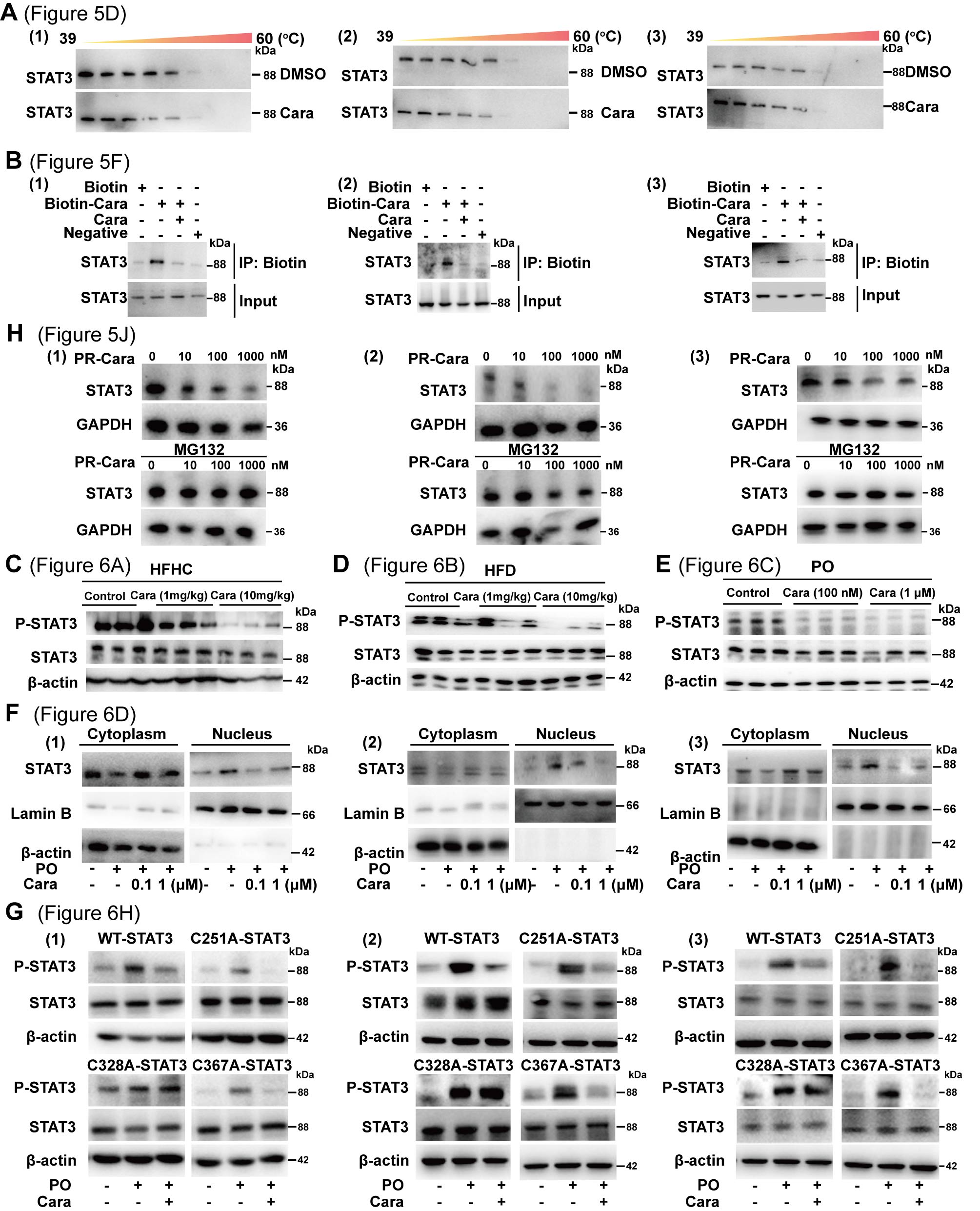


**FIGURE S9. Triplicate western blots.**

**FIGURE S10**


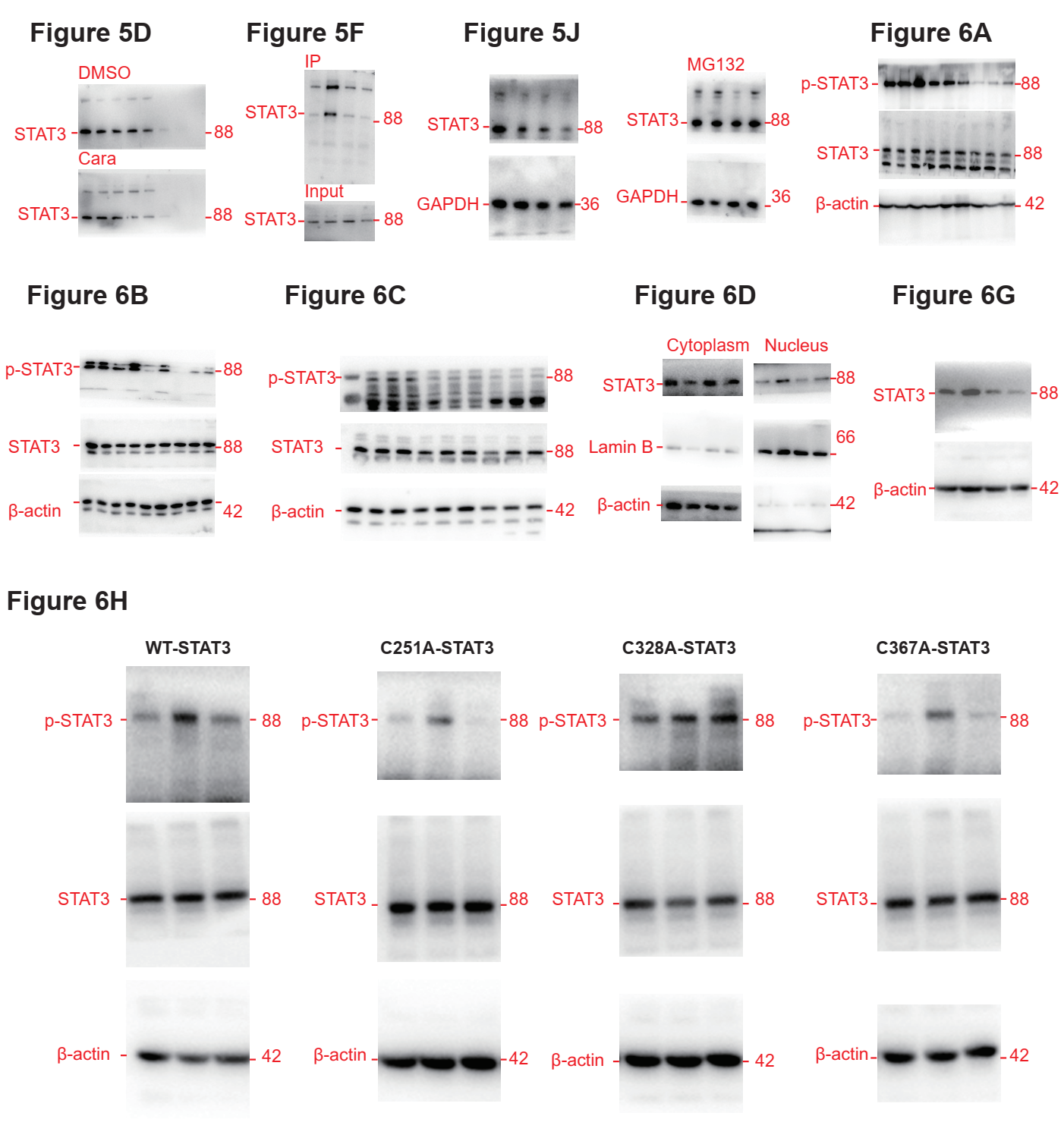


**FIGURE S10. The original, uncropped western blot images.**

**Table S1** Primers for qPCR

| **Gene** | **Forward Sequence (5’-3’)** | **Reverse Sequence (5’-3’)** |
| --- | --- | --- |
| Mouse *Fasn* | CTGCGGAAACTTCAGGAAATG | GGTTCGGAATGCTATCCAGG |
| Mouse *Pparα* | TATTCGGCTGAAGCTGGTGTAC | CTGGCATTTGTTCCGGTTCT |
| Mouse *Cpt1a* | AGGACCCTGAGGCATCTATT | ATGACCTCCTGGCATTCTCC |
| Mouse *Mcp1* | TACAAGAGGATCACCAGCAGC | ACCTTAGGGCAGATGCAGTT |
| Mouse *Cxcl10* | ATGACGGGCCAGTGAGAATG | ATGATCTCAACACGTGGGCA |
| Mouse *Il6* | TAGTCCTTCCTACCCCAATTTCC | TTGGTCCTTAGCCACTCCTTC |
| Mouse *Il1b* | CCGTGGACCTTCCAGGATGA | GGGAACGTCACACACCAGCA |
| Mouse *Tnfa* | CATCTTCTCAAAATTCGAGTGACAA | TGGGAGTAGACAAGGTACAACCC |
| Mouse *Col1a1* | TGCTAACGTGGTTCGTGACCGT | ACATCTTGAGGTCGCGGCATGT |
| Mouse *Col3a1* | ACGTAAGCACTGGTGGACAG | CCGGCTGGAAAGAAGTCTGA |
| Mouse *Ctgf* | TGACCCCTGCGACCCACA | TACACCGACCCACCGAAGACACAG |
| Mouse *Actb* | GTGACGTTGACATCCGTAAAGA | GCCGGACTCATCGTACTCC |
| *Mouse Srebp1c* | GGAGCCATGGATTGCACATT | GGCCCGGGAAGTCACTGT |
| Mouse *Acox1* | TAACTTCCTCACTCGAAGCCA | AGTTCCATGACCCATCTCTGTC |
| Mouse *Stat3* | CAATACCATTGACCTGCCGAT | GAGCGACTCAAACTGCCCT |
| Human *FASN* | ACAGCGGGGAATGGGTACT | GACTGGTACAACGAGCGGAT |
| Human *ACOX1* | ACTCGCAGCCAGCGTTATG | AGGGTCAGCGATGCCAAAC |
| Human *PPARA* | TACTGTCGGTTTCAGAAATGCC | GTCAGCGGACTCTGGATTCAG |
| Human *IL6* | GAGTAGTGAGGAACAAGCCAGA | AAGCTGCGCAGAATGAGATGA |
| Human *MCP1* | CAGCCAGATGCAATCAATGCC | TGGAATCCTGAACCCACTTCT |
| Human SREBP1C | ACAGTGACTTCCCTGGCCTAT | GCATGGACGGGTACATCTTCAA |
| Human *ACTA2* | CTATGAGGGCTATGCCTTGCC | GCTCAGCAGTAGTAACGAAGGA |
| Human *CXCL10* | GTGGCATTCAAGGAGTACCTC | TGATGGCCTTCGATTCTGGATT |
| Human *COL1A1* | GAGGGCCAAGACGAAGACATC | CAGATCACGTCATCGCACAAC |
| Human *CPT1A* | TCCAGTTGGCTTATCGTGGTG | TCCAGAGTCCGATTGATTTTTGC |
| Human *TNFA* | TGGCGTGGAGCTGAGAGATA | TGATGGCAGAGAGGAGGTTG |
| Human *ACTB* | CATGTACGTTGCTATCCAGGC | CTCCTTAATGTCACGCACGAT |

**Table S2** Effect of carabrone derivatives **CA-1**~**CA-5** on the expression of *Il1b* /*Il6* mRNA in PO-stimulated primary hepatocytes.

| **compound** | **R** | **mRNA inhibition %**  **@100 nM** | | **compound** | **R** | **mRNA inhibition %**  **@100 nM** | |
| --- | --- | --- | --- | --- | --- | --- | --- |
|  |  | **Il1b** | **Il6** |  |  | **Il1b** | **Il6** |
| **CA-1** |  | 94.7 | 82.7 | **CA-4** |  | 32.9 | 64.6 |
| **CA-2** |  | 58.6 | 76.2 | **CA-5** |  | 20.9 | 51.4 |
| **CA-3** |  | 79.5 | 93.0 | **Cara** | - | 85.4 | 88.9 |

**Table S3** Effect of carabrone derivatives **CA-6**~**CA-28** on the expression of *Il1b* /*Il6* mRNA in PO-stimulated primary hepatocytes.

| **compound** | **R** | **mRNA inhibition %**  **@100 nM** | | **compound** | **R** | **mRNA inhibition %**  **@100 nM** | |
| --- | --- | --- | --- | --- | --- | --- | --- |
|  |  | **Il1b** | **Il6** |  |  | **Il1b** | **Il6** |
| **CA-6** |  | 11.1 | 78.1 | **CA-18** |  | 40.2 | 12.2 |
| **CA-7** |  | 54.2 | 67.6 | **CA-19** |  | 24.0 | 0.8 |
| **CA-8** |  | 49.7 | 66.4 | **CA-20** |  | 95.3 | 97.5 |
| **CA-9** |  | 70.3 | 80.6 | **CA-21** |  | 97.6 | 97.0 |
| **CA-10** |  | 102.8 | 44.4 | **CA-22** |  | 20.0 | 40.3 |
| **CA-11** |  | 31.5 | 34.8 | **CA-23** |  | 77.4 | 87.2 |
| **CA-12** |  | 81.6 | 72.2 | **CA-24** |  | <0 | 43.8 |
| **CA-13** |  | 75.2 | 83.8 | **CA-25** |  | 53.6 | 33.6 |
| **CA-14** |  | 87.2 | 89.5 | **CA-26** |  | 17.6 | 8.6 |
| **CA-15** |  | 54.5 | 68.9 | **CA-27** |  | 94.7 | 59.8 |
| **CA-16** |  | 22.3 | 88.5 | **CA-28** |  | <0 | 31.6 |
| **CA-17** |  | 58.4 | 118.1 | **carabrone** | - | 92.2 | 86.1 |

**Table S4** Effect of carabrone derivatives **CA-29**~**CA-36** on the expression of *Il1b* /*Il6* mRNA in PO-stimulated primary hepatocytes.

| **compound** | **R** | **mRNA inhibition %**  **@100 nM** | | **compound** | **R** | **mRNA inhibition %**  **@100 nM** | |
| --- | --- | --- | --- | --- | --- | --- | --- |
|  |  | ***Il1b*** | ***Il6*** |  |  | ***Il1b*** | ***Il6*** |
| **CA-29** |  | 83.6 | 83.2 | **CA-34** |  | 36.0 | 8.9 |
| **CA-30** |  | 56.0 | 11.7 | **CA-35** |  | 83.5 | 91.3 |
| **CA-31** |  | 24.0 | 0.8 | **CA-36** |  | 74.6 | 48.2 |
| **CA-32** |  | 61.4 | 88.3 | **carabrone** | - | 92.3 | 94.2 |
| **CA-33** |  | 3.1 | <0 |  |  |  |  |

# Synthetic schemes and methods of compounds in this paper

**Carabrone and carabrol in this paper were extracted from *Carpesium abrotanoides* L.**

**Carabrone**: white solid, ^1^H NMR (500 MHz, CDCl_3_) *δ* (ppm): 6.26 (d, *J* = 2.9 Hz, 1H), 5.57 (d, *J* = 2.4 Hz, 1H), 4.82-4.77 (m, 1H), 3.20-3.14 (m, 1H), 2.55 (t, *J* = 7.5 Hz, 2H), 2.38-2.32 (m, 2H), 2.18 (s, 3H), 1.66-1.63 (m, 1H), 1.58-1.54 (m, 1H), 1.08 (s, 3H), 1.00-0.90 (m, 2H), 0.49-0.45 (m, 1H), 0.41-0.37 (m, 1H). ^13^C NMR (125 MHz, CDCl_3_) *δ* (ppm): 208.68, 170.47, 139.00, 122.56, 75.60, 43.58, 37.73, 37.29, 34.23, 30.73, 30.09, 23.35, 22.91, 18.23, 17.22. HRMS (ESI): m/z [M+Na]^+^ Calcd for C_15_H_20_NaO_3_ 271.1310, Found 271.1344.

**Carabrol:** yellow liquid, ^1^H NMR (500 MHz, CDCl_3_) *δ* (ppm): 6.26 (d, *J* = 2.7 Hz, 1H), 5.57 (d, *J* = 2.3 Hz, 1H), 4.83-4.78 (m, 1H), 3.84 (q, *J* = 6.2 Hz, 1H), 3.21-3.15 (m, 1H), 2.40-2.32 (m, 2H), 1.61-1.52 (m, 2H), 1.45 (s, 1H), 1.42-1.35 (m, 2H), 1.22 (d, *J* = 6.1 Hz, 3H), 1.10 (s, 3H), 1.02-0.92 (m, 2H), 0.50-0.46 (m, 1H), 0.39-0.35 (m, 1H). ^13^C NMR (125 MHz, CDCl_3_) *δ* (ppm): 170.56, 139.12, 122.50, 75.74, 67.90, 39.30, 37.81, 37.43, 35.02, 30.87, 25.38, 23.64, 22.93, 18.32, 17.12. HRMS (ESI): m/z [M+H]^+^ Calcd for C_15_H_23_O_3_ 251.1647, Found 251.1675.

**Figure S11** Synthetic schemes for **Biotin-Cara**

Reagents and conditions: a) i) 1,1'-carbonyl-di(1,2,4-triazole), 40^o^C, 1.5 h, ii) boc-1-amino-3,6-dioxa-8-octanediamine, CH_2_Cl_2_, 20^o^C, 6.5 h; b) CF_3_COOH, CH_2_Cl_2_ 20^o^C, 2 h; c) (+)-biotin-*N*-hydroxysuccimide ester, Et_3_N, DMF, 20^o^C, 4h.

**Synthesis of intermediate 1**

To a solution of Carabrol (100 mg, 0.4mmol) in CH_2_Cl_2_ (2.5 mL) was added 1,1'-Carbonyl-di(1,2,4-triazole) (98.4 mg, 0.6 mmol). The mixture was warmed to 40^o^C and stirred at 40^o^C for 1.5 h, then it was cooled to room temperature and boc-1-amino-3,6-dioxa-8-octanediamine (135 mg, 0.72 mmol) was added. The mixture was stirred at room temperature for 6.5 h until completion of the reaction as indicated by TLC, then it was diluted with water and extracted with EA. The combined organic phase was washed with brine, and dried over anhydrous Na_2_SO_4_. The solvent was evaporated to give crude product, which was purified by silica gel column chromatography (PE/ EA = 8:1 to 2:1) to give intermediate **1** as a yellow liquid (146 mg, 76%). ^1^H NMR (500 MHz, CDCl_3_) *δ* (ppm): 6.26 (d, *J* = 2.4 Hz, 1H), 5.57 (d, *J* = 1.8 Hz, 1H), 5.20 (s, 1H), 5.09 (s, 1H), 4.83-4.77 (m, 2H), 3.63 (s, 4H), 3.57 (q, *J* = 12.0 Hz, 4H), 3.41-3.38 (m, 2H), 3.37-3.33 (m, 2H), 3.20-3.14 (m, 1H), 2.39-2.31 (m, 2H), 1.73-1.68 (m, 1H), 1.59-1.55 (m, 1H),1.46 (s, 9H), 1.37-1.30 (m, 2H), 1.23 (d, *J* = 5.9 Hz, 3H), 1.08 (s, 1H), 1.00-0.90 (m, 2H), 0.48-0.43 (m, 1H), 0.37-0.33 (m, 1H).

**Synthesis of Intermediate 2**

To a solution of **1** (100 mg, 0.19 mmol) in CH_2_Cl_2_ (2 mL) was added trifluoroacetic acid (0.5 mL). The mixture was stirred at room temperature for 2 h until completion of the reaction as indicated by TLC, then it was evaporated under a vacuum to give crude intermediate **2** as a yellow liquid (85 mg, 89%), which was used in the next step without further purification.

**Synthesis of Biotin-Cara**

To a solution of **2** (60 mg, 0.12 mmol) in DMF (2 mL) were added (+)-biotin-*N*-hydroxysuccimide ester (45 mg, 0.13 mmol) and TEA (37 mg, 0.36 mmol). The mixture was stirred at room temperature for 4 h until completion of the reaction as indicated by TLC, then it was diluted with water and extracted with EA. The combined organic phase was washed with brine, and dried over anhydrous Na_2_SO_4_. The solvent was evaporated to give crude product, which was purified by silica gel column chromatography (CH_2_Cl_2_/ MeOH = 150:1 to 50:1) to give **Biotin-Cara** as a white solid (40 mg, 51%).^1^H NMR (500 MHz, CDCl_3_) *δ* (ppm): 6.25 (s,1H), 6.19 (s, 1H),5.58 (s, 1H), 5.32 (s, 1H), 4.92 (s, 1H), 4.80 (s, 1H), 4.54 (s, 1H), 4.34 (s, 1H), 3.27 (s, 2H), 3.18 (s, 4H), 2.98-2.91 (m, 1H), 2.75 (d, *J* = 12.8 Hz, 2H), 2.38-2.32 (m, 2H), 2.24-2.22 (m, 2H), 1.72 -1.65(m, 4H), 1.55 (m, 5H),1.48-1.44 (m, 2H), 1.39-1.32 (m, 2H), 1.22 (d, *J* = 4.0 Hz, 3H), 1.08 (s, 1H), 1.01-0.92 (m, 2H), 0.49-0.44 (m, 1H), 0.38-0.34 (m, 1H). ^13^C NMR (126 MHz, CDCl3) *δ* (ppm): 173.43, 170.63, 164.21, 156.64, 139.08, 122.62, 75.78, 71.09, 61.81, 60.24, 55.78, 40.57, 39.07, 37.77, 37.40, 36.25, 35.95, 34.73, 30.81, 28.23, 28.05, 27.51, 26.77, 25.73, 25.04, 22.88, 20.35, 18.28, 17.06. HRMS (ESI): m/z [M-H]^-^ Calcd for C_30_H_45_N_4_O_6_S 589.3060, Found 589.3031.

**Figure S12** Synthetic schemes for **Biotin-Cara-1**

Reagents and conditions: a) i) 1,1'-carbonyl-di(1,2,4-triazole), 40^o^C, 1.5 h, ii) *tert*-butyl-*N*-(4-aminobutyl) carbamate, CH_2_Cl_2_, 20^o^C, 4 h; b) CF_3_COOH, CH_2_Cl_2_ 20^o^C, 2 h; c) (+)-biotin-*N*-hydroxysuccimide ester, Et_3_N, DMF, 20^o^C.

**Synthesis of Intermediate** **3**

The title compound was prepared from intermediate *tert*-butyl-*N*-(4-aminobutyl) carbamate according to the synthesis procedure of intermediate **1**. The reaction crude product was purified by silica gel chromatography to give intermediate **3** (120 mg, 65%) as a colorless liquid. ^1^H NMR (500 MHz, CDCl_3_) *δ* (ppm): 6.25 (s, 1H), 5.57 (s, 1H), 4.82-4.80 (m, 1H), 4.68 (s, 1H), 4.59 (s, 1H), 3.19-3.14 (m, 5H), 2,39-2,31 (m, 2H), 1.69-1.65 (m, 2H),1.53 (s, 4H), 1.45 (s, 9H), 1.38-1.27 (m, 2H), 1.22 (d, *J* = 5.7 Hz, 3H), 1.08 (s, 3H), 1.00-0.91 (m, 2H), 0.47-0.44 (m, 1H), 0.37-0.34 (m, 1H).

**Synthesis of** **Intermediate** **4**

The title compound was prepared from intermediate **3** according to the synthesis procedure of intermediate **2**. The reaction mixture was evaporated under a vacuum to give crude intermediate **4** as a yellow liquid (70 mg, 88%), which was used in the next step without further purification.

**Synthesis of Biotin-Cara-1**

The title compound was prepared from intermediate **4** according to the synthesis procedure of **Biotin-Cara**. The reaction crude product was purified by silica gel chromatography to give **Biotin-Cara-1** (35 mg, 49%) as a white solid. ^1^H NMR (500 MHz, CDCl_3_) *δ* (ppm): 6.25 (s,1H), 6.19 (s, 1H),5.58 (s, 1H), 5.32 (s, 1H), 4.92 (s, 1H), 4.80 (s, 1H), 4.54 (s, 1H), 4.34 (s, 1H), 3.27 (s, 2H), 3.18 (s, 4H), 2.98-2.91 (m, 1H), 2.75 (d, *J* = 12.8 Hz, 2H), 2.38-2.32 (m, 2H), 2.24-2.22 (m, 2H), 1.72 -1.65(m, 4H), 1.55 (m, 5H),1.48-1.44 (m, 2H), 1.39-1.32 (m, 2H), 1.22 (d, *J* = 4.0 Hz, 3H), 1.08 (s, 1H), 1.01-0.92 (m, 2H), 0.49-0.44 (m, 1H), 0.38-0.34 (m, 1H). ^13^C NMR (126 MHz, CDCl3) *δ* (ppm): 173.43, 170.63, 164.21, 156.64, 139.08, 122.62, 75.78, 71.09, 61.81, 60.24, 55.78, 40.57, 39.07, 37.77, 37.40, 36.25, 35.95, 34.73, 30.81, 28.23, 28.05, 27.51, 26.77, 25.73, 25.04, 22.88, 20.35, 18.28, 17.06. HRMS (ESI): m/z [M-H]^-^ Calcd for C_30_H_45_N_4_O_6_S 589.3060, Found589.3031.

**Figure S13** Synthetic schemes for **Biotin-Cara-2**

Reagents and conditions: a) (Boc)_2_NH, PPh_3_, DIAD, THF, 20^o^C, 1.5 h; b) CF_3_COOH, CH_2_Cl_2_ 20^o^C, 2 h; c) (+)-biotin-*N*-hydroxysuccimide ester, Et_3_N, DMF, 20^o^C, 4 h.

**Synthesis of** **Intermediate 5**

To a solution of carabrol (500 mg, 2 mmol) in anhydrous THF (8 mL) were added di-*tert*-butyl iminodicarboxylate (651.8 mg, 3 mmol), PPh_3_ (786.9 mg, 3 mmol) and DIAD (606.6 mg, 3 mmol), the reaction mixture was stirred at 20^o^C in N_2_ environment for 1.5 h until completion of the reaction as indicated by TLC, then it was diluted with water and extracted with EA. The combined organic phase was washed with brine, and dried over anhydrous Na_2_SO_4_. The solvent was evaporated to give crude product, which was purified by silica gel column chromatography (PE/ EA = 10:1 to 5:1) to give intermediate **5** as a white solid (360 mg, 40%).^1^H NMR (500 MHz, CDCl_3_) *δ* (ppm): 6.25 (d, *J* = 2.5 Hz, 1H), 5.57 (d, *J* = 1.8 Hz, 1H), 4.82-4.77 (m, 1H), 4.28-4.21 (m, 1H), 3.19-3.14 (m, 1H), 2.40-2.31 (m, 2H), 2.00-1.92 (m, 1H), 1.51 (s, 18H), 1.39-1.34 (m, 1H), 1.30 (d, *J* = 6.8 Hz, 3H), 1.27-1.21 (m, 1H), 1.08 (s, 3H), 0.99-0.89 (m, 2H), 0.46-0.43 (m, 1H), 0.37-0.33 (m, 1H)。

**Synthesis of** **Intermediate 6**

To a solution of intermediate **5** (200 mg, 0.45 mmol) was added trifluoroacetic acid (2 mL), the mixture was stirred at room temperature for 2 h until completion of the reaction as indicated by TLC, then it was evaporated under a vacuum to give crude intermediate **6** as a yellow liquid (140 mg, 90%), which was used in the next step without further purification.

**Synthesis of Biotin-Cara-2**

The title compound was prepared from intermediate **6** according to the synthesis procedure of **Biotin-Cara**. The reaction crude product was purified by silica gel chromatography to give **Biotin-Cara-2** (38 mg, 57%) as a white solid. ^1^H NMR (500 MHz, CDCl_3_) *δ* (ppm): 6.52 (s, 1H), 6.23 (d, *J* = 2.0 Hz, 1H), 5.91 (d, *J* = 8.6 Hz, 1H), 5.76 (s, 1H), 5.57 (d, *J* = 1.4 Hz), 4.81-4.76 (m, 1H), 4.52 (d, *J* = 6.5 Hz, 1H), 4.31 (d, *J* = 5.8 Hz, 1H), 4.02-3.97 (m, 1H), 3.17-3.15 (m, 2H), 2.91 (dd, *J* = 12.8 Hz, 4.7 Hz, 1H), 2.74 (d, *J* = 12.8 Hz, 1H), 2.41-2.50 (m, 1H), 2.30 (dd, *J* =13.7 Hz, 6.1 Hz, 1H), 2.21-2.17 (m, 2H), 1.77-1.63 (m, 4H), 1.52 (q, *J* = 7.4 Hz, 1H), 1.45-1.38 (m, 3H), 1.30-1.21 (m, 2H),1.13 (d, *J* = 6.6 Hz, 3H), 1.06 (s, 3H), 0.99-0.87 (m, 2H), 0.43-0.40 (m, 1H), 0.38-0,34 (m, 1H). ^13^C NMR (126 MHz, CDCl3) *δ* (ppm): 172.46, 170.64, 164.00, 139.11, 122.62, 75.80, 61.82, 60.22, 55.70, 44.73, 40.57, 37.75, 37.40, 36.99, 36.26, 34.71, 30.84, 28.23, 28.11, 25.83, 25.66, 23.02, 21.32, 18.40, 16.85. HRMS (ESI): m/z [M-H]^-^ Calcd for C_25_H_36_N_3_O_4_S 474.2427, Found 474.2411.

**Figure S14** Synthetic schemes for **Biotin-Cara-3**

Reagents and conditions: a) 5-hexynoic acid, HATU, Et_3_N, THF, 20^o^C, 2 h.

To a solution of 5-hexynoic acid (67 mg, 0.6 mmol) in anhydrous THF (2.5 mL) were added HATU (228 mg, 0.6 mmol) and DIPEA (228 mg, 0.6 mmol), then carabrol (100 mg, 0.4 mmol) was added. The reaction mixture was stirred at room temperature for 2 h until completion of the reaction as indicated by TLC, then it was diluted with water and extracted with EA. The combined organic phase was washed with brine, and dried over anhydrous Na_2_SO_4_. The solvent was evaporated to give crude product, which was purified by silica gel column chromatography (PE/ EA = 15:1 to 8:1) to give target compound **Biotin-Cara-3** as a colorless liquid(98 mg, 71%).^1^H NMR (500 MHz, CDCl_3_) *δ* (ppm): 6.26 (d, *J* = 2.1 Hz, 1H), 5.56 (s, 1H), 4.95-4.92 (m, 1H), 4.83-4.78 (m, 1H),3.17 (q, *J* = 8.8 Hz, 1H), 2.44 (t, *J* = 7.4 Hz, 2H), 2.40-2.32 (m, 2H), 2.28 (t, J = 6.6 Hz, 2H), 1.99 (s, 1H), 1.89-1.83 (m, 2H), 1.75-1.67 (m, 1H), 1.61-1.56 (m, 1H), 1.39-1.30 (m, 2H), 1.23 (d, J = 6.2 Hz, 3H), 1.08 (s, 3H), 1.00-0.91 (m, 1H), 0.48-0.44 (m, 1H), 0.38-0.34 (m, 1H). 13C NMR (126 MHz, CDCl3) δ 172.66, 170.49, 139.06, 122.51, 83.29, 75.66, 70.74, 69.08, 37.81, 37.39, 36.00, 34.76, 33.29, 30.81, 24.99, 23.72, 22.90, 19.95, 18.24, 17.87, 17.10. HRMS (ESI): m/z [M+Na]^+^ Calcd for C_21_H_28_NaO_4_ 367.1885, Found 367.1925.

**Figure S15** Synthetic schemes for **Negative**

Reagents and conditions: a) NaBH_4_, methanol, -10^o^C; b) i) 1,1'-carbonyl-di(1,2,4-triazole), 40^o^C, 1.5 h, ii) boc-1-amino-3,6-dioxa-8-octanediamine, CH_2_Cl_2_ 20^o^C, 6.5 h; c) CF_3_COOH, CH_2_Cl_2_ 20^o^C, 2 h; d) (+)-biotin-*N*-hydroxysuccimide ester, Et_3_N, DMF, 20^o^C.

**Synthesis of** **Intermediate** **7**

Carabrol (200 mg, 0.8 mmol) was dissolved in MeOH (4 mL) at -10^o^C, NaBH_4_ (76 mg, 2 mmol) was added and stirred at -10^o^C for 10 min. Then the mixture was warmed to room temperature and stirred for 20 min until completion of the reaction as indicated by TLC, then it was quenched by 5% acetic acid (10 mL) and extracted with EA, the combined organic phase was washed with brine, and dried over anhydrous Na_2_SO_4_. The solvent was evaporated to give crude product, which was purified by silica gel column chromatography (PE/ EA = 5:1 to 2:1) to give intermediates **7** as a colorless liquid (157 mg, 78%). ^1^H NMR (500 MHz, CDCl_3_) *δ* (ppm): 4.77-4.73 (m, 1H), 3.84-3.78 (m, 1H), 2.85-2.78 (m, 1H), 2.51-2.46 (m, 1H), 2.44-2.40 (m, 1H),1.99-1.94 (m, 1H), 1.59-1.48 (m, 2H), 1.40-1.31 (m, 2H), 1.20 (d, *J* = 6.2 Hz, 3H), 1.14 (d, *J* = 7.4 Hz, 3H), 1.06 (s, 3H), 1.00-0.95 (m, 1H), 0.67-0.60 (m, 1H), 0.43-0.39 (m, 1H), 0.28-0.24 (m, 1H).

**Synthesis of** **Intermediate** **8**

The title compound was prepared from intermediate **7** according to the synthesis procedure of intermediate **1**. The reaction crude product was purified by silica gel chromatography to give intermediate **8** (132 mg, 65%) as a colorless liquid.

**Synthesis of Intermediate** **9**

The title compound was prepared from intermediate **8** according to the synthesis procedure of intermediate **2**. The reaction mixture was evaporated under a vacuum to give intermediate **9** as a yellow liquid (81 mg, 84%), which was used in the next step without further purification.

**Negative**

The title compound was prepared from intermediate **9** according to the synthesis procedure of **Biotin-Cara**. The reaction crude product was purified by silica gel chromatography to give **Negative** (34 mg, 43%) as a white solid. ^1^H NMR (500 MHz, CDCl_3_) *δ* (ppm): 6.50 (s, 1H), 6.08 (s, 1H), 5.26 (s, 1H), 5.18 (s, 1H), 4.83-4.81 (m, 1H), 4.78-4.75 (m, 1H), 4.54-4.52 (m, 1H), 4.36-4.33 (m, 1H), 3.64 (s, 4H), 3.59 (t, *J* = 5.0 Hz, 4H), 3.49-3.46 (m, 2H), 3.40-3.38 (m, 2H), 3.20-3.16 (m, 1H), 2.95-2,92 (m, 1H), 2.87-2.81 (m, 2H), 2.77-2.74 (m, 1H), 2.53-2.48 (m, 1H), 2.46-2.42 (m, 1H), 2.27-2.24 (m, 2H), 2.01-1.96 (m, 1H), 1.78-1.66 (m, 6H), 1.50-1.44 (m, 2H), 1.36-1.40 (m, 2H), 1.23 (d, *J* = 6.2 Hz, 3H), 1.16 (d, *J* = 7.4 Hz, 3H), 1.06 (s, 3H), 1.02-0.97 (m, 1H), 0.69-0.62 (m, 1H), 0.44-0.41 (m, 1H). 0.30-0.26 (m, 1H). ^13^C NMR (126 MHz, CDCl3) *δ* (ppm):179.65, 173.39, 164.04, 156.54, 71.32, 70.24, 70.10, 69.98, 61.81, 60.21, 55.62, 40.68, 40.51, 39.12, 38.32, 37.59, 37.19, 36.30, 35.97, 34.45, 28.23, 28.11, 25.62, 25.18, 23.97, 23.04, 20.35, 19.15, 15.48, 10.53. HRMS (ESI): m/z [M-H]^-^ Calcd for C_32_H_51_N_4_O_8_S 651.3428, Found 651.3460.

**Figure S16** Synthetic schemes for **PR-Cara**

Reagents and conditions: a) *tert*-butyl-3-aminopropanoate, Et_3_N, DMSO, 120^o^C, 4 h; b) CF_3_COOH, CH_2_Cl_2_ 20^o^C, 2 h; c) **2**, HATU, DIPEA, CH_2_Cl_2_, 20^o^C, 5 h.

**Synthesis of intermediate 11**

To a solution of **10** (500 mg, 1.81 mmol) in DMSO (10 mL) was added *tert*-butyl-3-aminopropanoate (289 mg, 1.99 mmol) and TEA (406mg, 3.98 mmol), The mixture was stirred at 120^o^C for 4 h until completion of the reaction as indicated by TLC, then it was diluted with water and extracted with EA. The combined organic phase was washed with brine, and dried over anhydrous Na_2_SO_4_. The solvent was evaporated to give crude product, which was purified by silica gel column chromatography (CH_2_Cl_2_/ MeOH = 150:1 to 50:1) to give intermediate **11** as a green solid (300 mg, 41%).^1^H NMR (500 MHz, CDCl_3_) *δ* (ppm): 8.12 (s, 1H), 7.53 (t, *J*=7.8 Hz, 1H), 7.13 (d, *J*=7.1 Hz, 1H), 6.95 (d, *J*= 8.5 Hz, 1H), 6.48 (t, *J*= 5.6 Hz, 1H), 4.94 (dd, *J*= 12.2 Hz, 5.2 Hz, 1H), 3.59 (q, *J*= 6.4 Hz, 2H), 2.92-2.89 (m, 1H), 2.84-2.72 (m, 2H), 2.60 (t, *J*= 6.6 Hz, 2H), 2.16-2.12 (m, 1H), 1.49 (s, 9H).

**Synthesis of Intermediate** **12**

To a solution of **11** (250 mg，0.62 mmol) in CH_2_Cl_2_ (3 mL) was added trifluoroacetic acid (2 mL), The mixture was stirred at room temperature for 2 h until completion of the reaction as indicated by TLC, then it was evaporated under a vacuum to give intermediate **12** as a green liquid (201 mg, 94%), which was used in the next step without further purification.

**PR-Cara**

To a solution of **12** (100 mg, 0.29 mmol) in DMSO were added HATU (121.22 mg, 0.32 mmol) and DIPEA (129 mg, 1.02 mmol), then intermediate **2** was added. The mixture was stirred at room temperature for 5 h, Completion of the reaction was confirmed by TLC, the reaction was diluted with water and extracted with EA, the combined organic phase was washed with brine, and dried over anhydrous Na_2_SO_4_. The solvent was evaporated to give crude product, which was purified by silica gel column chromatography (PE/ EA = 5:1 to 2:1) to give **PR-Cara** as a green solid (65 mg, 30%).^1^H NMR (500 MHz, CDCl_3_) *δ* (ppm): 0.70 (s, 1H), 7.50 (t, *J* = 6.6 Hz,1H), 7.10 (d, *J* = 7.1 Hz, 1H), 6.97 (d, *J* = 8.4 Hz, 1H), 6.38 (s,1H),6.23 (d, *J* = 2.9 Hz, 1H), 5.54 (d, *J* = 2.5 Hz, 1H), 5.14 (s,1H), 4.93-4.89 (m, 1H), 4.80-4.75 (m, 2H), 3.65 (t, *J* = 6.5 Hz, 2H), 3.58-3.51 (m, 6H), 3.46 (m, 2H), 3.35 (m, 2H), 3.17-3.11 (m, 1H), 2.90-2.69 (m, 4H), 2.55-2.54 (m, 1H), 2.36-2.28 (m, 2H), 2.13-2.10 (m, 1H), 2.04-1.99 (m, 1H),1.67-1.62 (m, 1H), 1.55 (m, 1H), 1.34-1.30 (m,2H), 1.26-1.25 (m, 3H), 1.21-1.20 (m, 2H),1.05 (s, 3H), 0.87-0.86 (m, 2H), 0.45-0.41 (m, 1H), 0.34-0.30 (m, 1H). ^13^C NMR (126 MHz, CDCl3) *δ* (ppm): 171.47, 170.75, 170.61, 169.33, 167.61, 156.55, 146.59, 139.06, 136.18, 132.49, 122.60, 116.77, 115.72, 111.70, 110.25, 75.75, 71.37, 70.12, 69.83, 50.76, 48.89, 40.63, 39.29, 38.97, 37.77, 37.35, 36.22, 36.00, 34.71, 31.44, 30.78, 29.69, 24.44, 22.88, 22.75, 20.31, 18.24, 17.05. HRMS (ESI): m/z [M-H]^-^ Calcd for C_38_H_48_N_5_O_1_ 750.3350, Found 750.3355.

**Figure S17** Synthetic schemes for **CA-1**~**CA-5**

Reagents and conditions: a) acyl chloride, DIPEA, CH_2_Cl_2_, 3 h.

To a solution of carabrol (100 mg, 0.4 mmol) in CH_2_Cl_2_ (2 mL) were added acyl chloride (0.6 mmol) and DIPEA (153 mg,1.2 mmol). The mixture was stirred at room temperature for 3 h until completion of the reaction as indicated by TLC, then it was diluted with water and extracted with EA. The combined organic phase was washed with brine, and dried over anhydrous Na_2_SO_4_. The solvent was evaporated to give crude product, which was purified by silica gel column chromatography (PE/ EA = 10:1 to 5:1) to give target compounds **CA-1**~**CA-5**.

**CA-1**：white solid, 80% yield. ^1^H NMR (500 MHz, CDCl_3_) *δ* (ppm): 8.01 (d, *J* = 8.8 Hz, 2H), 6.93 (d, *J* = 8.8 Hz, 2H), 6.25 (d, *J* = 2.8 Hz, 1H), 5.55 (d, *J* = 2.4 Hz, 1H), 5.19-5.12 (m,1H), 4.82-4.77 (m,1H), 3.88 (s, 3H), 3.19-3.13 (m, 1H),2.38-2.31 (m, 1H), 1.89-1.82 (m, 1H), 1.75-1.68 (m, 1H), 1.44-1.39 (m, 2H), 1.35 (d,  *J* = 6.2 Hz, 3H), 1.08 (s, 3H), 1.00-0.88 (m, 2H), 0.51-0.47 (m, 1H), 0.38-0.34 (m, 1H). ^13^C NMR (125 MHz, CDCl_3_) *δ* (ppm): ^13^C NMR (126 MHz, CDCl3) *δ* (ppm): 170.51, 165.95, 163.27, 139.07, 131.49, 123.25, 122.48, 113.56, 75.68, 71.00, 55.44, 37.82, 37.42, 36.19, 34.85, 30.84, 25.10, 22.92, 20.14, 18.25, 17.14. HRMS (ESI): m/z [M+H]^+^ Calcd for C_23_H_29_O_5_ 385.2015, Found 385.2009.

**CA-2**: white solid, 75% yield. ^1^H NMR (500 MHz, CDCl_3_) *δ* (ppm): 8.06 (dd, *J* = 8.9, 5.5 Hz, 2H), 7.13 (t, *J* = 8.7 Hz, 2H), 6.25 (d, *J* = 2.8 Hz, 1H), 5.56 (d, *J* = 2.4 Hz, 1H), 5.20-5.14 (m, 1H), 4.83-4.77 (m, 1H), 3.20-3.14 (m, 1H), 2.39-2.32 (m, 2H), 1.90-1.83 (m, 1H), 1.76-1.69 (m, 1H), 1.44-1.39 (m, 1H), 1.36 (d, *J* = 6.3 Hz, 3H), 1.08 (s, 3H), 1.00-0.90 (m, 2H), 0.51-0.47 (m, 1H), 0.39-0.35 (m, 1H). ^13^C NMR (126 MHz, CDCl3) *δ* (ppm): 170.48, 166.70, 165.21, 164.68, 139.05, 132.04, 131.96, 127.03, 122.51, 115.53, 115.35, 75.64, 71.61, 37.79, 37.39, 36.13, 34.75, 30.81, 25.06, 22.92, 20.05, 18.25, 17.13. HRMS (ESI): m/z [M+H]^+^ Calcd for C_22_H_26_FO_4_ 373.1815, Found 373.1813.

**CA-3**: colorless liquid, 71% yield. ^1^H NMR (500 MHz, CDCl_3_) *δ* (ppm): 7.75 (d, *J* = 7.6 Hz, 1H), 7.44 (t, *J* = 7.9 Hz, 1H), 6.98 (t, *J* = 8.1 Hz, 2H), 6.25 (s, 1H), 5.56 (s, 1H), 5.22-5.16 (m, 1H), 4.82-4.77 (m, 1H), 4.13 (q, *J* = 7.0, 6.4 Hz, 2H), 3.19-3.14 (m, 1H), 2.38-2.31 (m, 2H), 1.87-1.81 (m, 1H),1.74-1.67 (m, 1H), 1.49-1.42 (m, 1H), 1.36 (d, *J* = 6.2 Hz, 3H), 1.09 (s, 3H), 1.00-0.90 (m, 2H),0.52-0.48 (m, 1H), 0.39-0.34 (m, 1H). ^13^C NMR (126 MHz, CDCl3) *δ* (ppm): 170.56, 166.36, 158.25, 139.06, 133.05, 131.22, 122.53, 121.31, 120.03, 113.13, 75.73, 71.17, 64.43, 37.82, 37.42, 36.15, 34.89, 30.83, 25.07, 22.85, 20.09, 18.26, 17.13, 14.81. HRMS (ESI): m/z [M+H]^+^ Calcd for C_24_H_31_O_5_ 399.2171, Found 399.2171.

**CA-4**: colorless liquid, 82% yield. ^1^H NMR (500 MHz, CDCl_3_) *δ* (ppm): 6.26 (d, *J* = 2.9 Hz, 1H), 5.57 (d, *J* = 2.4 Hz, 1H), 4.95-4.88 (m, 1H), 4.83-4.78 (m, 1H), 3.21-3.14 (m, 1H), 2.4.-2.32 (m, 2H), 2.05 (s, 3H), 1.73-1.68 (m, 1H), 1.62-1.56 (m, 1H), 1.37-1.32 (m, 2H), 1.24 (d, *J* = 6.3 Hz, 3H), 1.09 (s, 3H), 1.21-0.91 (m, 2H), 0.48-0.44 (m, 1H), 0.38-0.34 (m, 1H). ^13^C NMR (126 MHz, CDCl3) *δ* (ppm): 170.76, 170.54, 139.42, 122.93, 75.69, 70.73, 37.75, 37.35, 35.92, 34.70, 30.77, 24.92, 22.83, 21.35, 19.89, 18.19, 17.05. HRMS (ESI): m/z [M+H]^+^ Calcd for C_17_H_25_O_4_ 293.1753, Found 293.1744.

**CA-5**: yellow liquid, 80% yield. ^1^H NMR (500 MHz, CDCl_3_) *δ* (ppm): 6.40 (dd, *J* = 17.3, 1.5 Hz, 1H), 6.26 (d, *J* = 2.8 Hz, 1H), 6.12 (dd, *J* = 17.3, 10.4 Hz, 1H), 5.85 – 5.81 (m, 1H), 5.57 (d, *J* = 2.5 Hz, 1H), 5.04-4.98 (m, 1H), 4.83-4.77 (m, 1H), 3.19-3.15 (m, 2H), 1.80-1.72 (m, 1H), 1.67-1.63 (m, 1H), 1.38-1.33 (m, 1H), 1.28 (d, *J* = 6.2 Hz, 3H), 1.08 (s, 3H), 1.00-0.89 (m, 2H),0.49-0.45 (m, 1H),0.38-0.34 (m, 1H). ^13^C NMR (126 MHz, CDCl3) *δ* (ppm): 170.53, 165.85, 139.04, 130.34, 128.97, 122.53, 75.69, 70.93, 37.78, 37.38, 35.96, 34.72, 30.80, 24.96, 22.85, 19.94, 18.22, 17.09. HRMS (ESI): m/z [M+H]^+^ Calcd for C_18_H_25_O_4_ 305.1753, Found 305.1745.

**Figure S18** Synthetic schemes for **CA-6**~**CA-19**

Reagents and conditions: a) 1,1'-Carbonyl-di(1,2,4-triazole), amine, amine, CH_2_Cl_2_ 20^o^C, 6 h.

To a solution of Carabrol (100 mg, 0.4mmol) in CH_2_Cl_2_ (2.5 mL) was added 1,1'-Carbonyl-di(1,2,4-triazole) (98.4 mg, 0.6 mmol). The mixture was warmed to 40^o^C and stirred at 40^o^C for 1.5 h, then it was cooled to room temperature and amino (135 mg, 0.72 mmol) was added. The mixture was stirred at room temperature for 6 h until completion of the reaction as indicated by TLC, the solution was diluted with water and extracted with EA. The combined organic phase was washed with brine, and dried over anhydrous Na_2_SO_4_. The solvent was evaporated to give crude product, which was purified by silica gel column chromatography (PE/ EA = 8:1 to 2:1) to give target compounds **CA-6**~**CA-19**.

**CA-6**: colorless liquid, 69% yield.^1^H NMR (500 MHz, CDCl_3_) *δ* (ppm): 6.25 (d, *J* = 2.9 Hz, 1H), 5.56 (d, *J* = 2.4 Hz, 1H), 4.82-4.77 (m, 2H), 4.56 (s, 1H), 3.20-3.13 (m, 1H), 2.80 (d, *J* = 4.9 Hz, 3H), 2.39-2.31 (m, 1H), 1.67-1.65 (m, 1H), 1.59-1.53 (m, 1H), 1.37-1.30 (m, 2H), 1.22 (d, *J* = 6.3 Hz, 3H), 1.08 (s, 3H), 1.00-0.90 (m, 2H), 0.48-0.44 (m, 1H), 0.37-0.33 (m, 1H). ^13^C NMR (126 MHz, CDCl3) *δ* (ppm): 170.52, 157.02, 139.10, 122.48, 75.71, 71.13, 37.82, 37.41, 36.30, 34.85, 30.83, 27.45, 25.04, 22.91, 20.34, 18.25, 17.08. HRMS (ESI): m/z [M+H]^+^ Calcd for C_17_H_26_NO_4_ 308.1862, Found 308.1856.

**CA-7**: yellow solid, 72% yield. ^1^H NMR (500 MHz, CDCl_3_) *δ* (ppm): 6.23 (d, *J* = 2.8 Hz, 1H), 5.54 (d, *J* = 2.4, 1H), 4.81-4.75 (m, 1H), 3.18-3.12 (m, 1H), 2.38-2.29 (m, 1H), 1.74-1.67 (m, 1H), 1.60-1.53 (m, 1H), 1.36-1.31 (m, 2H), 1.22 (d, *J* = 6.3 Hz), 1.06 (s, 3H), 0.98-0.88 (m, 2H), 0.47-0.43 (m, 1H), 0.36-0.32 (m, 1H).^13^C NMR (126 MHz, CDCl3) *δ* (ppm): 170.55, 156.46, 139.07, 122.50, 75.73, 71.55, 37.82, 37.42, 36.34, 36.28, 35.80, 34.89, 30.83, 25.05, 22.88, 20.42, 18.21, 17.07. HRMS (ESI): m/z [M+H]^+^ Calcd for C_18_H_28_NO_4_ 322.2018, Found 322.2013.

**CA-8**: colorless liquid, 71% yield. ^1^H NMR (500 MHz, CDCl_3_) *δ* (ppm): 6.23 (d, *J* = 2.8 Hz, 1H), 5.55 (d, *J* = 2.5 Hz, 1H), 4.81-4.76 (m, 1H), 4.61 (s, 1H), 3.22-3.19 (m, 2H), 3.17-3.14 (m, 1H), 2.38-2.29 (m, 2H), 1.66 (m, 1H), 1.55 (m, 1H), 1.36-1.30 (m, 2H), 1.20 (d, *J* = 6.0 Hz, 3H), 1.13 (m, *J* = 7.3 Hz, 3H), 1.07 (s, 1H), 0.99-0.89 (m, 1H), 0.46-0.43 (m, 1H), 0.36-0.32 (m, 1H). ^13^C NMR (126 MHz, CDCl3) *δ* (ppm): 170.52, 156.28, 139.10, 122.47, 75.71, 70.92, 37.81, 37.41, 36.31, 35.73, 34.86, 30.83, 25.02, 22.89, 20.31, 18.23, 17.07, 15.29. [M+H]^+^ Calcd for C_18_H_28_NO_4_ 322.2018, Found 322.2014.

**CA-9**: yellow liquid, 72% yield. ^1^H NMR (500 MHz, CDCl_3_) *δ* (ppm): 6.23 (d, *J* = 2.8 Hz, 1H), 5.55 (d, *J* = 2.5 Hz, 1H), 4.81-4.76 (m, 1H), 4.61 (s, 1H), 3.22-3.19 (m, 2H), 3.17-3.14 (m, 1H), 2.38-2.29 (m, 2H), 1.66 (m, 1H), 1.55 (m, 1H), 1.36-1.30 (m, 2H), 1.20 (d, *J* = 6.0 Hz, 3H), 1.13 (m, *J* = 7.3 Hz, 3H), 1.07 (s, 1H), 0.99-0.89 (m, 1H), 0.46-0.43 (m, 1H), 0.36-0.32 (m, 1H). ^13^C NMR (126 MHz, CDCl3) *δ* (ppm): 170.52, 156.28, 139.10, 122.47, 75.71, 70.92, 37.81, 37.41, 36.31, 35.73, 34.86, 30.83, 25.02, 22.89, 20.31, 18.23, 17.07, 15.29. [M+H]^+^ Calcd for C_18_H_28_NO_4_ 322.2018, Found 322.2014.

**CA-10**: colorless liquid, 68% yield. ^1^H NMR (500 MHz, CDCl_3_) *δ* (ppm): 6.26 (d, J = 2.8 Hz, 1H), 5.57 (d, J = 2.4 Hz, 1H), 4.83-4.78 (m, 1H), 4.61 (s, 1H), 3.20-3.13 (m, 3H), 2.40-2.31 (m, 2H), 1.73-1.65 (m, 1H), 1.55-1.51 (m, 2H), 1.38-1.33 (m, 2H), 1.23 (d, *J* = 6.1 Hz, 3H), 1.08 (s, 3H), 1.01-0.96 (m, 2H), 0.94(t, *J* = 7.4 Hz, 4H), 0.48-0.45 (m, 1H), 0.38-0.34 (m, 1H). ^13^C NMR (126 MHz, CDCl3) *δ* (ppm): 170.60, 156.46, 139.06, 122.54, 75.75, 70.88, 42.60, 37.78, 37.38, 36.29, 34.83, 30.81, 25.00, 22.84, 20.31, 18.21, 17.04, 11.22. [M+H]^+^ Calcd for C_19_H_30_NO_4_ 336.2175, Found 350.2173.

**CA-11**: yellow liquid, 65% yield. ^1^H NMR (500 MHz, CDCl_3_) *δ* (ppm): 6.24 (d, *J* = 2.6 Hz, 1H), 5.56 (d, *J* =1.8 Hz, 1H), 4.82-4.76 (m, 2H), 3.19-3.15 (m, 5H), 2.38-2.30 (m, 2H), 1.72-1.68 (m, 1H), 1.61-1.50 (m, 5H), 1.35-1.29 (m, 2H), 1.23-1.21 (m, 3H), 1.07-1.06 (m, 3H), 0.99-0.92 (m, 2H), 0.90-0.87 (m, 6H), 0.47-0.44 (m, 1H), 0.36-0.32 (m, 1H). ^13^C NMR (126 MHz, CDCl3) *δ* (ppm): 170.52, 156.18, 139.10, 122.46, 75.71, 71.10, 49.06, 48.48, 37.85, 37.43, 36.40, 34.94, 30.86, 25.07, 22.92, 21.94, 21.38, 20.32, 18.22, 17.08, 11.27. [M+H]^+^ Calcd for C_22_H_36_NO_4_ 378.2644, Found 378.2659.

**CA-12**: yellow liquid, 55% yield. ^1^H NMR (500 MHz, CDCl_3_) *δ* (ppm): 6.25 (s, 1H), 5.56 (s, 1H), 4.84-4.77 (m, 3H), 3.20-3.15 (m, 1H), 2.59 (s, 1H), 2.19-2.31 (m, 2H), 1.68 (s, 1H), 1.57 (s, 1H), 1.33-1.27 (m, 2H), 1.23 (d, *J* = 6.1 Hz, 3H), 1.08 (s, 3H), 1.00-0.91 (m, 2H), 0.73 (s, 1H), 0.72 (s, 1H), 0.52 (s, 2H), 0.48-0.44 (m, 1H), 0.37-0.33 (m, 1H). ^13^C NMR (126 MHz, CDCl3) *δ* (ppm): 170.60, 157.13, 139.06, 122.55, 75.75, 71.15, 37.79, 37.38, 36.26, 34.81, 30.81, 24.98, 23.03, 22.86, 20.27, 18.23, 17.06, 6.80. [M+H]^+^ Calcd for C_19_H_28_NO_4_ 334.2018, Found 334.2033.

**CA-13**: yellow liquid, 61% yield. ^1^H NMR (500 MHz, CDCl_3_) *δ* (ppm): 6.25 (d, *J* = 2.8 Hz, 1H), 5.56 (d, *J* = 2.4 Hz, 1H), 4.84-4.77 (m, 2H), 3.39 (t, *J* = 5.5 Hz, 2H ), 3.35-3.32 (m, 2H), 3.18-3.14 (m, 1H), 2.39-2.31 (m, 2H), 1.87 (s, 4H), 1.76-1.70 (m, 1H), 1.61-1.55 (m, 1H), 1.38-1.34 (m, 2H), 1.24 (d, *J* = 6.2 Hz, 3H), 1.08 (s, 3H), 1.00-0.90 (m, 2H), 0.49-0.45 (m, 1H), 0.37-0.33 (m, 1H).^13^C NMR (126 MHz, CDCl3) *δ* (ppm): 170.55, 155.00, 139.09, 122.49, 75.74, 71.07, 46.02, 45.68, 37.84, 37.44, 36.41, 34.94, 30.85, 25.72, 25.06, 24.95, 22.90, 20.50, 18.23, 17.08. [M+H]^+^ Calcd for C_20_H_30_NO_4_ 348.2175, Found 348.2169.

**CA-14**: yellow liquid, 72% yield. ^1^H NMR (500 MHz, CDCl_3_) *δ* (ppm): 6.26 (d, *J* = 2.8 Hz, 1H), 5.57 (d, *J* = 2.4 Hz, 1H), 4.87-4.83 (m, 1H), 4.81-4.78 (m, 1H), 3.67 (s, 4H), 3.47 (s, 4H), 3.19-3.15 (m, 1H), 2.40-2.34 (m, 2H), 1.75-1.71 (m, 1H), 1.60-1.56 (m, 1H), 1.37-1.32 (m, 2H), 1.25 (d, *J* = 6.2 Hz, 3H), 1.09 (s, 3H), 1.00-0.93 (m, 2H), 0.48-0.45 (m, 1H), 0.38-0.34 (m, 1H). ^13^C NMR (126 MHz, CDCl3) *δ* (ppm): 170.50, 153.47, 140.81, 122.50, 74.92, 70.88, 68.52, 43.99, 37.77, 37.37, 36.27, 34.76, 31.42, 30.79, 30.17, 24.98, 22.87, 20.27, 18.22, 17.06. [M+H]^+^ Calcd for C_20_H_30_NO_5_ 364.2124, Found 364.2113.

**CA-15**: yellow liquid, 65% yield. ^1^H NMR (500 MHz, CDCl_3_) *δ* (ppm): 6.24 (d, *J* = 2.8 Hz, 1H), 5.56 (s, 1H), 4.84-4.76 (m, 2H), 3.49 (s, 4H), 3.19-3.13 (m, 1H), 2.39-2.36 (m, 4H), 2.36-2.33 (m, 2H), 2.31 (s, 1H), 1.75-1.68 (m,1H), 1.61-1.54 (m,1H), 1.38-1.29 (m, 2H), 1.23 (d, *J* = 6.2 Hz, 3H), 1.07 (s, 3H), 0.99-0.89 (m, 2H), 0.47-0.43 (m, 1H), 0.36-0.32 (m, 1H). ^13^C NMR (126 MHz, CDCl3) *δ* (ppm): 170.57, 155.20, 139.04, 122.56, 75.71, 71.78, 54.74, 50.75, 46.16, 43.52, 37.81, 37.40, 36.30, 34.84, 30.83, 25.01, 22.88, 20.32, 18.24, 17.08. [M+H]^+^ Calcd for C_21_H_33_N_2_O_4_ 377.2440, Found 377.2450.

**CA-16**: colorless liquid, 54% yield. ^1^H NMR (500 MHz, CDCl_3_) *δ* (ppm): 6.25 (d, *J* = 2.9 Hz, 1H), 5.57 (s, 1H), 5.24 (s, 1H), 4.82-4.77 (m, 2H), 3.29 (d, *J* = 6.0 Hz, 2H), 3.19-3.14 (m, 1H), 2.48 (t, *J* = 6.0 Hz, 2H),2.38-2.31 (m, 2H), 2.29 (s, 6H), 1.73-1.68 (m, 1H),1.60-1.55 (m, 1H), 1.38-1.30 (m, 2H), 1.22 (d, *J* = 6.2 Hz, 3H), 1.08 (s, 3H),1.00-0.90 (m, 2H), 0.46-0.44 (m, 1H), 0.37-0.34 (m, 1H). ^13^C NMR (126 MHz, CDCl3) *δ* (ppm): 170.61, 156.53, 139.06, 122.55, 75.76, 71.10, 58.28, 50.74, 45.08, 38.08, 37.82, 37.40, 36.28, 34.87, 30.83, 25.01, 22.86, 20.31, 18.23, 17.07. [M+H]^+^ Calcd for C_20_H_33_N_2_O_4_ 365.2440, Found 365.2451.

**CA-17**: yellow liquid, 62% yield. ^1^H NMR (500 MHz, CDCl_3_) *δ* (ppm): 6.26 (d, *J* = 2.8 Hz, 1H), 5.57 (d, *J* = 2.4 Hz, 1H), 4.83-4.77 (m, 2H), 3.40-3.35 (m, 2H),3.19-3.14 (m, 1H), 2.92 (d, *J* = 12.1 Hz, 3H), 2.48-2.43 (m, 2H), 2.38-2.33 (m, 2H), 2.28 (s, 6H), 1.76-1.69 (m, 1H), 1.62-1.56 (m, 1H), 1.39-1.30 (m, 2H), 1.24 (d, *J* = 6.2 Hz, 3H), 1.08 (s, 3H), 1.00-0.90 (m, 2H), 0.47-0.44 (m, 1H), 0.37-0.33 (m, 1H). ^13^C NMR (126 MHz, CDCl3) *δ* (ppm): 169.78, 156.24, 139.15, 121.36, 75.83, 71.72, 57.38, 56.86, 50.79, 46.87, 45.78, 37.91, 37.50, 36.05, 34.97, 30.92, 25.14, 22.62, 20.45, 18.33, 17.17. [M+H]^+^ Calcd for C_21_H_35_N_2_O_4_ 379.2597, Found: 379.2606.

**CA-18**: yellow liquid, 55% yield. ^1^H NMR (500 MHz, CDCl_3_) *δ* (ppm): 6.26 (d, *J* = 2.5 Hz, 1H), 5.57 (d, *J* = 2.2 Hz, 1H), 4.83-4.77 (m, 2H), 3.38-3.19 (m, 4H), 3.19-3.14 (m, 1H), 2.66 (s, 4H), 2.59 (s, 2H), 2.40-2.59 (m, 2H), 1.74-1.68 (m, 1H), 1.64-1.60 (m, 1H), 1.37-1.33 (m, 2H), 1.24 (d, *J* = 6.2 Hz, 3H), 1.14-1.08 (m, 12H), 1.00-0.90 (m, 2H), 0.48-0.44 (m, 1H), 0.37-0.33 (m, 1H). ^13^C NMR (126 MHz, CDCl3) *δ* (ppm): 171.12, 170.52, 139.05, 122.48, 75.69, 71.31, 60.36, 47.60, 47.42, 37.80, 37.39, 36.38, 34.85, 30.81, 25.05, 22.87, 21.02, 20.34, 18.21, 17.06, 14.18, 11.84, 11.49. [M+H]^+^ Calcd for C_24_H_41_N_2_O_4_ 421.3066, Found: 421.3094.

**CA-19**: yellow liquid, 60% yield. ^1^H NMR (500 MHz, CDCl_3_) *δ* (ppm): 7.35-7.34 (m, 2H), 7.31-7.29 (m, 3H), 6.26 (d, *J* = 2.8 Hz, 1H), 5.57 (s, 1H), 4.94 (s, 1H), 4.86 (q, *J* = 6.3 Hz, 1H), 4.83-4.77 (m, 1H), 4.39 (d, *J* = 4.7 Hz, 2H), 3.19-3.14 (m, 1H), 2.39-2.3 (m, 2H), 1.73-1.67 (m, 1H), 1.61-1.55 (m, 1H), 1.38-1.30 (m, 2H), 1.25 (d, *J* = 6.2 Hz, 3H), 1.08 (s, 3H), 1.00-0.90 (m, 2H), 0.48-0.44 (m, 1H),0.41-0.32 (m, 1H). ^13^C NMR (126 MHz, CDCl3) *δ* (ppm): 170.57, 156.41, 139.07, 138.67, 128.67, 127.52, 127.47, 122.54, 75.72, 71.40, 45.00, 37.81, 37.40, 36.29, 34.83, 30.83, 25.03, 22.88, 20.33, 18.25, 17.08. [M+H]^+^ Calcd for C_23_H_30_NO_4_ 384.2175, Found 384.2203.

**Figure S19** Synthetic schemes for **CA-20**~**CA-25**

Reagents and conditions: a) isocyanate, DIPEA, CH_2_Cl_2_, 20^o^C, 5 h.

To a solution of carabrol (100 mg, 0.4mmol) in CH_2_Cl_2_ (2.5 mL) were added isocyanate (0.6 mmol) and DIPEA (102 mg, 0.8 mmol), the mixture was stirred at room temperature for 5 h until completion of the reaction as indicated by TLC, then it was diluted with water and extracted with EA. The combined organic phase was washed with brine, and dried over anhydrous Na_2_SO_4_. The solvent was evaporated to give crude product, which was purified by silica gel column chromatography (PE/ EA = 10:1 to 3:1) to give target compounds **CA-20**~**CA-25**.

**CA-20**: white solid, 67% yield. ^1^H NMR (500 MHz, CDCl_3_) *δ* (ppm): 7.31 (s, 2H), 6.87 (d, *J* = 9.0 Hz, 2H), 6.45 (s, 1H), 6.25 (d, *J* = 2.9 Hz, 1H), 5.55 (d, *J* = 2.4 Hz, 1H), 4.92 (q, *J* = 6.3 Hz, 1H), 4.83-4.77 (m, 1H), 3.81 (s, 3H), 3.19-3.13 (m, 1H), 2.39-2.31 (m, 2H), 1.78-1.72 (m, 1H), 1.67-1.63 (m, 1H), 1.44-1.34 (m, 2H), 1.29 (d, *J* = 6.3 Hz, 3H), 1.09 (s, 3H), 1.02-0.94 (m, 2H), 0.50-0.46 (m, 1H), 0.39-0.35 (m, 1H). ^13^C NMR (126 MHz, CDCl3) *δ* (ppm): 170.58, 155.88, 153.68, 139.07, 131.15, 122.56, 120.42, 114.25, 75.73, 71.71, 55.52, 37.76, 37.36, 36.22, 34.73, 30.80, 25.08, 22.89, 20.31, 18.27, 17.08. [M-H]^-^ Calcd for C_23_H_28_NO_5_ 398.1967, Found 398.1959.

**CA-21**: white solid, 72% yield. ^1^H NMR (500 MHz, CDCl_3_) *δ* (ppm): 7.21 (t, *J* = 8.1 Hz, 1H), 7.15 (s, 1H), 6.87 (d, *J* = 10.6 Hz, 1H), 6.63 (dd, *J* = 8.3, 2.5 Hz, 1H), 6.58 (s, 1H), 6.25 (d, *J* = 2.8 Hz, 1H), 5.55 (d, *J* = 2.4 Hz, 1H), 4.95-4.91 (m, 1H), 4.83-4.78 (m, 1H), 3.82 (s, 3H), 3.20-3.14 (m, 1H), 2.39-2.32 (m, 2H), 1.80-1.73 (m, 1H), 1.67-1.64 (m, 1H), 1.46-1.32 (m, 2H), 1.30 (d, *J* = 6.3 Hz, 3H), 1.09 (s, 3H), 1.02-0.91 (m, 2H), 0.50-0.46 (m, 1H), 0.39-0.35 (m, 1H). ^13^C NMR (126 MHz, CDCl3) *δ* (ppm): 170.52, 160.33, 153.18, 139.30, 139.08, 129.75, 122.52, 110.72, 109.09, 104.28, 75.68, 71.90, 55.28, 37.75, 37.36, 36.19, 34.70, 30.79, 25.09, 22.92, 20.28, 18.28, 17.10. [M-H]^-^ Calcd for C_23_H_28_NO_5_ 398.1967, Found 398.1962.

**Ca-22**: white solid, 65% yield. ^1^H NMR (500 MHz, CDCl_3_) *δ* (ppm): 7.35 (s, 2H), 7.02 (t, *J* = 8.6 Hz, 2H), 6.53 (s, 1H), 6.25 (d, J = 2.9 Hz, 1H), 5.55 (d, J = 2.5 Hz, 1H), 4.92 (q, *J* = 6.3 Hz, 1H), 4.83-4.78 (m, 1H), 3.20-3.15 (m, 1H), 2.39-2.32 (m, 2H), 1.80-1.73 (m, 1H), 1.67-1.63 (m, 1H), 1.43-1.35 (m, 2H), 1.30 (d, *J* = 6.2 Hz, 3H), 1.09 (s, 3H), 1.03-0.93 (m, 2H), 0.50-0.46 (m, 1H), 0.39-0.35 (m, 1H). ^13^C NMR (126 MHz, CDCl3) *δ* (ppm): 170.57, 159.86, 157.93, 153.46, 139.08, 134.05, 122.57, 120.25, 115.75, 115.57, 75.70, 71.99, 37.72, 37.33, 36.18, 34.63, 30.77, 25.07, 22.90, 20.26, 18.29, 17.08. [M-H]^-^ Calcd for C_22_H_25_FNO_4_ 386.1768, Found 368.1753.

**CA-23**: white solid, 62% yield. ^1^H NMR (500 MHz, CDCl_3_) *δ* (ppm): 7.35 (d, *J* = 8.1 Hz, 2H), 7.27 (d, *J* = 8.7 Hz, 2H), 6.68 (s, 1H), 6.24 (d, *J* = 2.8 Hz, 1H), 5.55 (d, *J* = 2.2 Hz, 1H), 4.92 (q, *J* = 6.3 Hz, 1H), 3.19-3.14 (m, 1H), 2.38-2.31 (m, 2H), 1.79-1.72 (m, 1H), 1.67-1.62 (m, 1H), 1.29 (d, *J* = 6.3 Hz, 4H), 1.08 (s, 3H), 1.02-0.92 (m, 2H), 0.49-0.45 (m, 1H), 0.37-0.34 (m, 1H). ^13^C NMR (126 MHz, CDCl3) *δ* (ppm): 170.58, 153.18, 139.07, 136.70, 129.03, 128.25, 122.59, 119.76, 75.71, 72.13, 37.70, 37.31, 36.15, 34.59, 30.75, 25.06, 22.89, 20.25, 18.29, 17.07. [M-H]^-^ Calcd for C_22_H_25_ClNO_4_ 402.1472, Found 402.1474.

**CA-24**: white solid, 75% yield. ^1^H NMR (500 MHz, CDCl_3_) *δ* (ppm): 7.27 (s, 2H), 7.13 (d, *J* = 8.2 Hz, 2H), 6.49 (s, 1H), 6.25 (d, *J* = 2.9 Hz, 1H), 5.55 (d, *J* = 2.5 Hz, 1H), 4.92 (q, *J* = 6.3 Hz, 1H), 4.83-4.78 (m, 1H), 3.20-3.15 (m, 1H), 2.39-2.34 (m, 2H), 2.32 (s, 3H), 1.80-1.74 (m, 1H), 1.67-1.63 (m, 1H), 1.43-1.36 (m, 2H), 1.30 (d, *J* = 6.3 Hz, 3H), 1.09 (s, 3H), 1.02-0.91 (m, 2H), 0.50-0.46 (m, 1H), 0.39-0.36 (m, 1H). ^13^C NMR (126 MHz, CDCl3) *δ* (ppm): 170.56, 153.43, 139.07, 135.43, 132.90, 129.54, 122.54, 118.69, 75.71, 71.73, 37.77, 37.37, 36.22, 34.74, 30.80, 25.09, 22.91, 20.73, 20.30, 18.27, 17.09. [M-H]^-^ Calcd for C_22_H_28_NO_4_ 382.2018, Found 382.2007.

**CA-25**: white solid, 55% yield. ^1^H NMR (500 MHz, CDCl_3_) *δ* (ppm): 7.43 (d, *J* = 8.4 Hz, 2H), 7.17 (d, *J* = 8.5 Hz, 2H), 6.74 (s, 1H), 6.24 (d, *J* = 2.5 Hz, 1H), 5.54 (d, *J* = 2.4 Hz, 1H), 4.93 (q, *J* = 6.3 Hz, 1H), 4.82-4.77 (m, 1H), 3.20-3.13 (m, 1H), 2.39-2.31 (m, 2H), 1.80-1.72 (m, 1H), 1.66-1.60 (m, 1H), 1.45-1.35 (m, 2H), 1.30 (d, *J* = 6.2 Hz, 3H), 1.08 (s, 3H), 1.03-0.92 (m, 2H), 0.49-0.48 (m, 1H), 0.39-0.35 (m, 1H). ^13^C NMR (126 MHz, CDCl3) *δ* (ppm): 170.58, 153.23, 144.60, 139.08, 136.83, 122.59, 121.89, 119.55, 119.49, 75.71, 72.20, 37.69, 37.31, 36.14, 34.57, 30.75, 25.06, 22.90, 20.24, 18.28, 17.07. [M-H]^-^ Calcd for C_23_H_25_F_3_NO_5_ 452.1685, Found 452.1680.

**Figure S20** Synthetic schemes for **CA-26**~**CA-28**

Reagents and conditions: a) i) triphosgene, Et_3_N, CH_2_Cl_2_, 20^o^C, 5 min, ii) amine, 20^o^C, 4 h.

To a solution of triphosgene (71 mg, 0.24 mmol) in CH_2_Cl_2_ (3 mL) were added carabrol (100 mg, 0.4 mmol) and TEA (122 mg, 1.2 mmol), the mixture was stirred at room temperature for 5 minutes. Amino (0.6 mmol) was added and stirred at room temperature for 4 h until completion of the reaction as indicated by TLC, then it was diluted with water and extracted with EA. The combined organic phase was washed with brine, and dried over anhydrous Na_2_SO_4_. The solvent was evaporated to give crude product, which was purified by silica gel column chromatography (CH_2_Cl_2_/ MeOH = 100:1 to 50:1) to give target compounds **CA-26**~**CA-28**.

**CA-26**: yellow solid, 23% yield. ^1^H NMR (500 MHz, CDCl_3_) *δ* (ppm): 7.25 (s, 2H), 6.73 (d, *J* = 8.4 Hz, 2H), 6.39 (s, 1H), 6.25 (d, *J* = 2.8 Hz, 1H), 5.55 (d, *J* = 2.5 Hz, 1H), 4.95-4.87 (m, 1H), 4.83-4.77 (m, 1H), 3.20-3.13 (m, 1H), 2.93 (s, 6H), 2.39-2.32 (m, 2H), 1.79-1.72 (m,1H), 1.66-1.62 (m, 1H), 1.42-1.37 (m, 2H), 1.29 (d, *J* = 6.2 Hz, 3H), 1.09 (s, 3H), 1.01-0.93 (m, 2H), 0.50-0.46 (m, 1H), 0.39-0.35 (m, 1H). ^13^C NMR (126 MHz, CDCl3) *δ* (ppm): 170.58, 153.81, 142.28, 139.07, 136.79, 122.55, 120.66, 113.54, 75.74, 71.53, 41.18, 37.79, 37.39, 36.26, 34.80, 30.82, 25.09, 22.90, 20.33, 18.27, 17.09. [M-H]^-^ Calcd for C_24_H_31_N_2_O_4_, 411.2284, Found 411.2266.

**CA-27**: yield solid, 27% yield. ^1^H NMR (500 MHz, CDCl_3_) *δ* (ppm): 7.30 (s, 2H), 6.91 (d, *J* = 9.0 Hz, 2H), 6.42 (s, 1H), 6.25 (d, *J* = 2.9 Hz, 1H), 5.55 (d, *J* = 2.4 Hz, 1H), 4.92 (q, *J* = 6.4 Hz, 1H), 4.83-4.77 (m,1H), 3.24 (t, *J* = 5.0 Hz, 4H), 3.20-3.13 (m, 1H), 2.71 (s, 4H), 2.45 (s, 3H), 2.39-2.31 (m, 2H), 1.78-1.72 (m, 1H), 1.67-1.61 (m, 1H), 1.38-1.34 (m, 2H), 1.28 (d, *J* = 4.0 Hz, 3H), 1.09 (s, 3H), 1.02-0.94 (m, 2H), 0.50-0.67 (m, 1H), 0.39-0.35 (m, 1H). ^13^C NMR (126 MHz, CDCl3) *δ* (ppm): 170.60, 153.66, 147.27, 139.06, 131.34, 122.57, 120.12, 117.28, 75.74, 71.66, 55.64, 49.24, 45.55, 37.75, 37.36, 36.21, 34.72, 30.79, 25.07, 22.89, 20.30, 18.27, 17.07. [M-H]^-^ Calcd for C_27_H_36_N_3_O_4_, 466.2706, Found 466.2703.

**CA-28**: white solid, 53% yield. ^1^H NMR (500 MHz, CDCl_3_) *δ* (ppm): 8.07 (s, 1H), 7.82 (s, 1H), 6.74 (d, *J* = 8.8 Hz, 1H), 6.45 (s, 1H), 6.25 (d, *J* = 2.9 Hz, 1H), 5.56 (d, *J* = 2.5 Hz, 1H), 4.92 (q, *J* = 6.3 Hz, 1H), 4.83-4.78 (m, 1H), 3.93 (s, 3H), 3.20-3.15 (m, 1H), 2.42-2.32 (m, 2H), 1.80-1.73 (m, 1H), 1.67-1.64 (m, 1H), 1.43-1.35 (m, 2H), 1.30 (d, *J* = 6.2 Hz, 3H), 1.09 (s, 3H), 1.03-0.93 (m, 2H), 0.50-0.46 (m, 1H), 0.39-0.35 (m, 1H). ^13^C NMR (126 MHz, CDCl3) *δ* (ppm): 170.56, 160.70, 153.77, 139.07, 137.47, 131.29, 128.73, 122.58, 110.70, 75.70, 72.19, 53.56, 37.72, 37.33, 36.17, 34.63, 30.77, 25.04, 22.89, 20.24, 18.28, 17.08. [M-H]^-^ Calcd for C_22_H_29_N_2_O_5_ 399.1920, Found 399.1918.

**Figure S21** Synthetic schemes for **CA-29**~ **CA-31**

Reagents and conditions: a) acyl chloride, DIPEA, CH_2_Cl_2_, , 20^o^C, 2 h.

To a solution of **6** (100 mg, 0.29 mmol) in CH_2_Cl_2_ (2 mL) were added acyl chloride (0.44 mmol) and DIPEA (148 mg, 1.16mmol), the reaction mixture was stirred at room temperature for 2 h until completion of the reaction as indicated by TLC, then it was diluted with water and extracted with EA. The combined organic phase was washed with brine, and dried over anhydrous Na_2_SO_4_. The solvent was evaporated to give crude product, which was purified by silica gel column chromatography (PE/ EA = 10:1 to 3:1) to give target compounds **CA-29**~**CA-31**.

**CA-29**: white solid, 64% yield. ^1^H NMR (500 MHz, CDCl_3_) *δ* (ppm): 7.73 (d, *J* = 8.5 Hz, 2H), 6.94 (d, *J* = 8.4 Hz, 2H), 6.25 (d, *J* = 2.8 Hz, 1H), 5.77 (d, *J* = 10.0 Hz, 1H), 5.57 (d, *J* = 2.4 Hz, 1H), 4.82-4.76 (m, 1H), 4.28-4.22 (m, 1H), 3.87 (m, 3H), 3.20-3.14 (m, 1H), 2.42-2.37 (m, 1H), 2.32 (dd, *J* = 13.7, 6.1 Hz, 1H), 1.68-1.65 (m, 2H), 1.52-1.44 (m, 1H), 1.37-1.32 (m, 1H), 1.26 (d, *J* = 6.6 Hz, 3H), 1.08 (s, 3H), 1.01-0.93 (m, 2H), 0.48-0.45 (m, 1H), 0.40-0.37 (m, 1H). ^13^C NMR (126 MHz, CDCl3) *δ* (ppm): 170.60, 166.39, 162.06, 139.08, 128.58, 127.22, 122.57, 113.72, 75.75, 55.42, 45.31, 37.75, 37.38, 37.27, 34.74, 30.83, 25.68, 23.00, 21.44, 18.35, 16.88. [M+H]^+^ Calcd for C_23_H_30_NO_4_, 384.2175, Found 384.2194.

**CA-30**: white solid, 60% yield. ^1^H NMR (500 MHz, CDCl_3_) *δ* (ppm): 7.64 (d, *J* = 8.3 Hz, 2H), 7.23 (d, *J* = 7.9 Hz, 2H), 6.23 (d, *J* = 2.9 Hz, 1H), 5.80 (d, *J* = 8.5 Hz, 1H), 5.55 (d, *J* = 2.5 Hz, 1H), 4.79-4.74 (m, 1H), 4.26-4.21 (m,1H), 3.18-3.11 (m, 1H), 2.39 (s, 3H), 2.37-2.34 (m, 1H), 2.30 (dd, *J* = 13.7, 6.1 Hz, 1H), 1.66-1.62 (m, 2H), 1.50-1.43(m, 1H), 1.34-1.30 (m, 1H), 1.24 (d, *J* = 6.6 Hz, 3H), 1.05 (s, 3H), 0.98-0.89 (m, 2H), 0.46-0.42 (m, 1H), 0.38-0.34 (m, 1H). ^13^C NMR (126 MHz, CDCl3) *δ* (ppm): 170.59, 166.81, 141.74, 139.08, 132.09, 129.21, 126.77, 122.56, 75.74, 45.32, 37.76, 37.38, 37.27, 34.72, 30.82, 25.66, 23.03, 21.42, 18.35, 16.89. [M+H]^+^ Calcd for C_23_H_30_NO_3_ 368.2226, Found 368.2245.

**CA-31**: white solid, 63% yield. ^1^H NMR (500 MHz, CDCl_3_) *δ* (ppm): 7.78 (dd, *J* = 8.7, 5.3 Hz, 2H), 7.08 (t, *J* = 8.6 Hz, 2H), 6.21 (d, *J* = 2.8 Hz, 1H), 6.17 (d, *J* = 8.4 Hz, 1H), 5.55 (d, *J* = 2.4 Hz, 1H), 4.79-4.74 (m, 1H), 4.23-4.18 (m, 1H), 3.17-3.11 (m, 1H), 2.39-2.34 (m, 1H), 2.28 (dd, *J* = 13.7, 6.1 Hz, 1H), 1.65-1.61 (m, 2H), 1.48-1.41 (m, 1H), 1.33-1.29 (m, 1H), 1.23 (d, *J* = 6.6 Hz, 3H), 1.05 (s, 3H), 0.96-0.88 (m, 2H), 0.44-0.41 (m, 1H), 0.37-0.33 (m, 1H). ^13^C NMR (126 MHz, CDCl3) *δ* (ppm): 170.63, 165.91, 165.58, 163.58, 139.07, 131.12, 131.09, 129.23, 129.15, 122.60, 115.56, 115.39, 75.78, 45.57, 37.71, 37.36, 37.10, 34.69, 30.80, 25.69, 23.00, 21.26, 18.33, 16.87. [M+H]^+^ Calcd for C_22_H_27_FNO_3_ 372.1975, Found 372.2002.

**Figure S22** Synthetic schemes for **CA-32**~ **CA-35**

Reagents and conditions: a) acid, HATU, DIPEA, CH_2_Cl_2_, 2 h.

To a solution of Carboxylic acid (0.44 mmol) in CH_2_Cl_2_ (1 mL) were added HATU (165.3 mg, 0.44 mmol) and DIPEA (148 mg, 1.16mmol),and subsequently **6** was added and stirred at room temperature for 2 h until completion of the reaction as indicated by TLC, then it was diluted with water and extracted with EA. The combined organic phase was washed with brine, and dried over anhydrous Na_2_SO_4_. The solvent was evaporated to give crude product, which was purified by silica gel column chromatography (CH_2_Cl_2_/ MeOH = 100:1 to 30:1) to give target compounds **CA-32**~**CA-35**.

**CA-32**: white solid, 58% yield. ^1^H NMR (500 MHz, CDCl_3_) *δ* (ppm): 7.72 (d, *J* = 8.3 Hz, 2H), 7.41 (d, *J* = 7.9 Hz, 2H), 6.24 (d, *J* = 2.9 Hz, 1H), 5.90 (d, *J* = 8.5 Hz, 1H), 5.57 (d, *J* = 2.5 Hz, 1H), 4.81-4.76 (m, 1H), 4.28-4.22 (m, 1H), 3.72 (t, *J* = 4.6 Hz, 4H), 3.54 (s, 2H), 3.20-3.13 (m, 1H), 2.45 (s, 4H), 2.42-2.36 (m,1H), 2.31 (dd, *J* = 13.7, 6.1 Hz, 1H), 1.67-1.62 (m, 2H), 1.52-1.46 (m, 1H), 1.34-1.30 (m, 1H), 1.26 (d, *J* = 6.6 Hz, 3H), 1.07 (s, 3H), 0.98-0.89 (m, 2H), 0.47-0.44 (m, 1H), 0.40-0.36 (m, 1H). ^13^C NMR (126 MHz, CDCl3) *δ* (ppm): 170.55, 166.68, 141.60, 139.09, 133.86, 129.21, 126.80, 122.55, 75.71, 66.97, 62.94, 53.62, 45.39, 37.74, 37.37, 37.27, 34.69, 30.81, 25.68, 23.05, 21.40, 18.37, 16.89. [M-H]^-^ Calcd for C_27_H_35_N_2_O_4_ 451.2597, Found 451.2599.

**CA-33**: white solid, 49% yield. ^1^H NMR (500 MHz, CDCl_3_) *δ* (ppm): 7.68 (d, *J* = 8.9 Hz, 2H), 6.90 (d, *J* = 8.9 Hz, 2H), 6.25 (d, *J* = 2.9 Hz, 1H), 5.75 (d, *J* = 8.5 Hz, 1H), 5.56 (d, *J* = 2.5 Hz, 1H), 4.81-4.75 (m, 1H), 4.28-4.21 (m, 1H), 3.32 (t, *J* = 5.1 Hz, 4H), 3.19-3.12 (m, 1H), 2.59 (t, *J* = 5.0 Hz, 4H), 2.42-2.39 (m, 1H), 2.33-2.29, 2.31 (dd, *J* = 13.7, 6.1 Hz, 1H), 1.66-1.61 (m, 2H), 1.51-1.44 (m, 1H), 1.36-1.28 (m, 1H), 1.24 (d, *J* = 6.6 Hz, 3H), 1.07 (s, 3H), 1.00-0.90 (m, 2H), 0.47-0.44 (m, 1H), 0.39-0.35 (m, 1H). ^13^C NMR (126 MHz, CDCl3) *δ* (ppm): 170.56, 166.45, 153.24, 139.11, 128.15, 124.70, 122.51, 114.35, 75.73, 54.82, 47.90, 46.11, 45.14, 37.77, 37.39, 37.39, 34.77, 30.84, 25.66, 23.03, 21.53, 18.35, 16.89. [M+Cl]^-^ Calcd for C_27_H_37_ClN_3_O_3_ 486.2523, Found 286.2527.

**CA-34**: white solid, 65% yield. ^1^H NMR (500 MHz, CDCl_3_) *δ* (ppm): 6.87 (d, *J* = 8.8 Hz, 1H), 6.25 (q, *J* = 2.6 Hz, 1H), 5.57 (t, *J* = 2.1 Hz, 1H), 4.82-4.76 (m, 1H), 4.06-4.01 (m, 1H), 3.73 (s, 4H), 3.17 (q, *J* = 9.6 Hz, 1H), 3.00 (s, 2H), 2.54 (s, 4H), 2.41-2.36 (m, 1H), 2.34-2.30 (m, 1H), 1.59-1.53 (m, 2H), 1.41-1.35 (m, 1H), 1.28-1.23 (m, 1H), 1.16 (d, *J* = 6.6 Hz, 3H), 1.07 (s, 3H), 1.01-0.93 (m, 2H), 0.46-0.42 (m, 1H), 0.38-0.34 (m, 1H). ^13^C NMR (126 MHz, CDCl3) *δ* (ppm): 170.51, 168.93, 139.06, 122.56, 75.65, 66.94, 62.10, 53.86, 50.84, 44.40, 37.74, 37.35, 37.12, 34.60, 30.78, 25.71, 23.08, 21.32, 18.33, 16.88. [M+H]^+^ Calcd for C_21_H_33_N_2_O_4_ 377.2440, Found 377.2468.

**CA-35**: white solid, 57% yield. ^1^H NMR (500 MHz, CDCl_3_) *δ* (ppm): 6.91 (d, *J* = 9.1 Hz, 1H), 6.23 (d, *J* = 2.8 Hz, 1H), 5.55 (d, *J* = 2.4 Hz, 1H), 4.80-4.74 (m, 1H), 4.06-4.00 (m, 1H), 3.17-3.11 (m, 1H), 2.92 (s, 2H), 2.40-2.34 (m, 1H), 2.32-2.29 (m, 1H), 2.27 (s, 6H), 1.57-1.51 (m, 2H), 1.43-1.38 (m, 1H), 1.26-1.20 (m, 1H), 1.15 (d, *J* = 6.5 Hz, 3H), 1.05 (s, 3H), 0.98-0.89 (m, 2H), 0.44-0.40 (m, 1H), 0.36-0.32 (m, 1H). ^13^C NMR (126 MHz, CDCl3) *δ* (ppm): 170.62, 169.39, 139.05, 122.62, 75.76, 63.07, 45.84, 44.35, 37.78, 37.38, 37.04, 34.71, 30.83, 25.68, 23.04, 21.31, 18.33, 16.88. [M-H]^-^ Calcd for C_19_H_19_N_2_O_3_ 333.2178, Found 333.2192.

**Figure S23** Synthetic schemes for **CA-36**

Reagents and conditions: a) dimethylcarbamoyl chloride, DIPEA, CH_2_Cl_2_, 2 h.

To a solution of **6** (100 mg, 0.29 mmol) in CH_2_Cl_2_ (2 mL) were added dimethylcarbamoyl chloride (47 mg, 0.44 mmol) and DIPEA (148 mg，1.16mmol), the reaction mixture was stirred at room temperature for 3 h until completion of the reaction as indicated by TLC, then it was diluted with water and extracted with EA. The combined organic phase was washed with brine, and dried over anhydrous Na_2_SO_4_. The solvent was evaporated to give crude product, which was purified by silica gel column chromatography (PE/ EA = 10:1 to 2:1) to give target compounds **CA-36** as a white solid (59 mg, 63%).^1^H NMR (500 MHz, CDCl_3_) *δ* (ppm): 6.24 (d, *J* = 2.9 Hz, 1H), 5.56 (d, *J* = 2.5 Hz, 1H), 4.81-4.76 (m, 1H), 4.08 (d, *J* = 8.1 Hz, 1H), 3.91-3.85 (m, 1H), 3.19-3.13 (m, 1H), 2.89 (s, 6H), 2.40-2.34 (m, 1H), 2.33-2.29 (m, 1H), 1.52 (t, *J* = 7.1 Hz, 2H), 1.43-1.37 (m, 1H), 1.32-1.25 (m, 1H), 1.14 (d, *J* = 6.5 Hz, 3H), 1.07 (s, 3H), 0.99-0.90 (m, 2H), 0.46-0.42 (m, 1H), 0.39-0.35 (m, 1H). ^13^C NMR (126 MHz, CDCl3) *δ* (ppm): 170.57, 157.97, 139.12, 122.50, 75.76, 46.25, 37.80, 37.77, 37.42, 36.18, 34.91, 30.86, 25.70, 22.92, 22.05, 18.32, 16.92. [M-H]^-^ Calcd for C_18_H_27_N_2_O_3_, 319.2022, Found 319.2040.

# Spectrum of compound in this paper

^1^H NMR of **carabrone** (CDCl_3_)

^13^C NMR of **carabrone** (CDCl_3_)

HRMS spectrum of **carabrone**


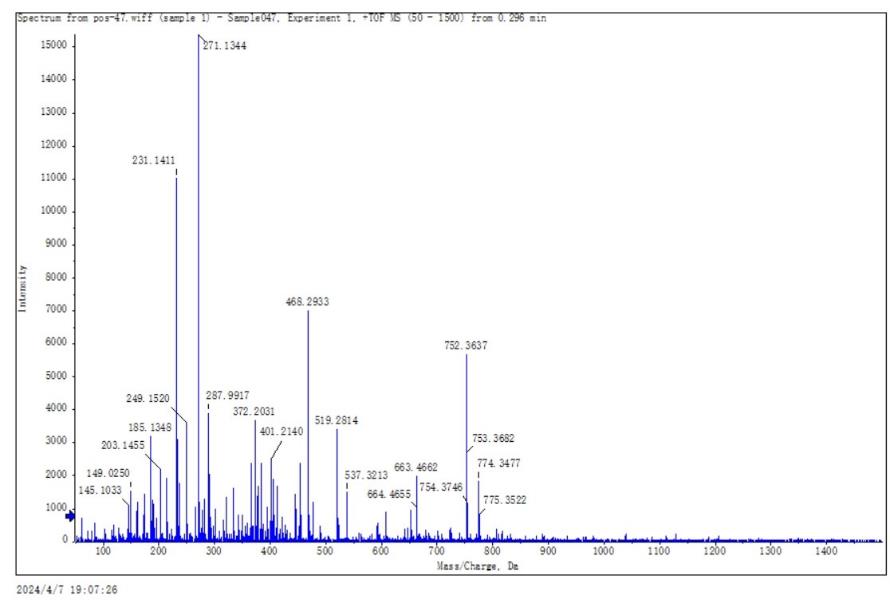


^1^H NMR of **carabrol** (CDCl_3_)

^13^C NMR of **carabrol** (CDCl_3_)

HRMS spectrum of **carabrol**


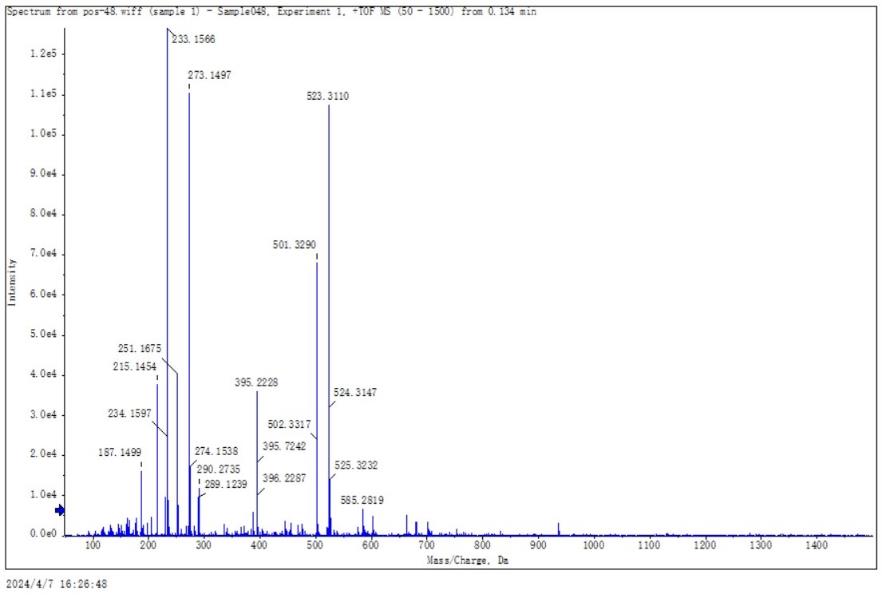


^1^H NMR of **Biotin-Cara** (CDCl_3_)

^13^C NMR of **BIO-1** (CDCl_3_)

HRMS spectrum of **Biotin-Cara**


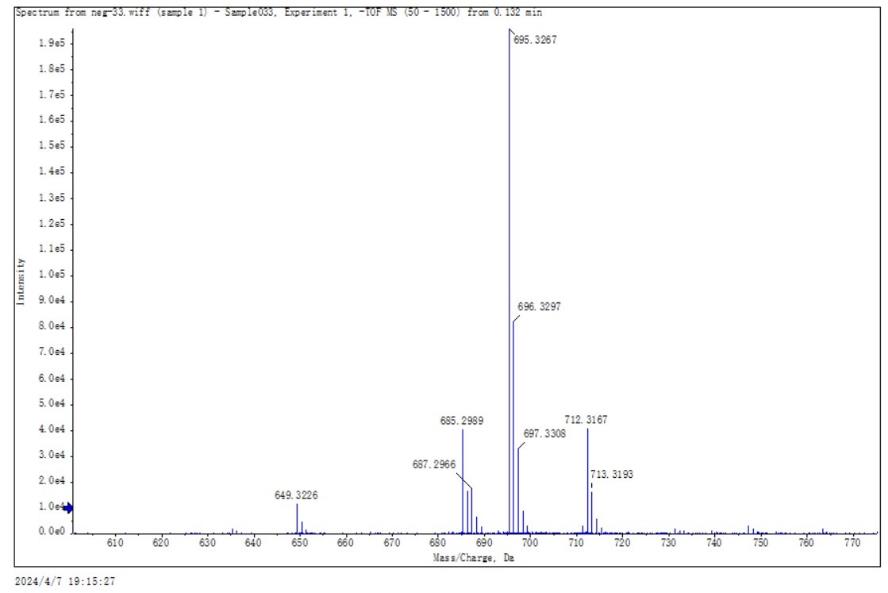


^1^H NMR of **Biotin-Cara-1**(CDCl_3_)

^13^C NMR of **Biotin-Cara-1** (CDCl_3_)

HRMS spectrum of **Biotin-Cara-1**


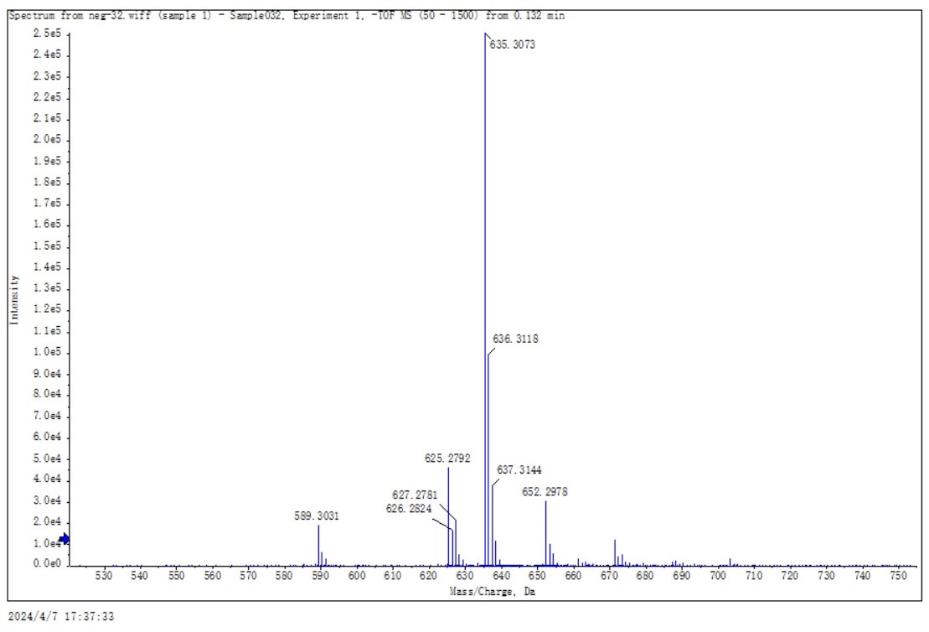


^1^H NMR of **Biotin-Cara-2** (CDCl_3_)

^13^C NMR of **Biotin-Cara-2** (CDCl_3_)

HRMS spectrum of **Biotin-Cara-2**


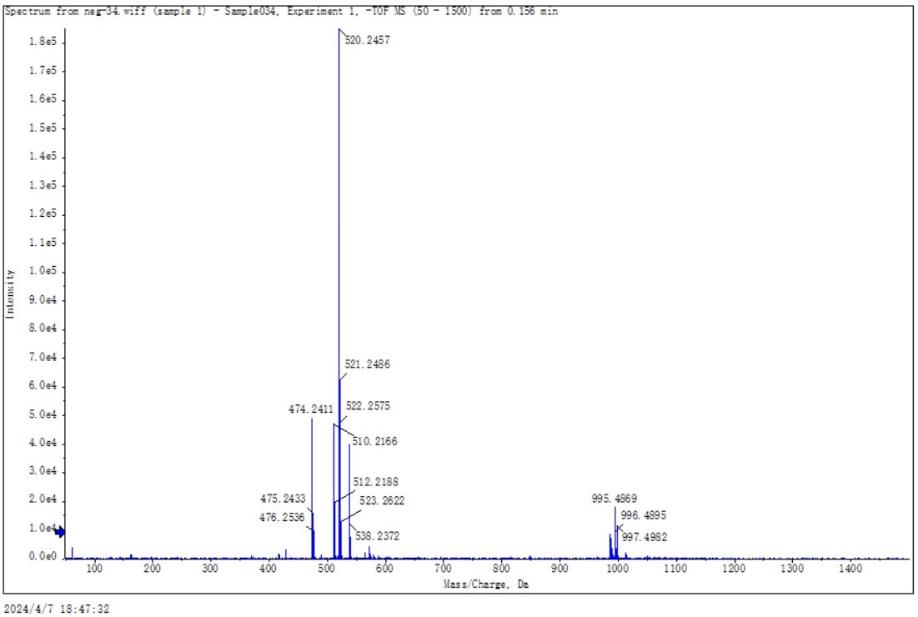


^1^H NMR of **Biotin-Cara-3** (CDCl_3_)

^13^C NMR of **Biotin-Cara-3** (CDCl_3_)

HRMS spectrum of **Biotin-Cara-3**


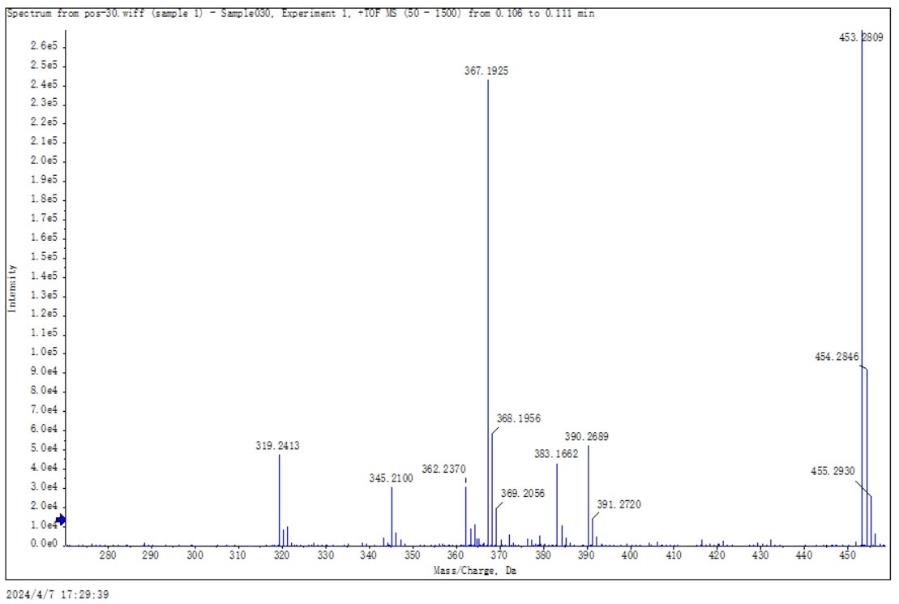


^1^H NMR of **Negative** (CDCl_3_)

^13^C NMR of **Negative** (CDCl_3_)

HRMS spectrum of **Negative**


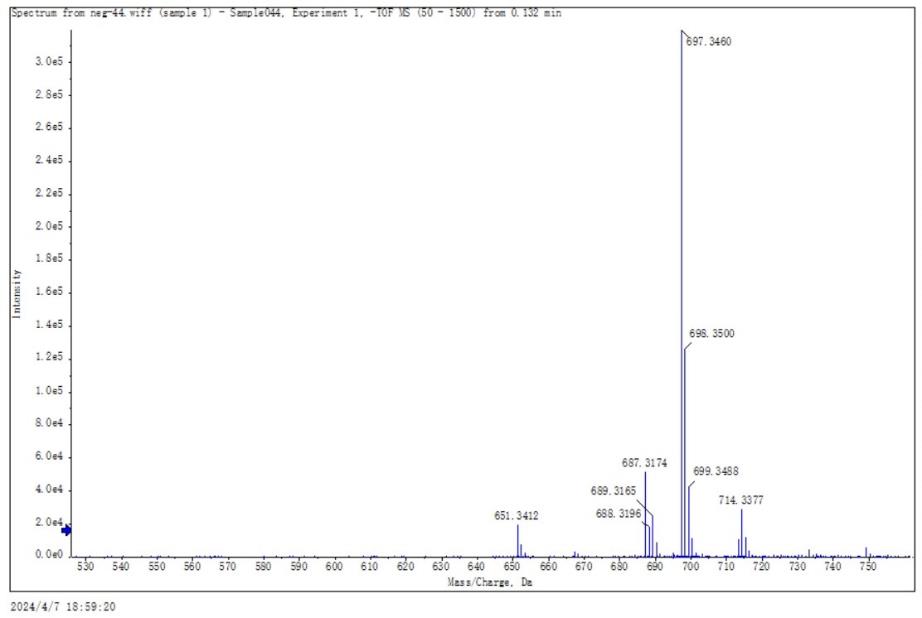


^1^H NMR of **PR-Cara** (CDCl_3_)

^13^C NMR of **PR-Cara** (CDCl_3_)

HRMS spectrum of **PR-Cara**


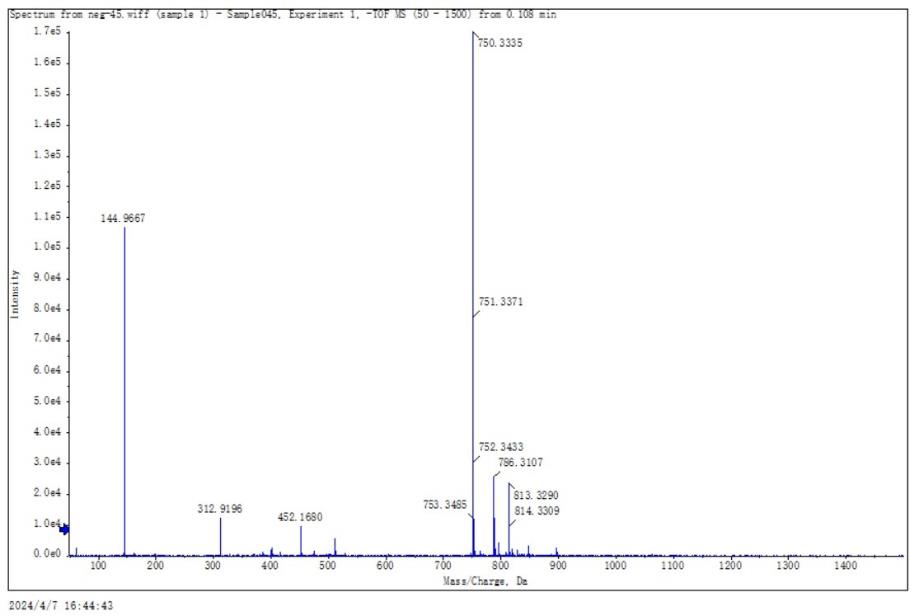


^1^H NMR of **CA-1** (CDCl_3_)

^13^C NMR of **CA-1** (CDCl_3_)

HRMS spectrum of **CA-1**


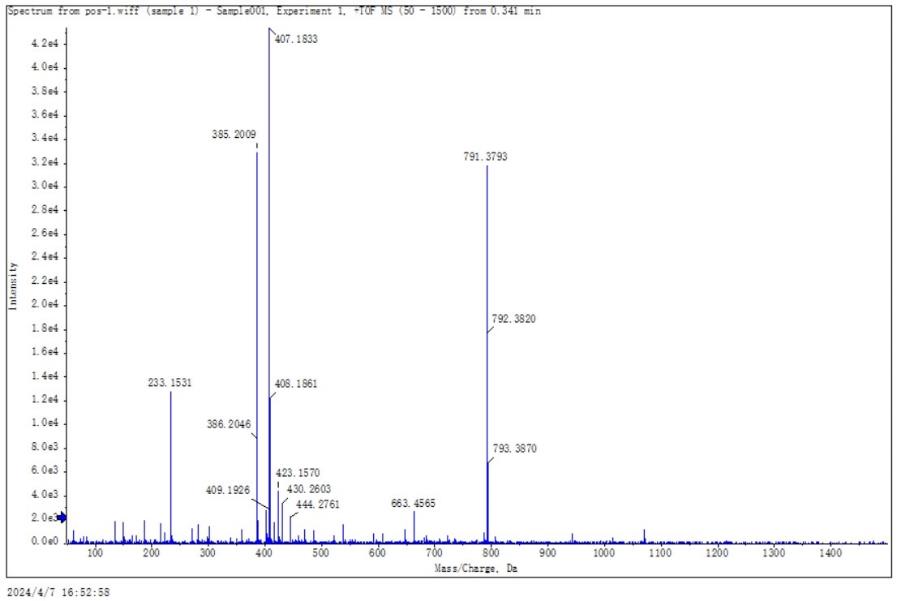


^1^H NMR of **CA-2** (CDCl_3_)

^13^C NMR of **CA-2** (CDCl_3_)

HRMS spectrum of **CA-2**


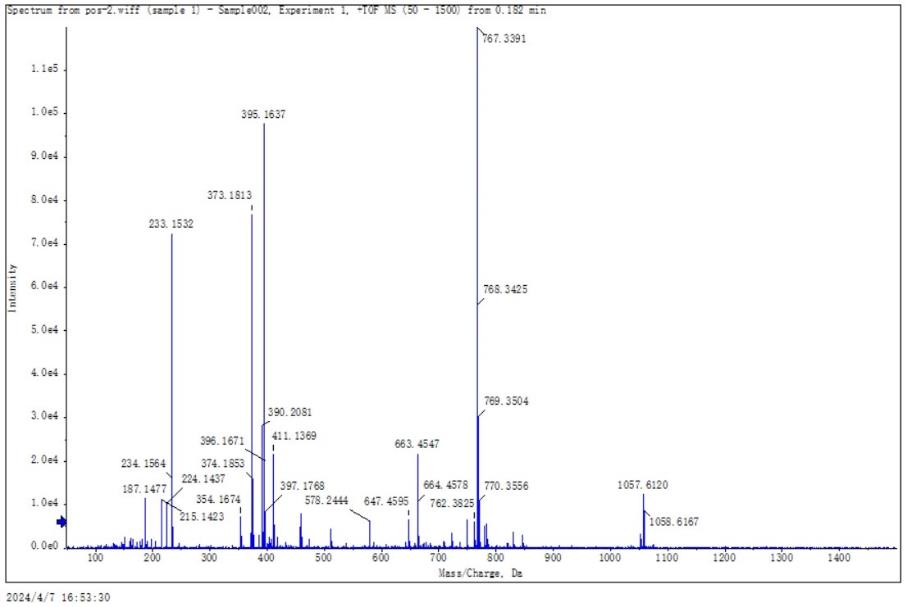


^1^H NMR of **CA-3** (CDCl_3_)

^13^C NMR of **CA-3** (CDCl_3_)

HRMS spectrum of **CA-3**


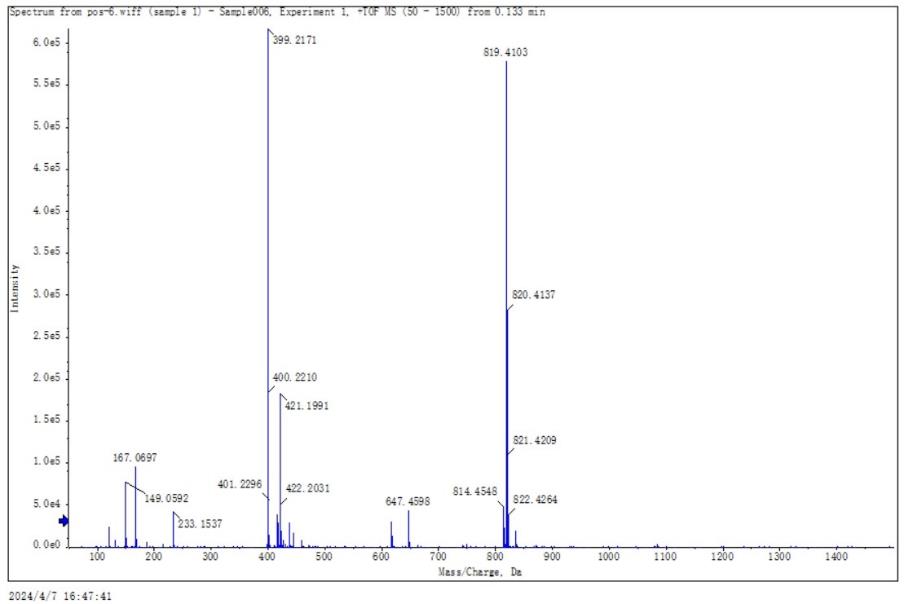


^1^H NMR of **CA-4** (CDCl_3_)

^13^C NMR of **CA-4** (CDCl_3_)

HRMS spectrum of **CA-4**


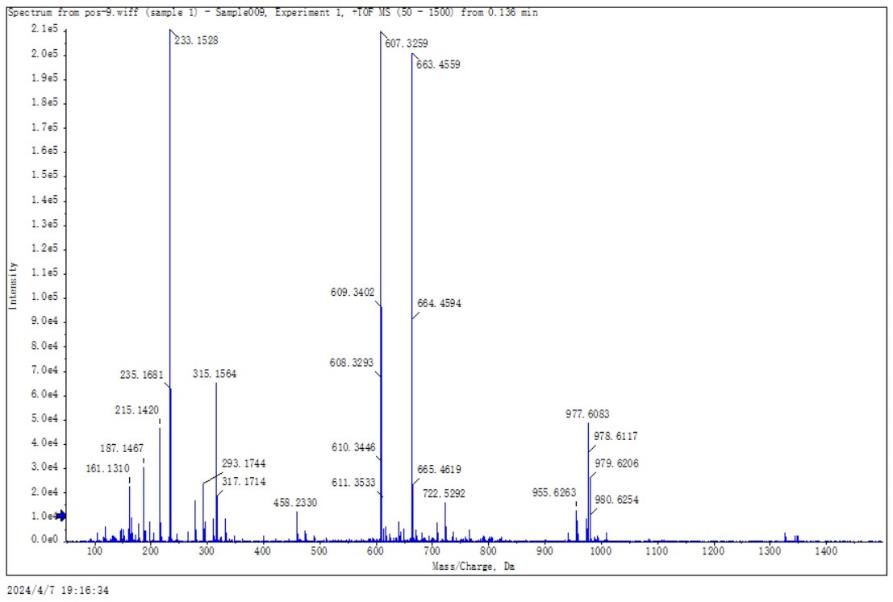


^1^H NMR of **CA-5** (CDCl_3_)

^13^C NMR of **CA-5** (CDCl_3_)

HRMS spectrum of **CA-5**


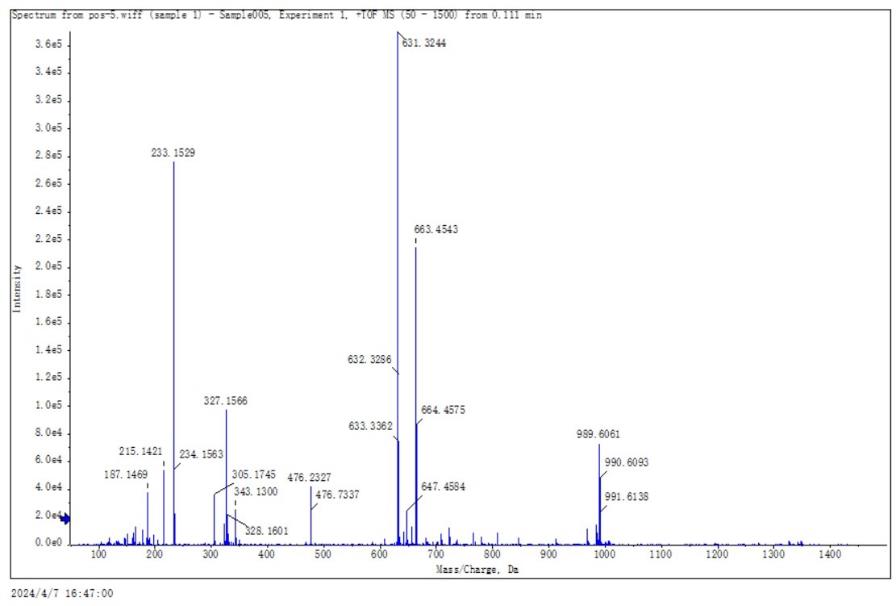


^1^H NMR of **CA-6** (CDCl_3_)

^13^C NMR of **CA-6** (CDCl_3_)

HRMS spectrum of **CA-6**


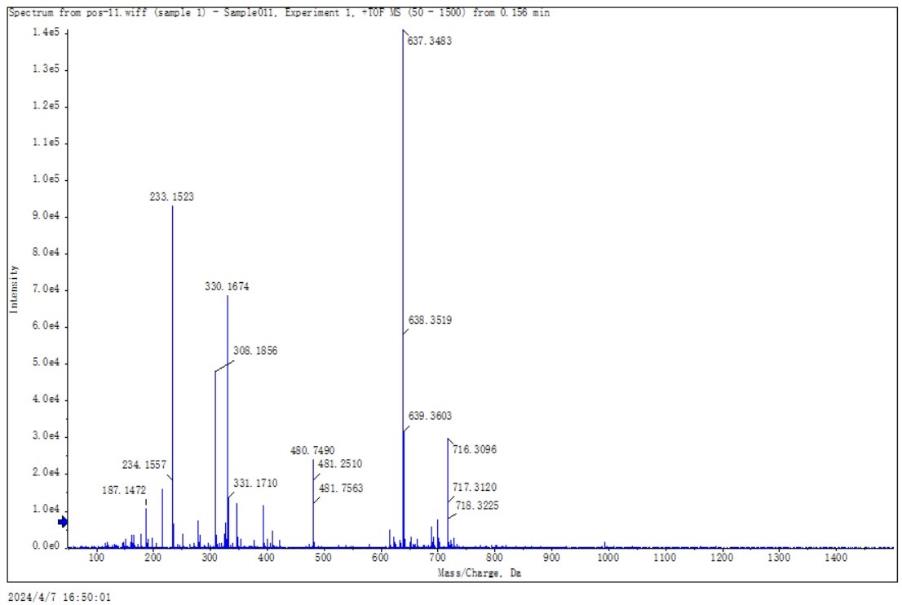


^1^H NMR of **CA-7** (CDCl_3_)

^13^C NMR of **CA-7** (CDCl_3_)

HRMS spectrum of **CA-7**


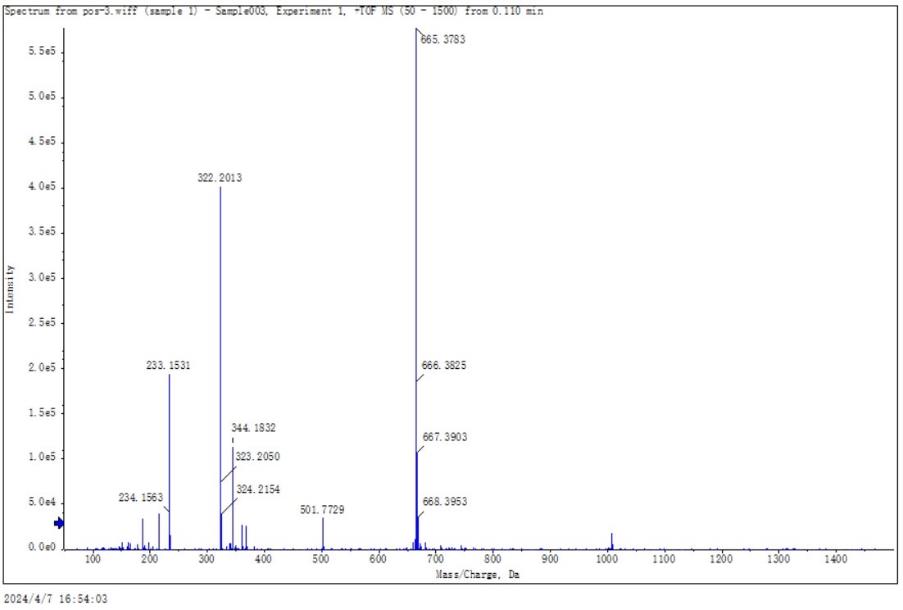


^1^H NMR of **CA-8** (CDCl_3_)

^13^C NMR of **CA-8** (CDCl_3_)

HRMS spectrum of **CA-8**


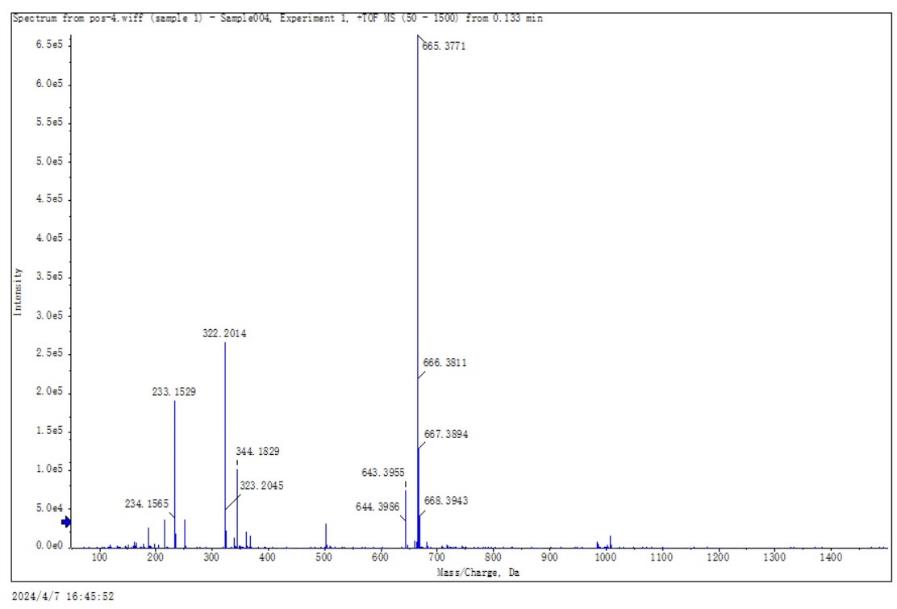


^1^H NMR of **CA-9** (CDCl_3_)

^13^C NMR of **CA-9** (CDCl_3_)

HRMS spectrum of **CA-9**


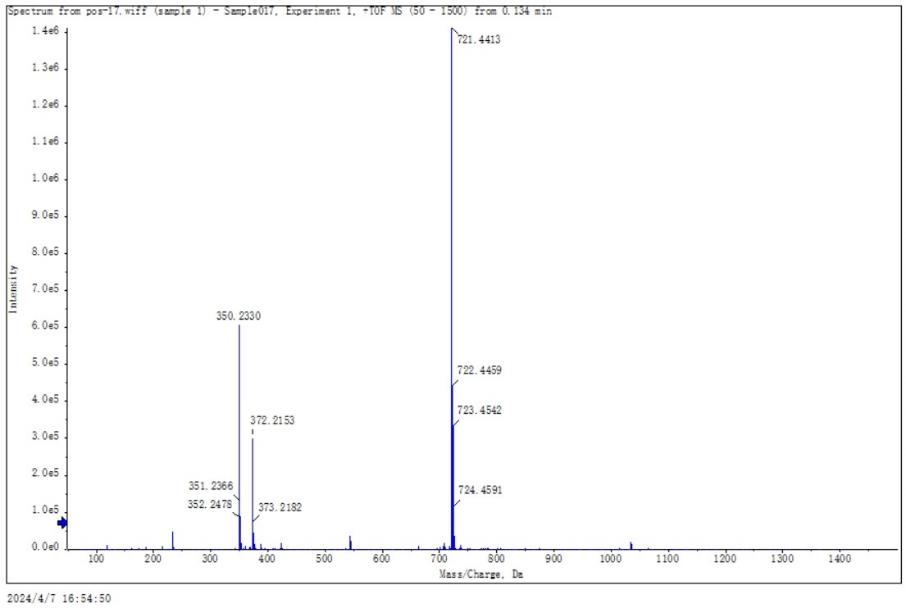


^1^H NMR of **CA-10** (CDCl_3_)

^13^C NMR of **CA-10** (CDCl_3_)

HRMS spectrum of **CA-10**


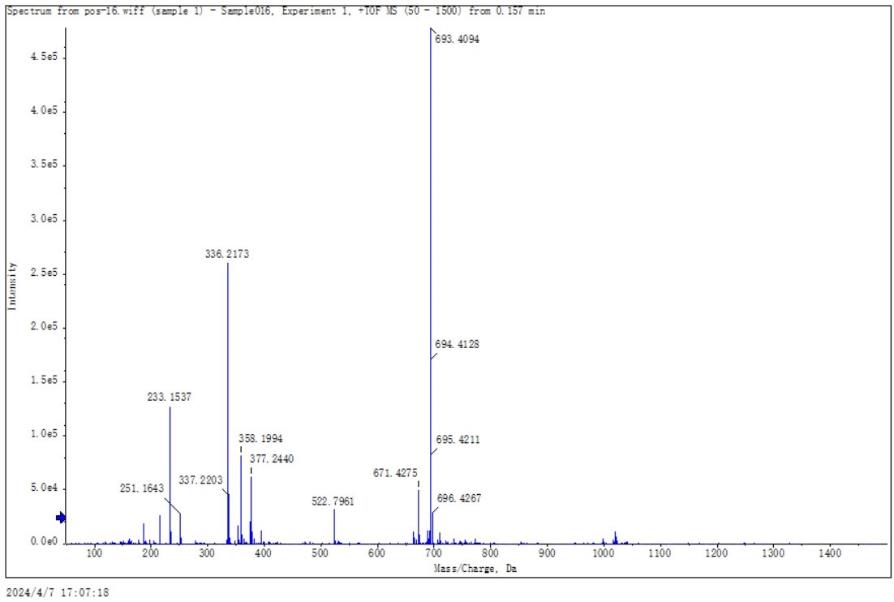


^1^H NMR of **CA-11** (CDCl_3_)

^13^C NMR of **CA-11** (CDCl_3_)

HRMS spectrum of **CA-11**


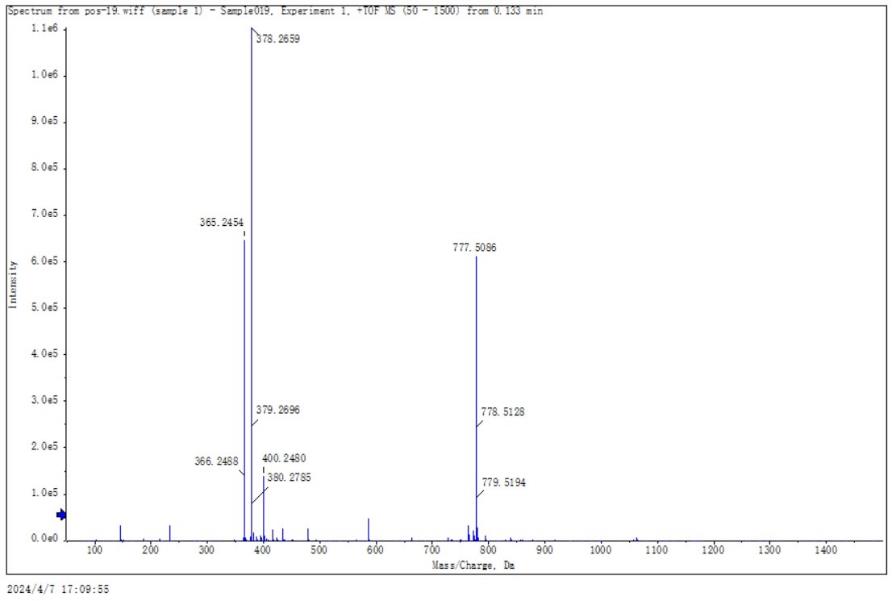


^1^H NMR of **CA-12** (CDCl_3_)

^13^C NMR of **CA-12** (CDCl_3_)

HRMS spectrum of **CA-12**


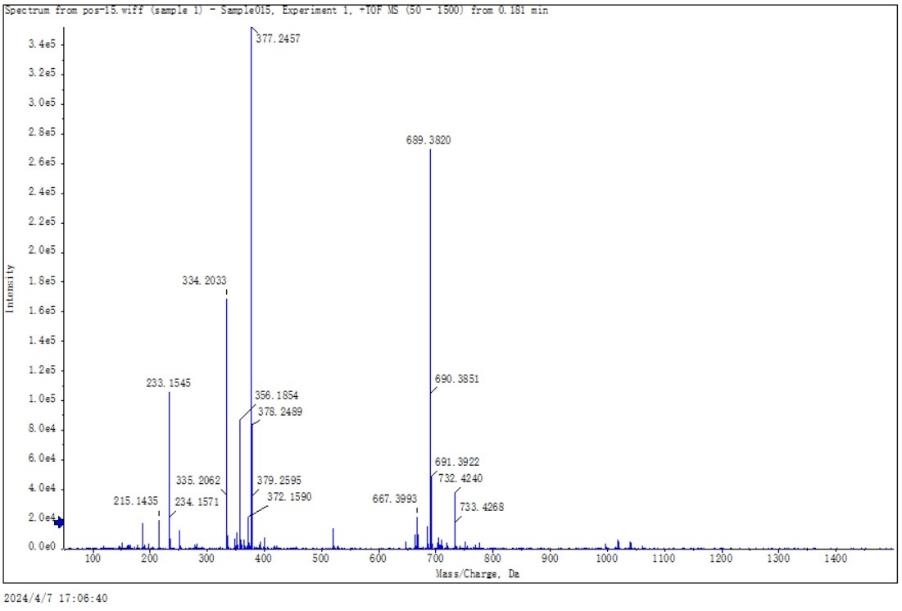


^1^H NMR of **CA-13** (CDCl_3_)

^13^C NMR of **CA-13** (CDCl_3_)

HRMS spectrum of **CA-13**


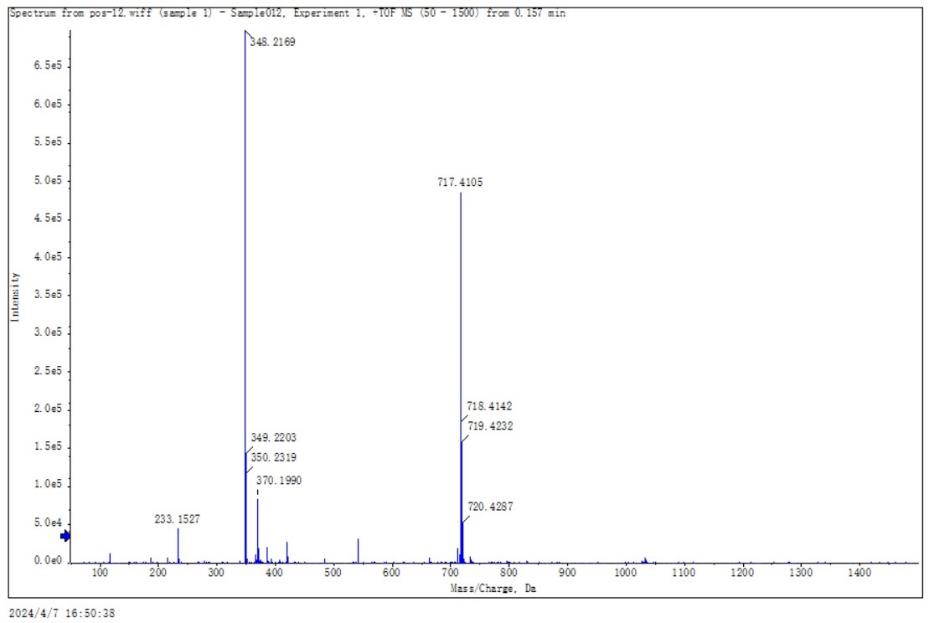


^1^H NMR of **CA-14** (CDCl_3_)

^13^C NMR of **CA-14** (CDCl_3_)

HRMS spectrum of **CA-14**


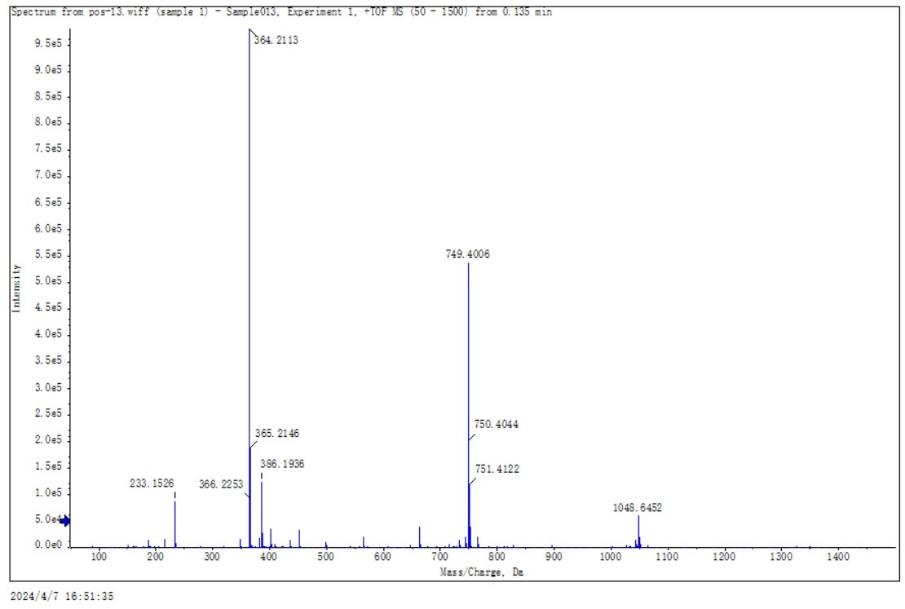


^1^H NMR of **CA-15** (CDCl_3_)

^13^C NMR of **CA-15** (CDCl_3_)

HRMS spectrum of **CA-15**


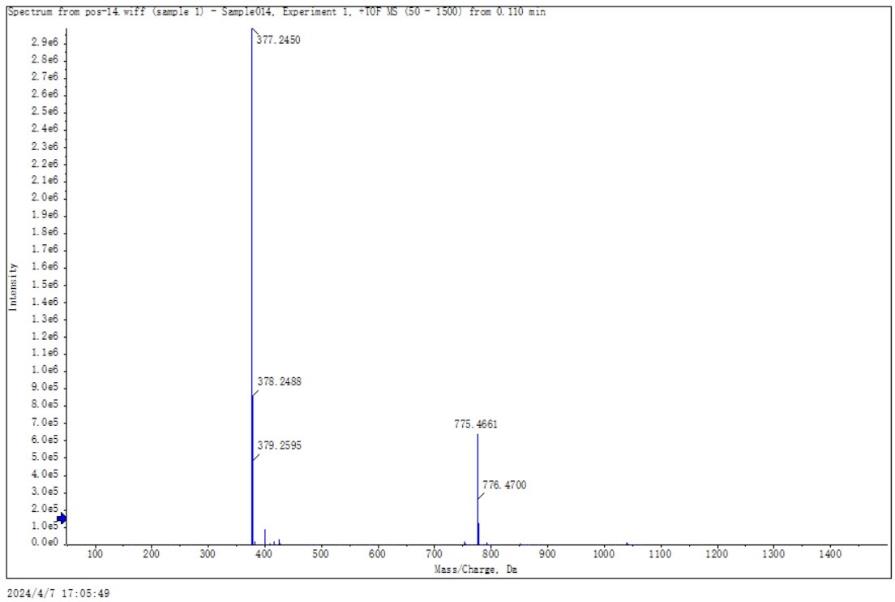


^1^H NMR of **CA-16** (CDCl_3_)

^^

^13^C NMR of **CA-16** (**CDCl_3_**)

HRMS spectrum of **CA-16**


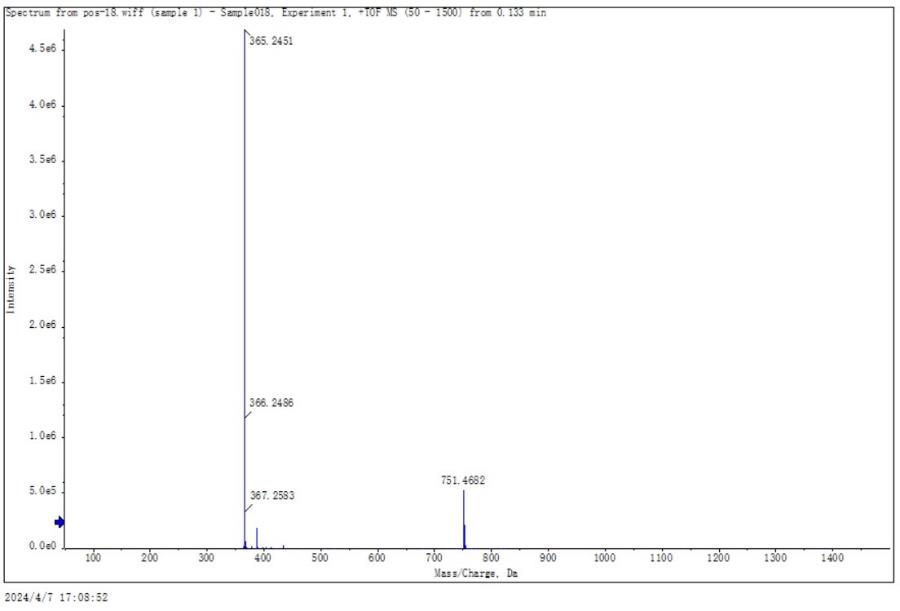


^1^H NMR of **CA-17** (CDCl_3_)

^13^C NMR of **II-17** (CDCl_3_)

HRMS spectrum of **CA-17**


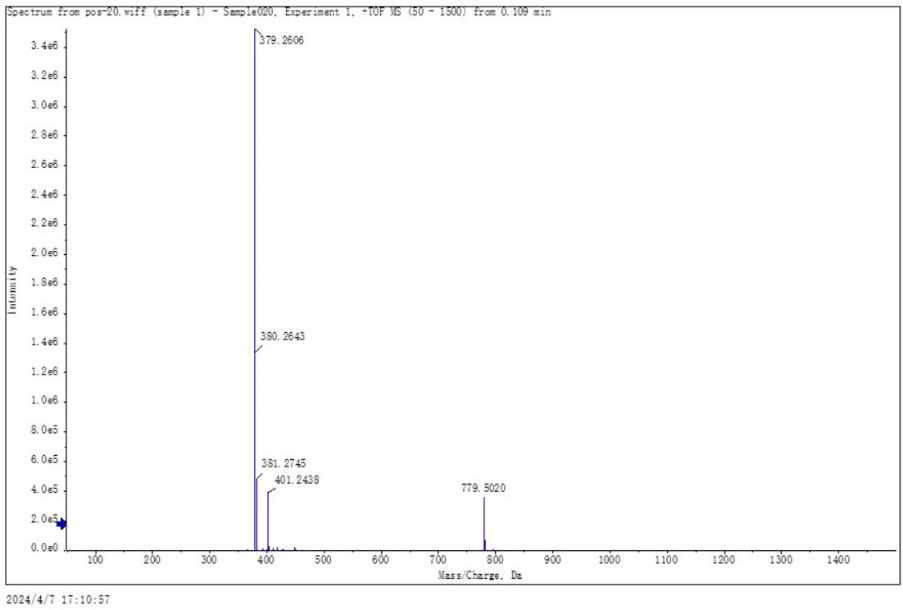


^1^H NMR of **CA-18** (CDCl_3_)

^13^C NMR of **CA-18** (CDCl_3_)

HRMS spectrum of **CA-18**


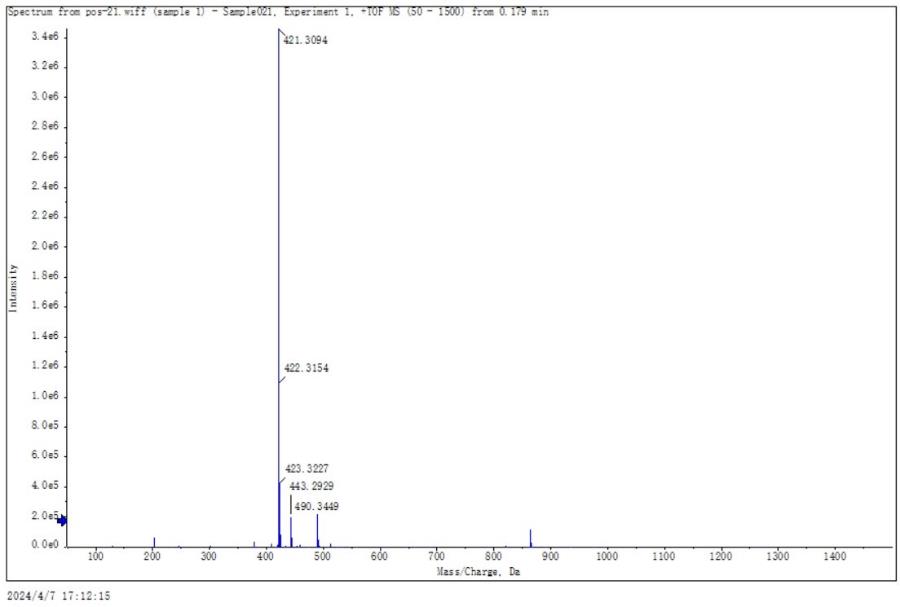


^1^H NMR of **CA-19** (CDCl_3_)

^13^C NMR of **CA-19** (CDCl_3_)

HRMS spectrum of **CA-19**


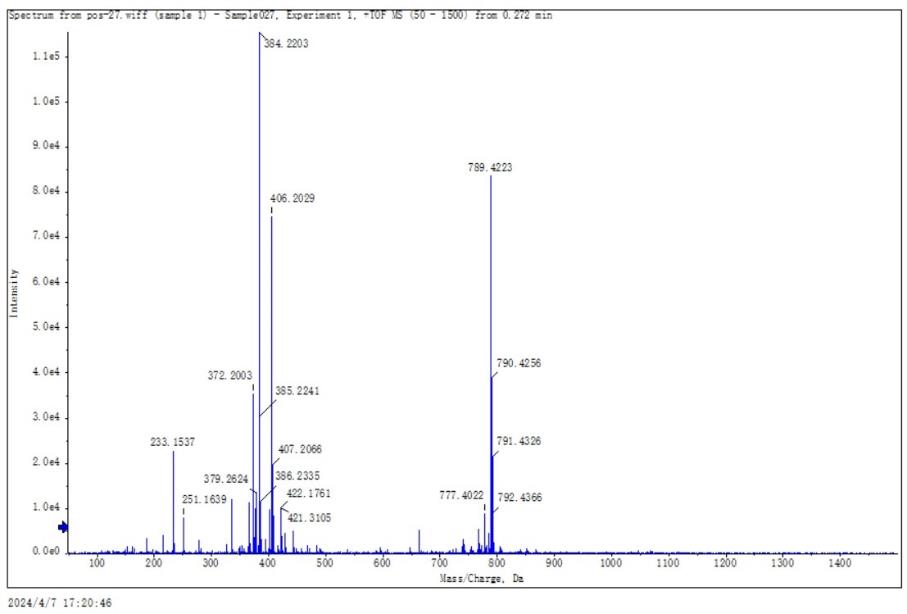


^1^H NMR of **CA-20** (CDCl_3_)

^13^C NMR of **CA-20** (CDCl_3_)

HRMS spectrum of **CA-20**


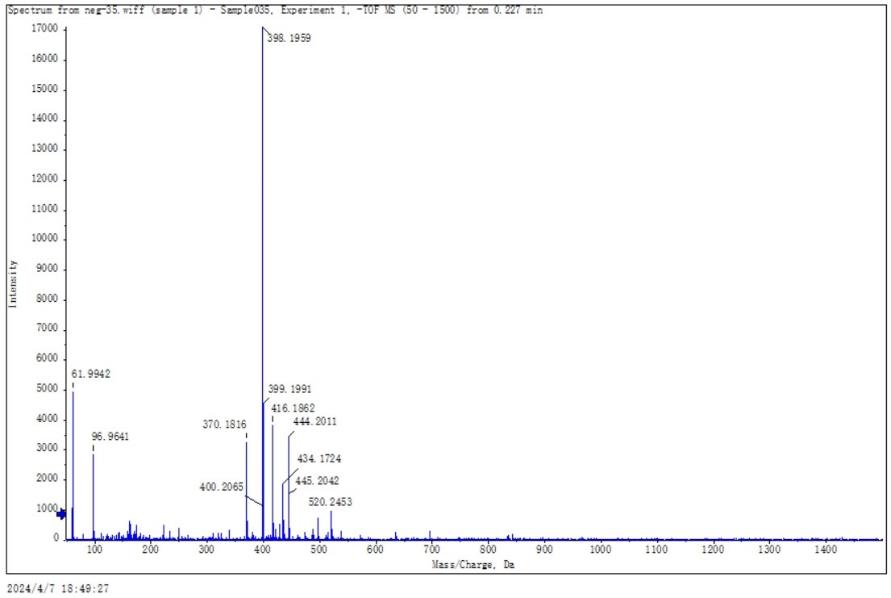


^1^H NMR of **CA-21** (CDCl_3_)

^13^C NMR of **CA-21** (CDCl_3_)

HRMS spectrum of **CA-21**


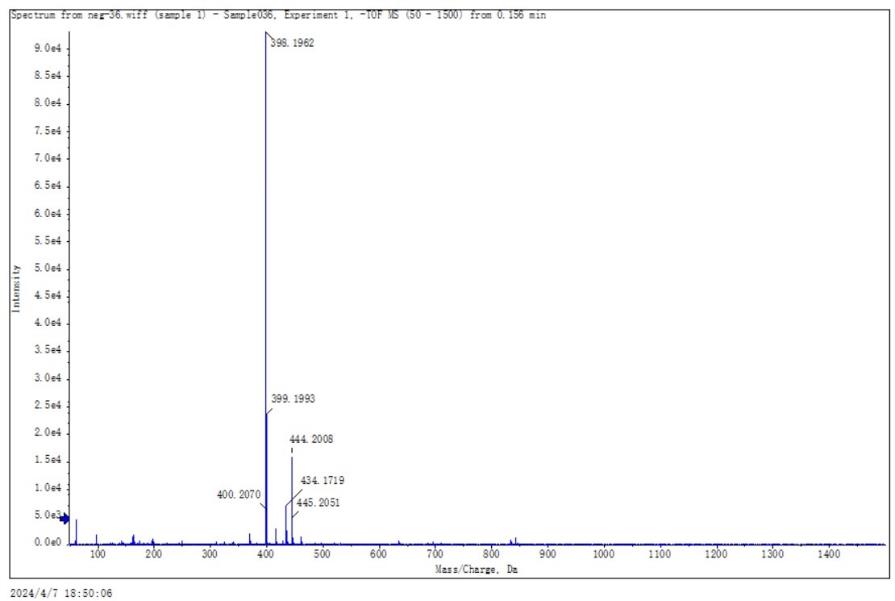


^1^H NMR of **CA-22** (CDCl_3_)

^13^C NMR of **CA-22** (CDCl_3_)

HRMS spectrum of **CA-22**


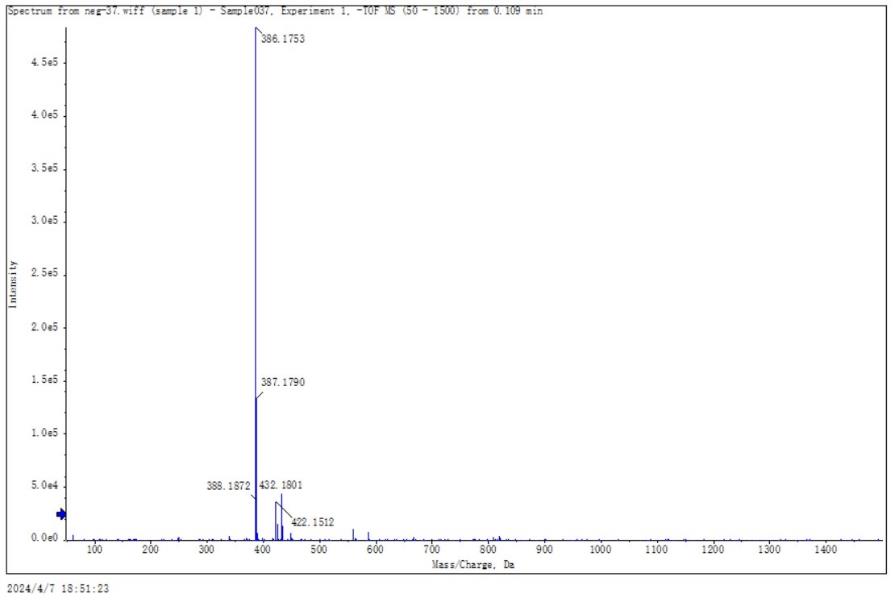


^1^H NMR of **CA-23** (CDCl_3_)

^13^C NMR of **CA-23** (CDCl_3_)

HRMS spectrum of **CA-23**


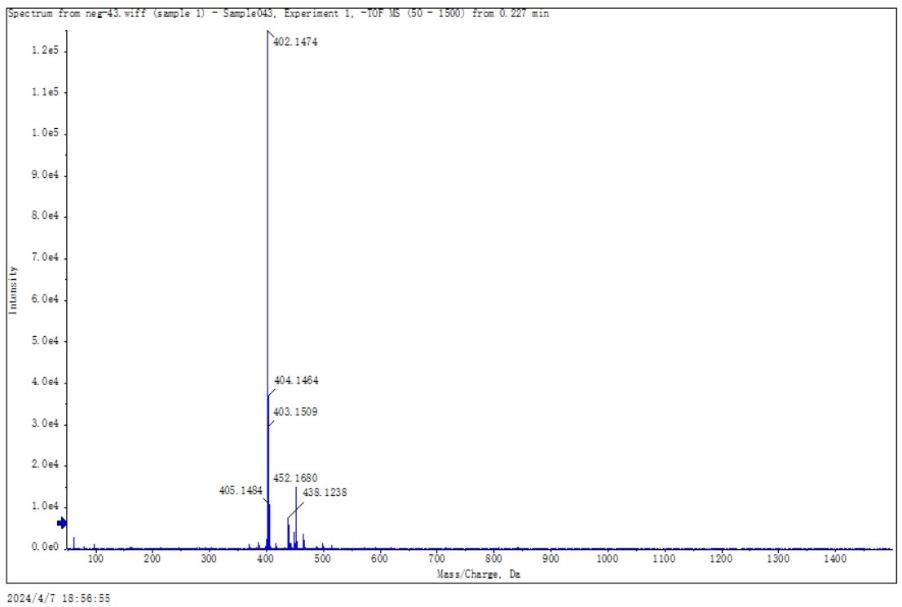


^1^H NMR of **CA-24** (CDCl_3_)

^13^C NMR of **CA-24** (CDCl_3_)

HRMS spectrum of **CA-24**


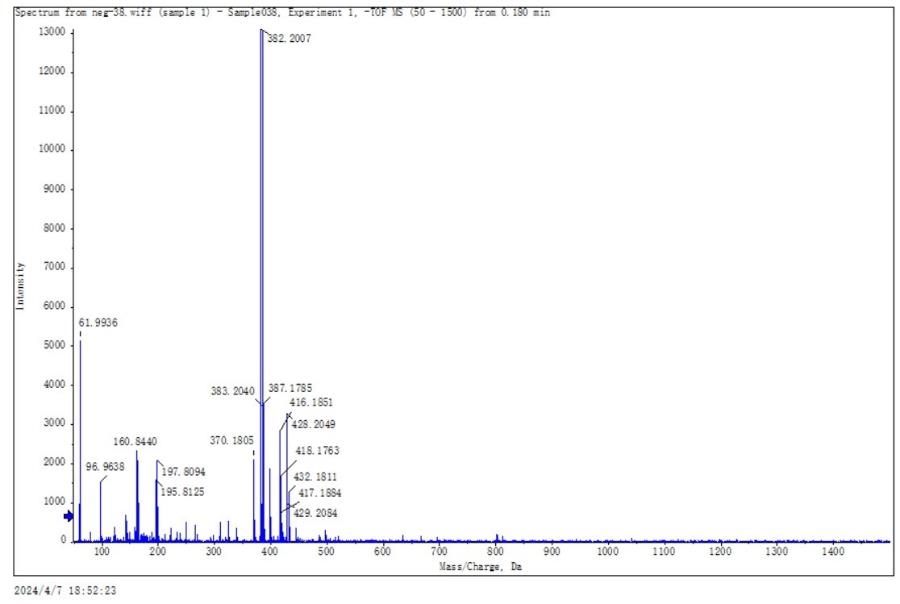


^1^H NMR of **CA-25** (CDCl_3_)

^13^C NMR of **CA-25** (CDCl_3_)

HRMS spectrum of **CA-25**


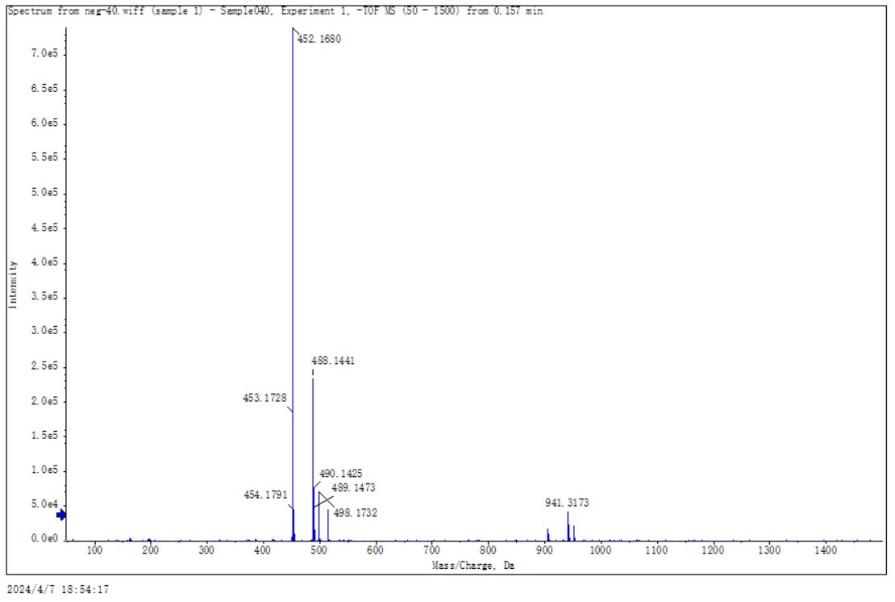


^1^H NMR of **CA-26** (CDCl_3_)

^13^C NMR of **CA-26** (CDCl_3_)

HRMS spectrum of **CA-26**


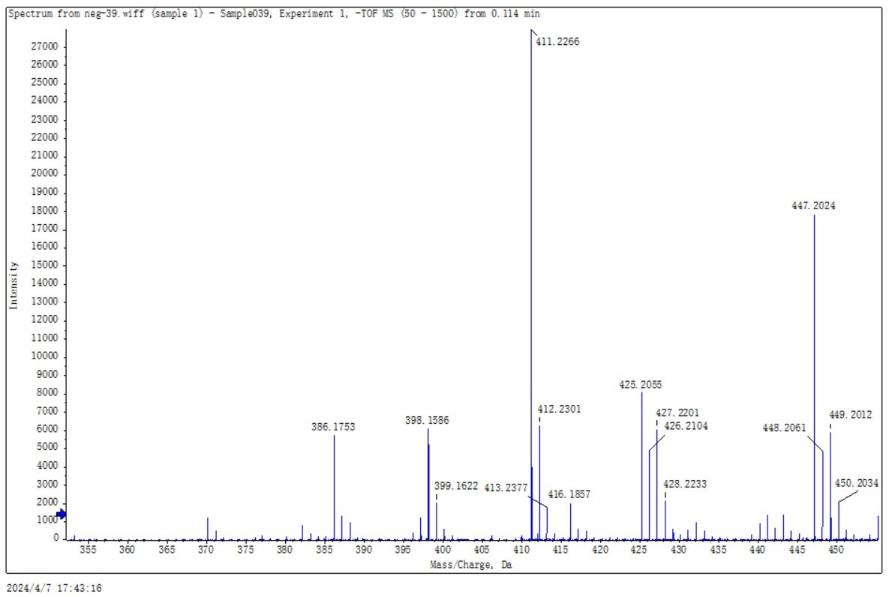


^1^H NMR of **CA-27** (CDCl_3_)

^13^C NMR of **CA-27** (CDCl_3_)

HRMS spectrum of **CA-27**


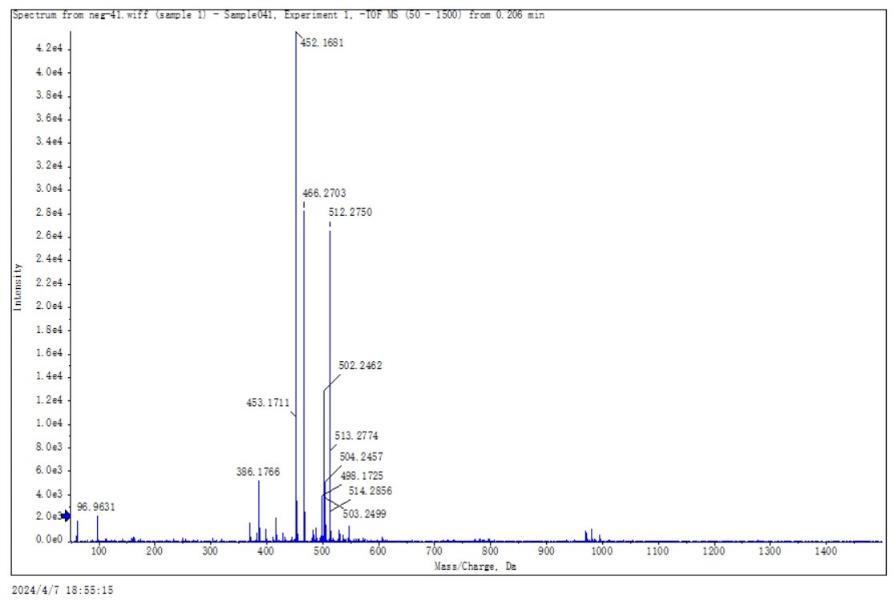


^1^H NMR of **CA-28**(CDCl_3_)

^13^C NMR of **CA-28** (CDCl_3_)

HRMS spectrum of **CA-28**


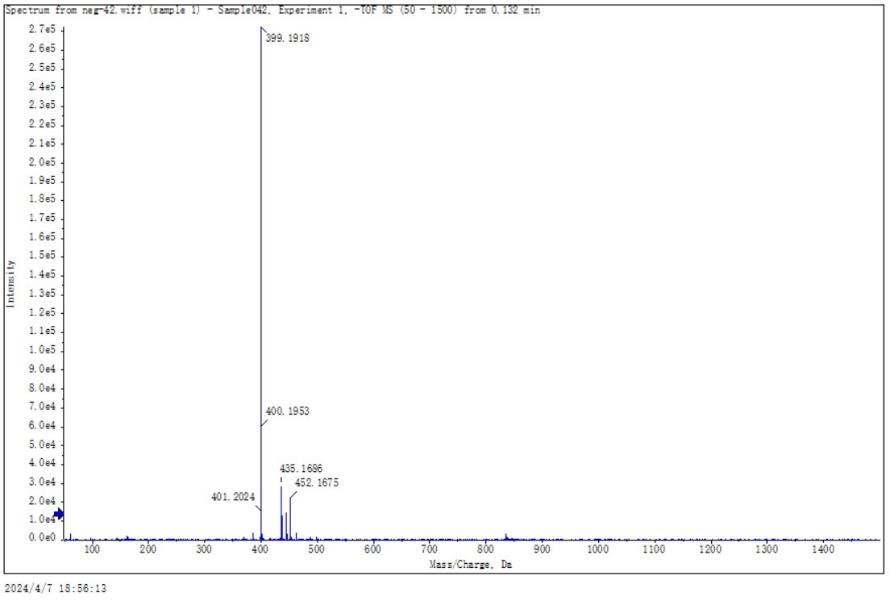


^1^H NMR of **CA-29** (CDCl_3_)

^13^C NMR of **CA-29** (CDCl_3_)

HRMS spectrum of **CA-29**


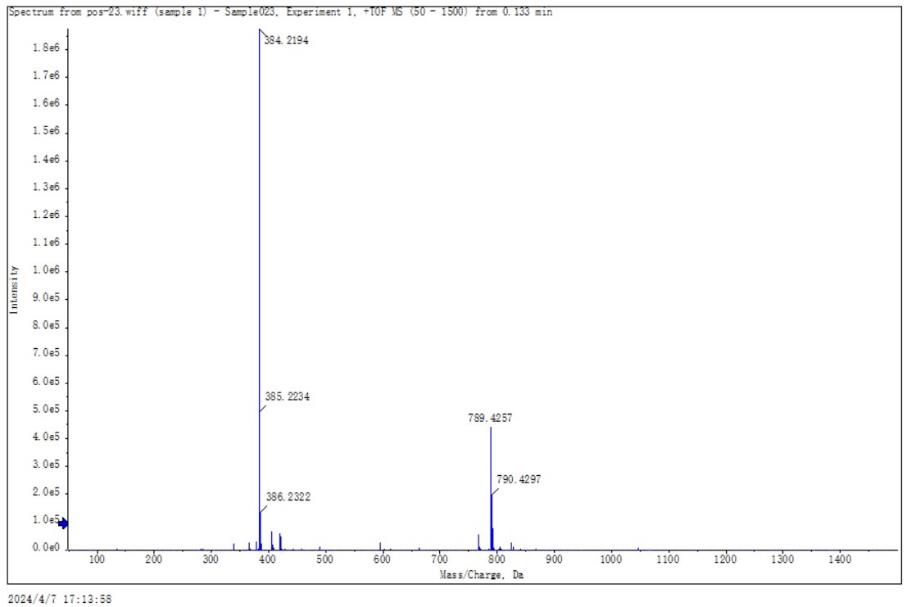


^1^H NMR of **CA-30** (CDCl_3_)

^13^C NMR of **CA-30** (CDCl_3_)

HRMS spectrum of **CA-30**


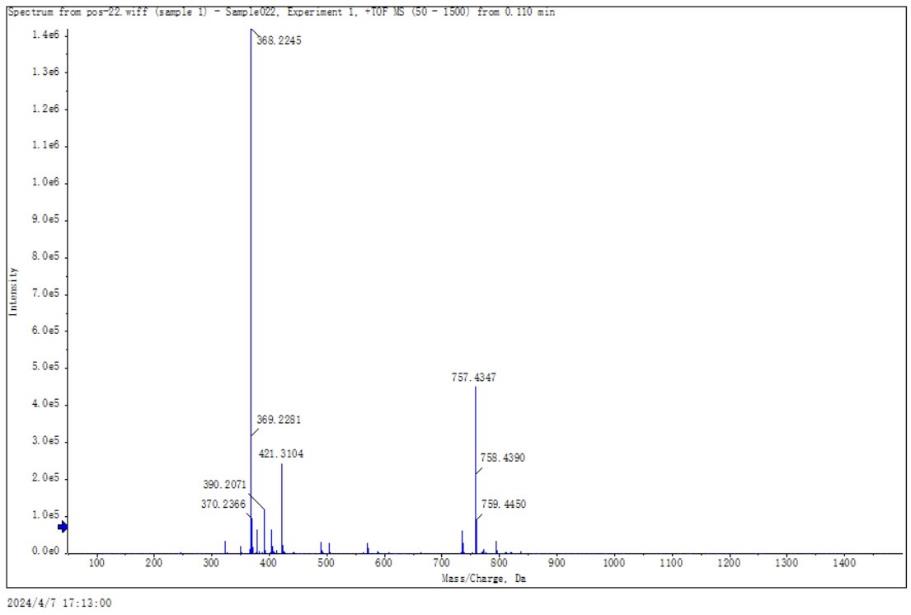


^1^H NMR of **CA-31** (CDCl_3_)

^13^C NMR of **CA-31** (CDCl_3_)

HRMS spectrum of **CA-31**


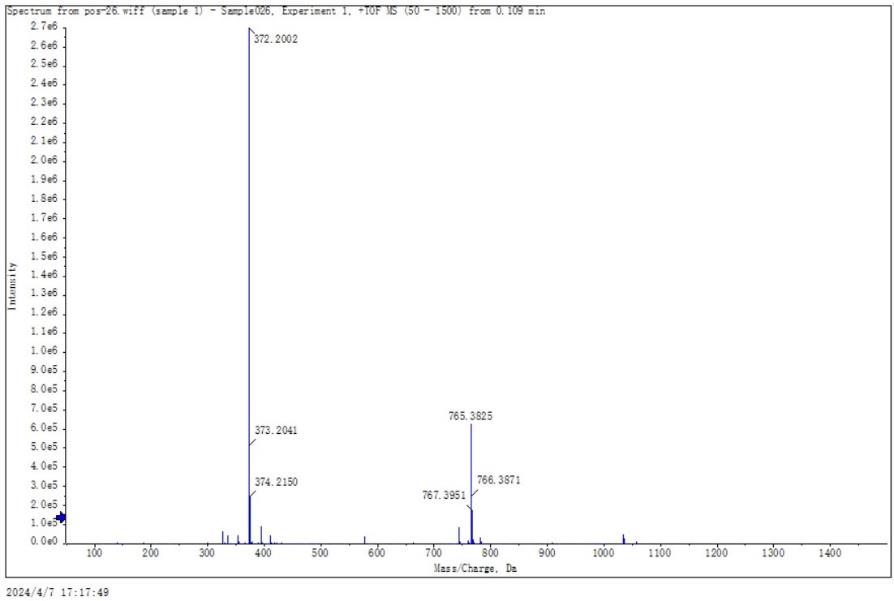


^1^H NMR of **CA-32** (CDCl_3_)

^13^C NMR of **CA-32** (CDCl_3_)

HRMS spectrum of **CA-32**


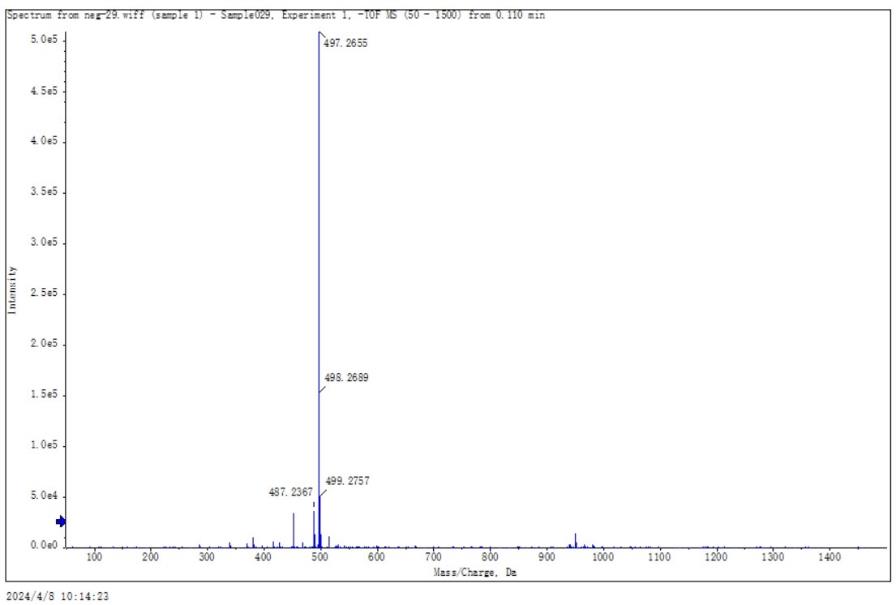


^1^H NMR of **CA-33** (CDCl_3_)

^13^C NMR of **CA-33** (CDCl_3_)

HRMS spectrum of **CA-33**


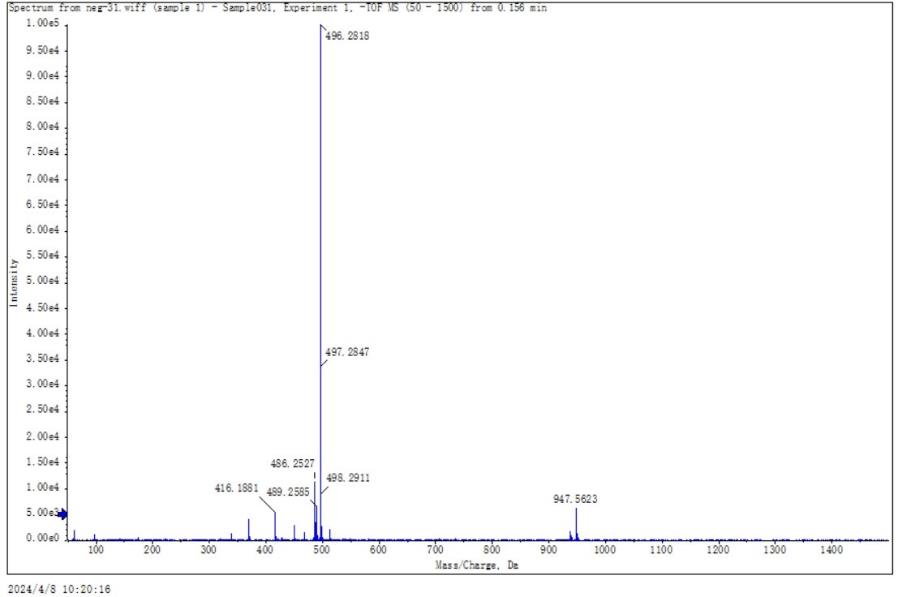


^1^H NMR of **CA-34** (CDCl_3_)

^13^C NMR of **CA-34** (CDCl_3_)

HRMS spectrum of **CA-34**


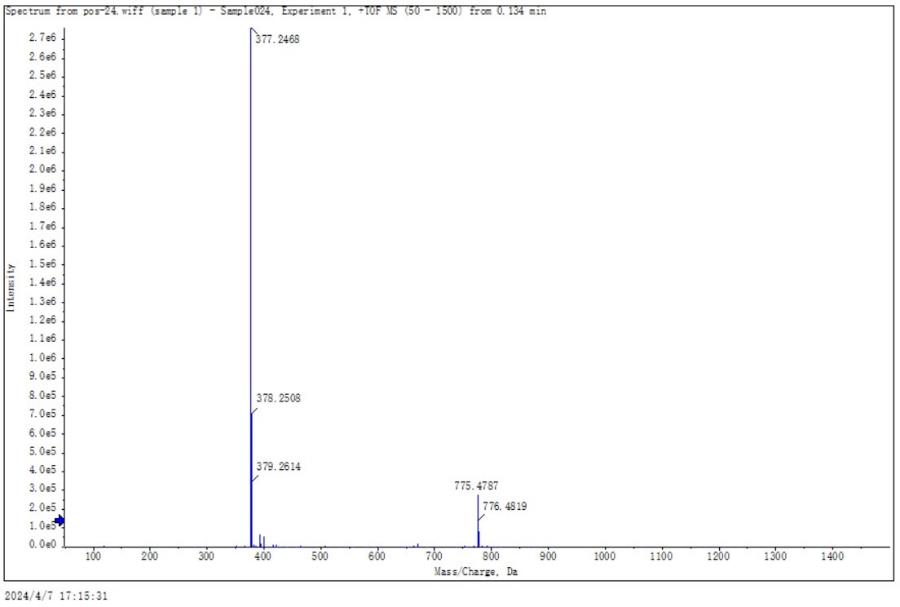


^1^H NMR of **CA-35** (CDCl_3_)

^13^C NMR of **CA-35** (CDCl_3_)

HRMS spectrum of **CA-35**


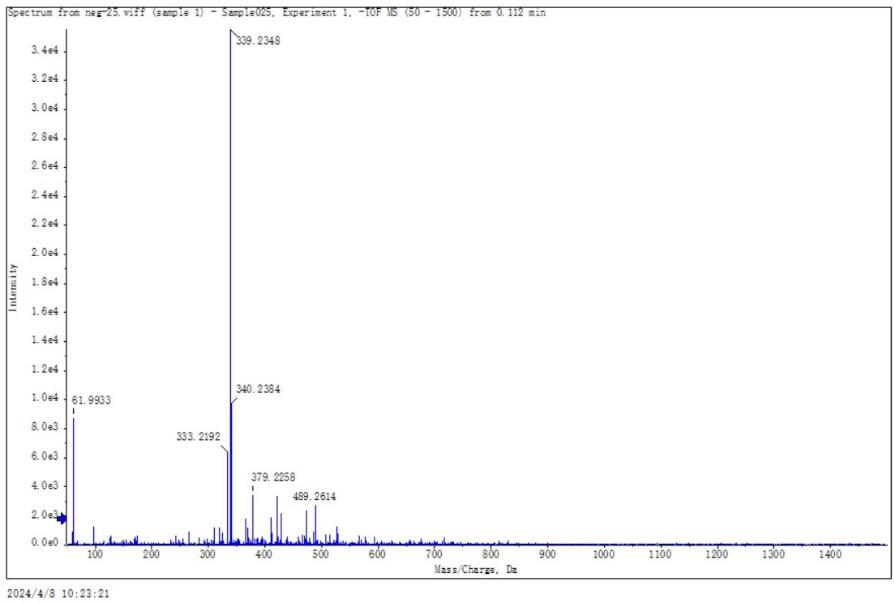


^1^H NMR of **CA-36** (CDCl_3_)

^13^C NMR of **CA-36** (CDCl_3_)

HRMS spectrum of **CA-36**


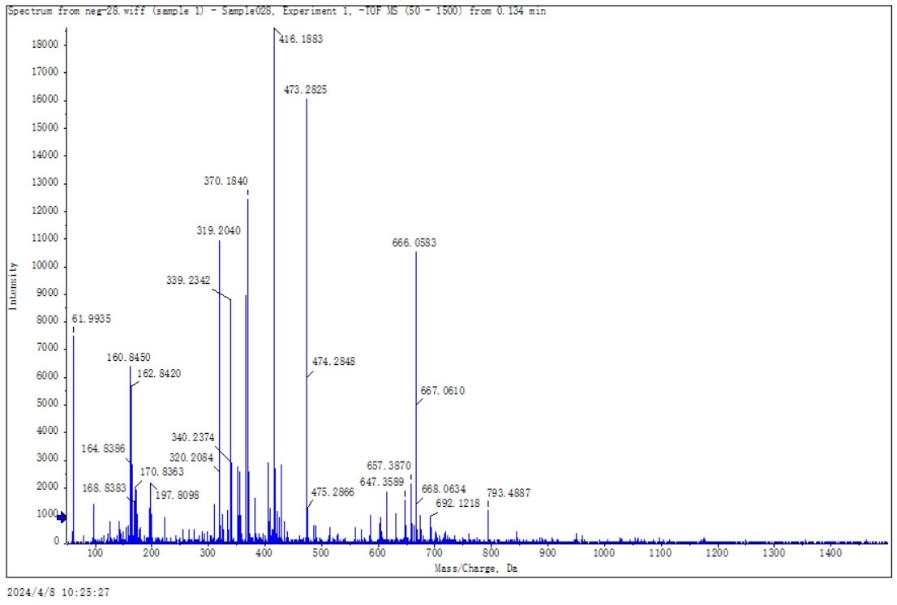

Supplement: Supplementary file 1 — Supporting Information: Figure S1‐S10, Table S1‐S4 and synthetic schemes and methods of compounds in this paper. [file MCO2-6-e70145-s001.docx]
